# Clinical, pathological, and laboratory diagnoses of diseases of harbour porpoises (*Phocoena phocoena*), live stranded on the Dutch and adjacent coasts from 2003 to 2016

**DOI:** 10.1186/s13567-019-0706-3

**Published:** 2019-10-30

**Authors:** Cornelis E. van Elk, Marco W. G. van de Bildt, Peter R. W. A. van Run, Paulien Bunskoek, Jolanda Meerbeek, Geoffrey Foster, Albert D. M. E. Osterhaus, Thijs Kuiken

**Affiliations:** 1 Department of Viroscience, Erasmus Medical Center, Wytemaweg 80, 3015 CN Rotterdam, The Netherlands; 2 Dolfinarium Harderwijk, Zuiderzeeboulevard 22, 3841 WB Harderwijk, The Netherlands; 3 SOS Dolfijn, Valkenhof 61, 3862 LL Nijkerk, The Netherlands; 4 SRUC Veterinary Services, Inverness, IV2 5NA UK; 5 Research Center for Emerging Infections and Zoonoses, University of Veterinary Medicine, Bünteweg 17, 30559 Hannover, Germany

## Abstract

Harbour porpoises (*Phocoena phocoena*) in the North Sea live in an environment heavily impacted by humans, the consequences of which are a concern for their health. Autopsies carried out on stranded harbour porpoises provide an opportunity to assess health problems in this species. We performed 61 autopsies on live-stranded harbour porpoises, which died following admission to a rehabilitation centre between 2003 and 2016. The animals had stranded on the Dutch (*n* = 52) and adjacent coasts of Belgium (*n* = 2) and Germany (*n* = 7). We assigned probable causes for stranding based on clinical and pathological criteria. Cause of stranding was associated in the majority of cases with pathologies in multiple organs (*n* = 29) compared to animals with pathologies in a single organ (*n* = 18). Our results show that the three most probable causes of stranding were pneumonia (*n* = 35), separation of calves from their mother (*n* = 10), and aspergillosis (*n* = 9). Pneumonia as a consequence of pulmonary nematode infection occurred in 19 animals. Pneumonia was significantly associated with infection with *Pseudalius inflexus, Halocercus* sp., and *Torynurus convolutus* but not with *Stenurus minor* infection. Half of the bacterial pneumonias (6/12) could not be associated with nematode infection. Conclusions from this study are that aspergillosis is an important probable cause for stranding, while parasitic infection is not a necessary prerequisite for bacterial pneumonia, and approximately half of the animals (29/61) probably stranded due to multiple causes. An important implication of the observed high prevalence of aspergillosis is that these harbour porpoises suffered from reduced immunocompetence.

## Introduction

Biodiversity is in sharp decline due to increasing human pressures on the environment. The marine environment is no exception and vertebrate population abundance loss in the oceans has been estimated at 36% between 1970 and 2012 [1]. Therefore, there is justifiable concern for the conservation of marine species and ecosystems in areas where humans have a large impact. This includes the harbour porpoise (*Phocoena phocoena*) living in the North Sea, an environment heavily influenced by human activities.

Anthropogenic activities in the North Sea lead to chemical pollution [2], noise pollution [3], and depleted fish populations [4], which all may affect harbour porpoises. Firstly, they are vulnerable to chemical pollution because they bioaccumulate and biomagnify lipophilic chemical pollutants [2]. Multiple investigations have found indications for the negative effect of these chemical pollutants on the immune system of harbour porpoises in the North Sea and adjacent waters [5–8]. Secondly, harbour porpoises are vulnerable to noise pollution because their hunting and communication are largely dependent on acoustic signals. Thirdly, they are vulnerable to fishing activities because they drown due to accidental capture in fishing gear [9] and because harbour porpoises partly depend on fish species that are also targeted by human fisheries [10].

Historic observations on the abundance of harbour porpoises in the North Sea suggest it is a vulnerable population. Harbour porpoises were abundant in Dutch coastal waters until the early fifties of the last century, went nearly extinct in the seventies and eighties, but showed a strong population increase in the decades thereafter [11]. The reasons for these fluctuations in abundance are largely unknown, although chemical pollution and fisheries bycatch have been implicated as causes for the population decline [12].

Previous investigations, among harbour porpoises stranded and bycaught around the North Sea between 1990 and 2000, have shown that the top three (probable) causes of mortality are bycatch, bronchopneumonia (bacterial, parasitic or a combination of the two) and starvation (mainly of neonates) [13–16].

It is unknown, however, whether causes of mortality have changed since 2000 or if these causes of mortality were different around the Dutch coast compared to those other regions of the North Sea. Moreover, there is no consensus on the impact of parasitic lung infections on the health of harbour porpoises. Some researchers regard pulmonary parasitic infections as a primary cause of death [13, 15, 16], or as the trigger for secondary and lethal bacterial pneumonias [14, 16], while others have observed heavy infections without apparent health effects [15, 17].

Our goal, therefore, was to establish the probable causes for stranding of harbour porpoises around the Dutch coast in comparison with previous surveys [13–16], and to evaluate the role of parasitic lung infections as a cause of pneumonia.

Autopsies were performed on harbour porpoises that stranded alive on the coasts of the Netherlands or neighbouring countries between 2003 and 2016, were rescued, but despite rehabilitation efforts died or had to be euthanized, while in captivity. The advantage of this set up was that we had clinical and pathological data of these animals, and that carcasses were always fresh.

Our main findings showed that cause of stranding was associated mostly with alterations in multiple organs (*n* = 29) rather than alterations in a single organ (*n* = 18). Nematode infections resulted in pneumonia in 19 animals and was significantly associated with infection with *Pseudalius inflexus*, *Halocercus* sp., and *Torynurus convolutus* but not with infection with *Stenurus minor*. Half of the bacterial pneumonias (6/12) occurred independently of nematode infection. We observed aspergillosis in an unprecedented high prevalence 14.7% (*n* = 61). These results suggest the immunocompetence of our sample of harbour porpoises was reduced compared to the samples of harbour porpoises in previous surveys [13–16].

## Materials and methods

### Rescue and rehabilitation of live‑stranded cetaceans at SOS Dolfijn

Since 1967, small cetaceans—mainly harbour porpoises—that strand alive along the Dutch, Belgian and German coasts have been rescued and rehabilitated at the Dolfinarium Harderwijk (Harderwijk, The Netherlands) and subsequently released into the wild. Since 2004, this activity was operated by an independent foundation, SOS Dolfijn, at the same site. Admission and rehabilitation of live stranded wild harbour porpoises at the SOS Dolphin Foundation was authorized by the government of the Netherlands (permit number FF/75/2012/036). SOS Dolfijn had two 50 m^3^ pools with fresh water to which sodium chloride was added. In the first period of rehabilitation, animals were observed round the clock and standard parameters were recorded, including respiration rate, cramps, food intake and defaecation. In addition, other potentially relevant observations were recorded, including swimming behaviour and alertness. As an animal improved, the level of observation and care diminished to a minimum of 9 h/day.

### Age determination of autopsied harbour porpoises

Age classes were defined according to the following criteria [18]: neonates, animals less than a week old based on remains of umbilicus or time of year found (June, July), body weight up to 11 kg and body length up to 90 cm; juveniles, immature gonads (testis weight < 100 g each for males; absence of corpus luteum or corpus albicans on ovaries for females) and body length < 130 cm for males and < 145 cm for females; adults, mature gonads (testis weight > 100 g each for males and presence of corpus luteum, corpus albicans or follicle > 1 cm diameter for females) or with a body length > 130 cm for males or > 145 cm for females. Ages of juveniles were estimated by comparing length at admission with published age length data [18] and assumed date of birth on the first of July [19].

### Autopsy and histology

Autopsies were performed according to a standard protocol [20], and by the same pathologists. The following tissues were sampled for histology: adrenal gland, bronchus, cerebellum, cerebrum, colon, duodenum, oesophagus, forestomach, fundic stomach, gonads, heart, jejunum, kidney, liver, lung, mesenteric lymph node, muscle, pancreas, pulmonary lymph node, pyloric stomach, skin, spleen, thymus, thyroid, trachea, tracheobronchial lymph node, and urinary bladder. Additional samples were taken of tissues with gross lesions. Tissue samples were fixed in 10% neutral-buffered formalin, routinely processed, and embedded in paraffin. The 3-μm-thick sections were mounted on glass slides and stained with haematoxylin and eosin (HE) for light microscopy.

### Organ weights and sizes

Weight of body, left lung, spleen, liver, kidney, gonads, brain, adrenal, heart and width ratio of left cardiac ventricular wall and right cardiac ventricular wall, and weight ratio of left and right lung were compared to body length (straight line from tip of snout to fluke notch). For each comparison, the best fit line with the highest R^2^ (coefficient of determination) was plotted by use of Excel (Microsoft office; linear, exponential, polynomial, logarithmic, or power). To investigate if extreme variation from the mean contained relevant information, the 5% most extreme values (high or low) were checked for diagnoses and probable causes of stranding for each organ or ratio. Whether a value was extreme was determined by the difference between measured and predicted or absolute value. For relationships with an R^2^ > 0.50 (indicating body length was an independent with a strong predictive value for the variable measured) predicted R^2^ values were chosen. Absolute measured values were chosen in case R^2^ < 0.50 (indicating body length had weak predictive value for organ weight).

### Bacteriology

For bacteriological examination of animals displaying gross or histological lesions suggestive of bacterial disease, samples of lung, kidney, liver, spleen, pulmonary lymph node, and adrenal gland were frozen at −20 °C and after thawing, cultured according to a standard protocol. Briefly, each tissue was plated on Columbia sheep blood agar (CSBA) (Oxoid, Basingstoke, UK), MacConkey agar (Oxoid), and Farrell’s medium [21], which was set up specifically for the recovery of *Brucella ceti* [22]. A chocolate agar (CA) plate (Oxoid) was included for lung and pulmonary lymph node. CSBA, CA and Farrell’s plates were incubated at 37 °C aerobically plus 5% CO_2_ and examined daily for 14 days, whereas MacConkey agar plates were incubated aerobically without added CO_2_ at 37 °C for 48 h. Isolates were identified based on Gram stain reaction and morphology, gaseous requirements and a range of phenotypic tests, dependent upon the suspected identity of each isolate. Phenotypic tests included classical methods and commercial API identification kits (BioMerieux, Basingstoke, UK), which included analytical profiles for bacterial species from marine mammals established in-house.

### Parasitology

Parasites were sampled and preserved in 70% ethanol. Parasite abundance per porpoise per organ (or organs in case of left and right lungs) was estimated and either classified in two categories (light, 1–100 parasites; heavy, > 100 parasites) infection or four categories (1–10 parasites; 11–100 parasites; 101–1000 parasites; > 1000 parasites). Nematode length and width were measured. Pulmonary nematodes were specified according to length and host organ infected based on previous research by Gibson and others [23]: nematodes < 30 mm, *Stenurus minor*; 30–70 mm, mixed *Torynurus convolutus* and *Halocercus* sp.; > 100 mm, *Pseudalius inflexus*.

To assess the role of parasitic infections in the lungs as a cause of pneumonia, the presence and abundance of parasites in the lungs were compared between animals with and without pneumonia as a probable cause for stranding, per parasite species and per age category, using the Fisher test (two-sided). A *p* < 0.05 was considered as a significant difference in prevalence and intensity of infection.

### Virology

As morbillivirus infections have been identified as a cause of deaths among harbour porpoises [24] lung and spleen samples of all animals were tested by reverse transcriptase polymerase chain reaction (RT-PCR) for the presence of morbilliviral RNA. Total nucleic acids were isolated from 300 µL of a 10% organ homogenate using the High Pure Viral Nucleic Acid Kit (Roche diagnostic GmbH, Mannheim, Germany), following the protocol provided by the manufacturer. After first strand synthesis, morbillivirus-specific primers P1: 5′ATGTTTATGATCACAGCGGT3′ and P2: 5′ATTGGGTTGCACCACTTGTC3′ were used for PCR. PCR reactions were checked on 2% agarose gels.

### Grey seal attack bite marks

Photographs of suspect lesions of the integument were evaluated according to criteria set by Leopold et al. [25] by one of the co-authors of that article (Begeman).

### Selection of significant lesions and diagnoses

Significant lesions were those lesions considered responsible for stranding by themselves or together with other significant lesions in the same animal. Selection of significant lesions was based on combined analysis of clinical observations and pathological results. A significant diagnosis was defined as a diagnosis based upon the observation of a significant lesion. Significant diagnoses or lesions acquired whilst in rehabilitation, for example an aspiration pneumonia due to tube feeding, were ignored in this manuscript. Incidental diagnoses were those diagnoses based upon the observation of lesions, which were considered too minor to have contributed to stranding. These diagnoses are not further discussed here, but are available in Additional file 1: Table S1.

## Results

### Harbour porpoises rescued and autopsied

The total number of animals admitted for rehabilitation between 2003 and 2016 was 131, of which 61 (47%) were autopsied following death or euthanasia. Forty-three animals were autopsied fresh after having been put on ice immediately after death. They were autopsied within 24 h of death (36 animals) or between 24 and 72 h after death (7 animals). The remaining 18 animals were frozen immediately after death (−20 °C) and autopsied at a later date. Animals autopsied originated from the North Sea coasts of Germany (7), Belgium (2) and the Netherlands (52). Juveniles were the main category of both admitted (106/131; 81%) and autopsied (43/61; 70%) animals, while males and females were more or less equally represented, except in neonates, where only males were presented for rehabilitation (Table 1). Numbers of animals varied between 3 to 10 admitted and 2 to 5 autopsied annually, except for 2006, 2011, and 2012, which had exceptionally high numbers of admissions (18, 15 and 15 respectively) and parallel high numbers of autopsies (12, 6 and 7 respectively). There were more admissions in winter (*n* = 66) than in spring, summer or autumn (*n* = 26, 17, 22 respectively; Additional file 2).

**Table 1:** **Sex and age category of admitted and autopsied harbour porpoises between 2003 and 2016**

Age class	No. admitted (no. M/no. F)	No. autopsied (no. M/no. F)
Neonate	5 (5/0)	4 (4/0)
Juvenile	106 (53/53)	43 (21/22)
Adult	20 (8/12)	14 (4/10)
Total	131 (66/65)	61 (30/31)

### Overview of significant diagnoses

**Figure 1:**
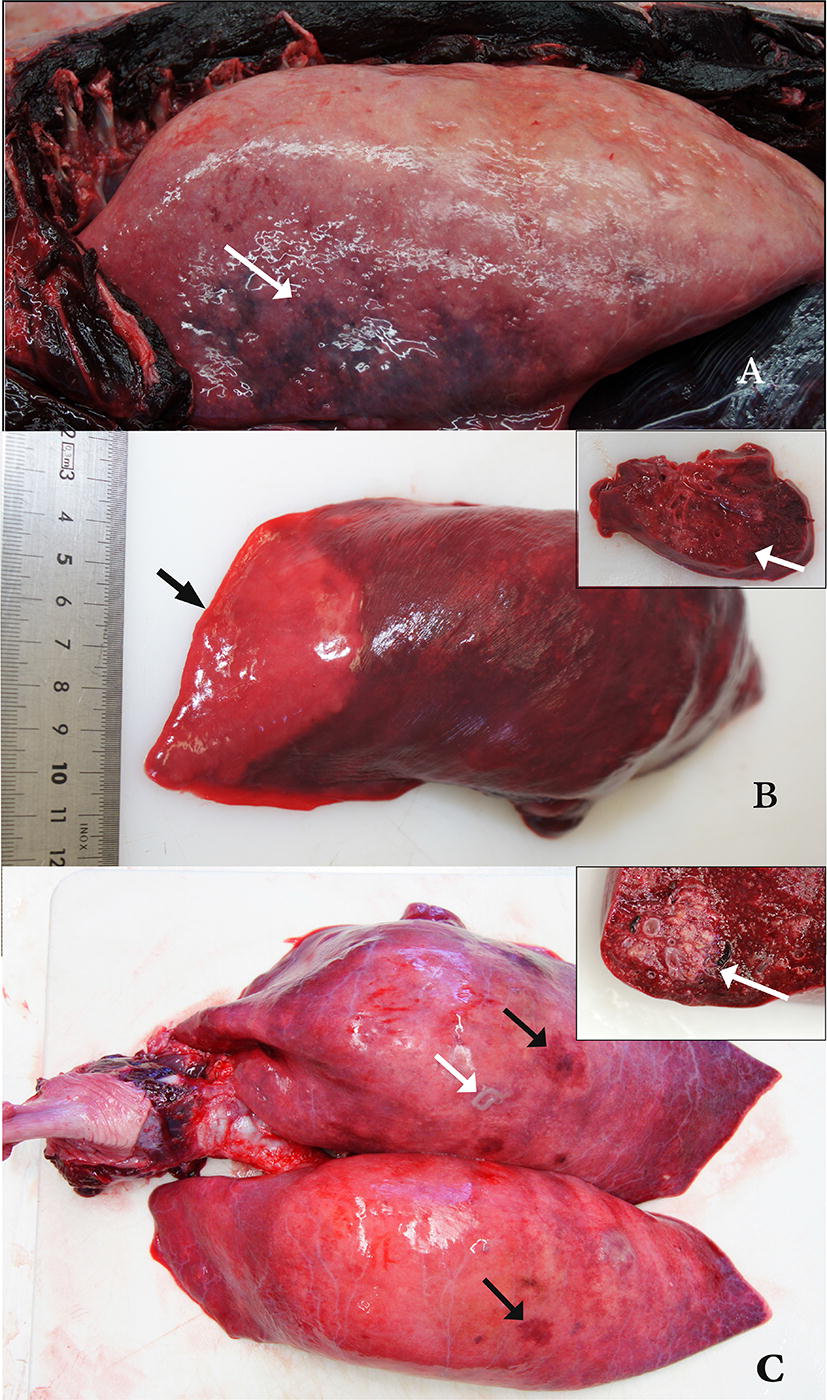
**Macroscopic aspects of pneumonias of varied aetiology in harbour porpoises.** **A** Bacterial pneumonia, a focal purple coloured lesion is present in the ventro-cranial part of the left lung. The white arrow points to the lesion. **B** Fungal pneumonia, a yellow sharply demarcated lesion is present at the caudal tip of the right lung. Insert shows lesion visible at cut surface. Arrows point to lesions. **C** Parasitic pneumonia, multiple nodules of less than 1cm diameter, some associated with a hyperaemic region surrounding or adjacent to the nodule are visible at the surface. Black arrows point to nodules, white arrow points to subpleural a scar caused by a calcified nematode. Insert shows lesion at cut surface, which is a poorly demarcated firm yellow nodule. Extensive gross and histologic description available in supplementary material.

**Figure 2:**
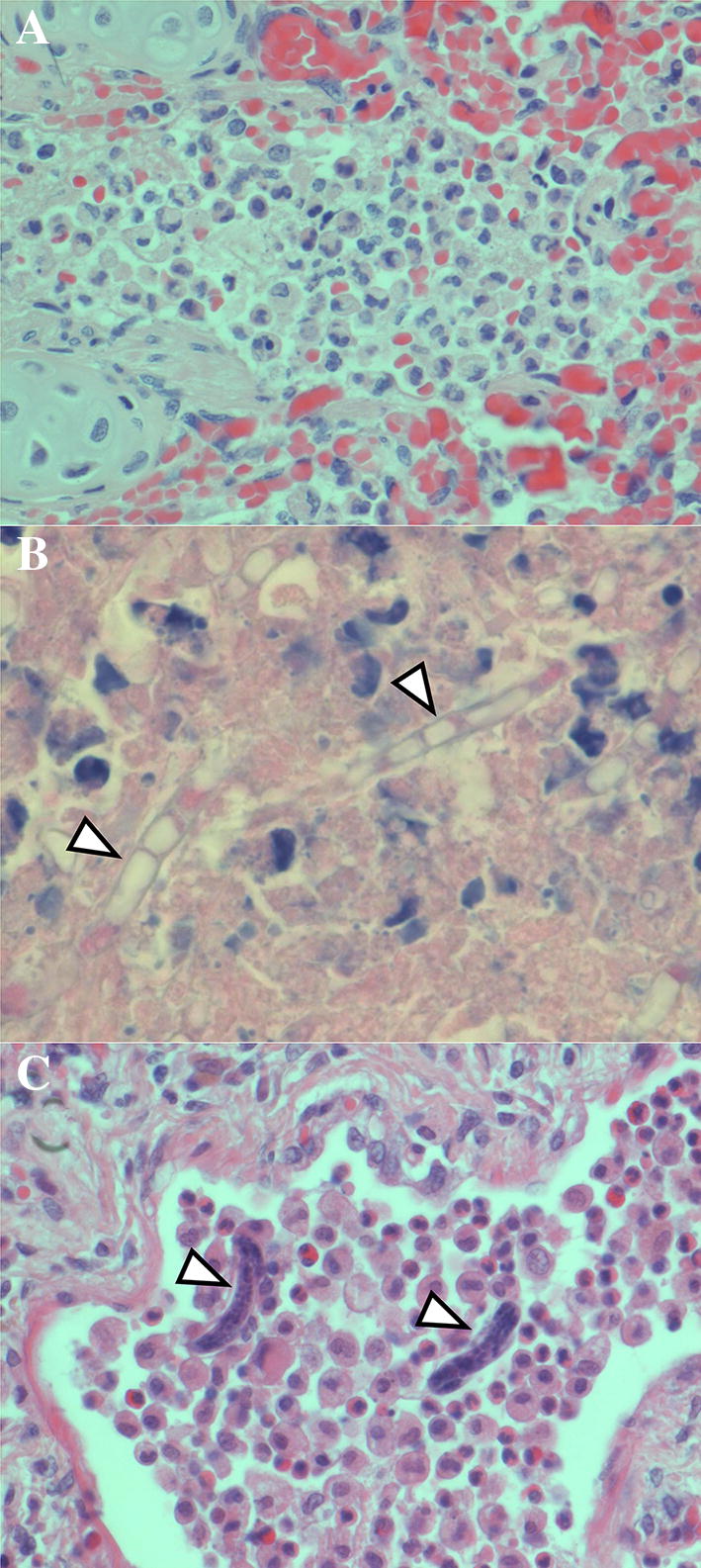
**Distinct histopathological features of pneumonia from different causes.** **A** Bacterial pneumonia in porpoise PP140917. Neutrophils and fibrin fill an alveolar lumen. **B** Fungal pneumonia in porpoise PP121130. Fungal hyphae (arrowheads) with internal segments are present in cellular debris at the edge of a pulmonary abscess. **C** Parasitic pneumonia in porpoise PP040324. Nematode larvae (probably Stenurus minor) (arrowheads), macrophages, and eosinophils fill an alveolar lumen. Haematoxylin and eosin. Original magnifications: 40 X objective (**A**, **C**); 100 X objective (**B**).

Pneumonia was the only significant diagnosis in 13/61 (21%) animals and one of multiple significant diagnoses in 21/61 (34%) animals (Table 2 and Additional file 1: Table S2). The cause of pneumonia was determined in 30/34 (88%) animals: 13 due to parasitic infection combined with bacterial or fungal infection; 8 due to parasitic infection alone; 6 due to bacterial infection alone, 4 due to fungal infection alone, and 1 due to aspiration of gastric content. In most animals with pneumonia as a significant diagnosis, gross lesions were evident (Figure 1). The typical character and distribution of the gross lesions differed among pneumonias of parasitic, bacterial, and fungal aetiology, although there was some overlap. Histologically, the differences were more distinct (Figure 2, description in Additional file 1).

**Table 2:** **Organs affected, morphological diagnosis, and aetiology of significant diagnoses observed in 61 harbour porpoises**

Organ	No. of animals with severe lesion in specified organ											
	Total (single/multiple^a^)	Inflammatory lesion from										
		Nematodes	Nematodes plus bacteria	Nematodes plus fungi	Bacteria	Bacteria plus fungi	Fungi	Viruses	Viruses plus bacteria	Unknown micro-organisms	Unknown cause	Non-inflammatory lesion
Lung	35 (13/22)	9	7	3	5		4^b^			1	5	1
Liver	7 (1/6)				1						4	2
Brain	7 (0/7)					1	2^c^	1			3	
Integument	7 (1/6)				4				1		1	1
Kidney	4 (1/3)											4
Ear	3 (0/3)					2					1	
Muscle	3 (0/3)				1							2
Heart	2 (0/2)						1^d^				1	
Pancreas	2 (1/1)										1	1
Skeleton	2 (0/2)				2							
Oesophagus	1 (1/0)											
Eye	1 (0/1)										1	
Pharynx	1 (0/1)						1^d^					
Stomach	1 (0/1)	1										
Vasculature	1 (0/1)											

^a^Single, number of animals with severe lesion diagnosed only in specified organ. Multiple, number of animals with severe lesion diagnosed also in one or more other organs.

^b^In three of these four animals, fungal infection spread from the lungs to other organs.

^c^In one of these two animals, fungal infection spread from the lungs.

^d^In this animal, fungal infection spread from the lungs.

Five of 61 (8%) animals had only one significant diagnosis based upon a single organ other than the lung (Table 2). Diagnoses were: pancreatic duct hyperplasia, bite wounds by predators in the integument and bones, ulcerative oesophagitis, hepatic necrosis and lipidosis, and protein-losing nephropathy (Additional file 1).

Twenty-nine of 61 animals (48%) had significant diagnoses in multiple organs (Table 2 and Additional file 1: Table S2). Besides the lungs (pneumonia) (*n* = 21; see above), the main organs affected in animals with multi-organ disease (*n* = 7 per affected organ) were liver (hepatitis or hepatic lipidosis), brain (encephalitis or encephalomyelitis), and integument (dermatitis or bite wounds). In 7/29 (24%) of these animals with significant diagnoses in multiple organs, a single aetiology was identified as the cause of the multi-organ disease: fungal infection in 3 animals, with spread from lung or middle ear to brain or pharynx; bacterial infection in 1 animal, with sepsis affecting lungs, muscles and connective tissue; parasitic infection in 1 animal, affecting both lungs and pulmonary blood vessels; bite wounds in 1 animal, affecting both integument and skeleton; and a metabolic disorder in 1 animal, affecting both liver and kidney.

### Specific aetiology of infectious diseases

#### Fungal infections

**Figure 3:**
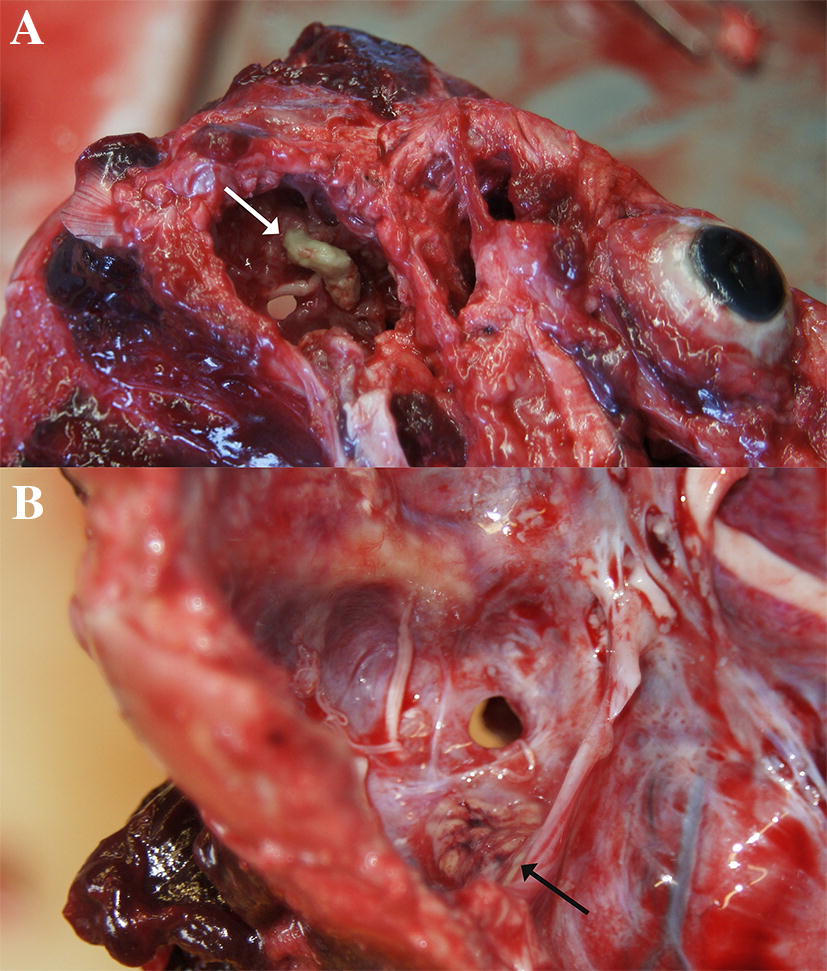
**Fungal infection of the middle ear which extends into the cranial cavity.** **A** View upon the ventral aspect of the skull with the bulla tympanica removed. Green pasty substance is visible (white arrow). **B** View into the cranial cavity adjacent to the inner ear. The inflammation can be seen to extend from the inner ear to the meninges (black arrow).

In all nine animals with significant diagnoses from fungal infections, the aetiology was *Aspergillus* sp. In 6/9 (66%) animals, the fungus was identified as *Aspergillus fumigatus* by culture, while in 3/9 (33%) animals, culture was negative and the fungus was identified as *Aspergillus* sp. by histology, based on characteristic morphology. In 7/9 (78%) animals, the lungs were infected; in 3 animals, aspergillosis was also diagnosed in an additional organ: heart, brain, or pharynx (Table 2). In 2/9 (22%) animals, aspergillosis was diagnosed in the middle ear and had spread to the brain (Figure 3).

#### Viral infections

Two significant diagnoses of viral aetiology were made, one in the brain and the other in the integument. Lung and spleen samples of all animals (*n* = 61) tested negative for the presence of morbilliviral RNA by RT-PCR.

The case with a viral infection of the brain suffered from lymphocytic encephalitis with neuronal necrosis and intranuclear inclusion bodies. It was diagnosed as *Phocoena phocoena* herpesvirus type 2, based on a combination of PCR, virus culture, histology, and electron microscopy [26].

**Figure 4:**
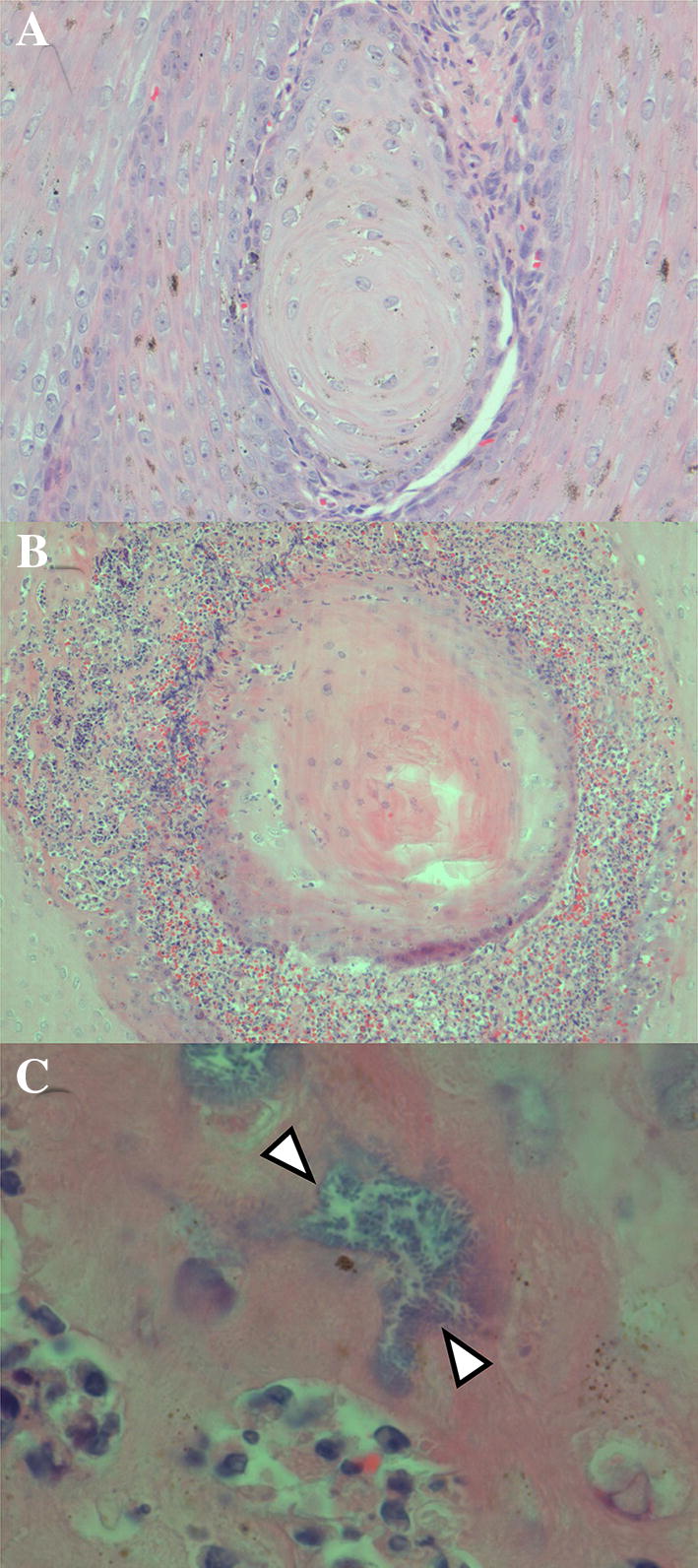
**Histopathological features of the pyogranulomatous dermatitis in porpoise PP121130.** **A** Keratin pearl, consisting of concentric rings of squamous cells with progressive keratinization towards the centre, within the epidermal layer. **B** There is infiltration of many neutrophils and macrophages at the border between keratin pearl and surrounding epidermis. **C** There is an aggregate of bacteria (in between arrowheads) among the infiltrating inflammatory cells. Haematoxylin and eosin. Original magnifications: 20 X objective (**A**); 10 X objective (**B**); 100 X objective (**C**).

The case with a viral infection of the integument suffered from multifocal pyogranulomatous dermatitis (Figure 4). It was suspected to have been caused by a papillomavirus infection based on characteristic histological changes, including epidermal hyperplasia, keratin pearls, invagination of the epidermis and associated increase in vascularization of the subjacent dermis. Bacterial infection and associated inflammation were also present, and were considered to be secondary to the viral infection.

#### Bacterial infections

In 12 animals, bacterial infections lead to significant diagnoses (Table 3). These bacterial infections were considered to be the cause of the observed lesions because infiltration of many neutrophils, with or without macrophages and syncytia, were seen on histology with or without bacteria visible, and bacteria were cultured from samples of the lesions. The diagnoses associated with these bacterial infections were pneumonia in nine animals, sepsis in two animals, dermatitis in two animals, and otitis combined with panencephalitis in one animal. A single bacterial species was responsible for infection in eight animals, while multiple bacterial species were responsible in four animals. *Streptococcus* sp. and *Enterococcus faecalis* were each isolated in three animals, *Escherichia coli*, *Actinobacillus delphinicola*, *Shewanella putrefaciens*, and *Brucella* sp. were each isolated in two animals, and *Salmonella* sp., *Pseudomonas aeruginosa*, and *Clostridium perfringens* were each isolated in only one animal. The *Salmonella* sp. was a monophasic group B *Salmonella* thought to be adapted to, and specific for, the harbour porpoise [27, 28].

**Table 3:** **Significant diagnoses from bacterial infections in respiratory, lymphoid, central nervous, and integumentary systems**

Erasmus code number^a^	Bacterium	Lesion	Observation which links bacterium to lesion
PP041215	*Aeromonas sp.*	Pneumonia, necrotizing, suppurative, locally extensive, acute marked	Aggregates of bacteria with neutrophils. *Aeromonas* sp. cultured from lung
PP110329	*Actinobacillus delphinicola*	Bronchopneumonia, multifocal, acute, marked	Alveoli filled with neutrophils. Part of these neutrophils are degenerate and apparently transformed into globules of dark blue chromatin (as seen with some bacterial infections: nuclear streaming). *Actinobacillus delphinicola* cultured from lung and lung draining lymph node
PP070221	*Actinobacillus delphinicola* *Brucella* sp.	Bronchopneumonia, multifocal, suppurative, acute, moderate	Bacteria observed with fibrin, macrophages, and neutrophils in lung lesions. *Actinobacillus delphinicola* cultured from lung draining lymph node, *Brucella sp.* cultured form lung
PP110711	*Brucella ceti*	1. Pneumonia, pyogranulomatous, locally extensive, chronic, marked	Marked inflammatory reaction with many neutrophils and macrophages. TBLN sample yielded culture of *Brucella ceti*
		2. Lymphadenitis multifocal subacute to chronic marked	Subcapsular infiltration with neutrophils (TBLN), increase of lymphocytes in medulla and vacuolated macrophages in cortex (PSLN) *Brucella ceti* cultured from both lymph nodes
PP120906.3	*Enterococcus faecalis*	Interstitial pneumonia, suppurative, histiocytic, locally extensive, chronic, moderate	Inflammatory reaction typical for bacterial infection. *Enterococcus faecalis* cultured form lung sample
PP030405	*Escherichia coli; Pseudomonas aeruginosa; Streptococcus sp.*	Pneumonia, pyogranulomatous, multifocal, chronic, marked	Histologic association of bacteria with lesion. *Escherichia coli; Pseudomonas aeruginosa; Streptococcus sp.* cultured from lung sample
PP061122.2	*Escherichia coli*	1. Bronchopneumonia, suppurative, multifocal, subacute to chronic, marked 2. Myositis, suppurative, multifocal, acute, moderate 3. Fasciitis, suppurative, focal, acute, moderate	Histologic association of bacteria with lesion. *Escherichia coli* cultured from sample
PP121031	*Salmonella sp.* (host adapted group B *Salmonella*)	Bronchopneumonia, pyogranulomatous, necrotizing, multifocal, chronic, moderate	Histologic association of bacteria with lesion. *Salmonella sp.* cultured from sample
PP120906.2	*Streptococcus dysgalactiae*	1. Interstitial pneumonia, suppurative, diffuse, acute, moderate 2. Hepatic abscesses, multiple, marked 3. Arthritis, suppurative, diffuse, acute, marked 4. Sepsis	Inflammatory reaction typical of bacterial infection (suppurative interstitial pneumonia, multiple marked hepatic abscesses, observation of strings of bacteria in joint capsule with associated inflammatory relation) *Streptococus dysgalactiae* cultured from ear abscess, liver abscess, lung, liver, spleen, kidney, trachea-bronchial lymph node, pre-scapular lymph node, adrenal and uterus
PP121130	*Shewanella putrefaciens* *Enterococcus faecalis* Gram negative non-fermenter	Dermatitis, pyogranulomatous, multifocal, chronic, marked, with epidermal hyperplasia, keratin pearls, and bacterial infection	Many aggregates of small coccobacilli mixed with many neutrophils and macrophages observed in the epidermis
PP111219	*Escherichia coli Stenotrophomonas maltophilia*	Bronchopneumonia, haemorrhagic, suppurative, diffuse, acute, marked, associated with bacterial infection	Histologic association of bacteria with lesion. *Salmonella sp.* cultured from sample
PP050502	*Shewanella putrefaciens* *Enterococcus faecalis* *Streptococcus* sp.	Dermatitis, multifocal, suppurative, superficial, acute, moderate	Histologic association of bacteria with lesion and bacteria cultured from sample
PP060524	*Clostridium perfringens*	1. Cerebellum: panencephalitis, pyogranulomatous, haemorrhagic, necrotizing, locally extensive, marked associated with fungal hyphae (*Aspergillus* sp.) and mixed bacterial infection 2. Otitis media purulent subacute to chronic diffuse marked	Bacteria observed centrally in pyogranulomas in meninges and cultured from middle ear and brain samples

^a^Erasmus code numbers are made of two letters as acronym for genus and species (here *Phocoena phocoena*) and six numbers indicating year, month and day of necropsy. The suffix T indicates the animal was treated with fenbendazole (an antiparasiticum) during rehabilitation.

#### Parasitic infections

##### Respiratory tract

The pulmonary nematodes *S. minor*, *T. convolutus*, *Halocercus* sp., and *P. inflexus* were found in juveniles both with and without a significant diagnosis of pneumonia (Table 4 and Additional file 1: Table S3). Prevalences and intensities of both *T. convulutus*/*Halocercus* sp. infection and *P. inflexus* infection were significantly higher in juveniles with significant diagnoses of pneumonia than in juveniles without severe pneumonia (*p* < 0.05, Fisher test two sided), but prevalence and intensity of *S. minor* did not differ significantly between the two groups (*p* > 0.05, Fisher test two sided). No significant differences in prevalence and intensity of any of the pulmonary nematode species were found between adults with and without severe pneumonia (Additional file 1: Tables S4 and S5).

**Table 4:** **Presence and burden of nematode infections in juvenile harbour porpoises with and without pneumonias of different aetiologies**

Lung pathology and aetiology	No. of harbour porpoises infected (no. with light infection/no. with heavy infection)		
	*Stenurus minor*	*Torynurus convolutus*/*Halocercus* sp.	*Pseudalius inflexus*
No severe pneumonia (*n* = 20)	3 (3/0)	5 (4/1)	1 (1/0)
Severe pneumonia (*n* = 21)	2 (2/0)	14 (5/9)	11 (5/6)
Parasitic (*n* = 6)	1	4 (2/2)	4 (1/3)
Bacterial (*n* = 5)	1 (1/0)	2 (2/0)	2 (2/0)
Fungal (*n* = 2)	0	1 (1/0)	1 (1/0)

Light = 1–100 nematodes (both lungs).

Heavy ≥ 100 nematodes (both lungs).

Pulmonary nematodes were not detected in neonates. The estimated age of the youngest juveniles in which pulmonary nematodes were detected was 9 months (*S. minor*), 6.5 months (*T. convolutus*/*Halocercus sp.*), and 5 months (*P. inflexus*). *Pseudalius inflexus* had a significantly higher prevalence and intensity in adults than in juveniles (above 6 months of age) (*p* < 0.05, Fisher test two sided); Infections with *T. convolutus/Halocercus* sp. and *S. minor* did not differ significantly in prevalence and intensity of infection between juveniles (13/42, 31% infected) and adults (10/12, 83% infected).

##### Pulmonary vasculature

The prevalence and intensity of *P. inflexus* infection in pulmonary blood vessels was significantly higher in adults (10/14, 71% infected) than in juveniles (19/41, 46% infected) (*p* < 0.05, Fisher test two sided; Additional file 1: Table S6). Histologic lesions of the pulmonary vasculature were observed only in animals with an associated *P. inflexus* infection (Additional file 1). No gross lesions of the pulmonary vasculature were observed. The youngest animal with a *P. inflexus* infection of the pulmonary vasculature was estimated to be 5 months of age.

##### Digestive tract

Parasitic infections in the organs of the digestive tract were not associated with significant diagnoses. *Campula oblonga* infection of the liver and *Anisakis simplex* infection of the forestomach occurred significantly more often in adults than in juveniles (*p* < 0.05, Fisher test two sided). The prevalence of *C. oblonga* infection of the pancreas, *Pholeter gastrophilus* infection of the pyloric stomach and *Diphyllobothrium stemmacephalum* of the intestine did not differ significantly between juveniles and adults (*p* > 0.05, Fisher test two sided). Prevalences of parasitic infections of organs of the digestive tract are available in Additional file 1: Tables S7 and S8. The estimated age of the youngest animals with parasitic infections of the digestive tract were: 9 months for *C. oblonga* infection of the liver, adult (of unknown age) for *C. oblonga* infection of the pancreas, 7 months for *A. simplex* infection of the fore stomach, 9 months for *P. gastrophilus* infection of the fundic stomach and 11 months for *D. stemmacephalum* infection of the intestine.

### Aetiology of non-infectious diseases

#### Separation from mother animal

The probable cause of stranding in 10/61 (16%) animals was separation from the mother in mother-dependent animals. Three of these were neonates, and seven were emaciated juveniles of less than 10 months of age, at which time they were still mother-dependent [29]. The diagnosis was based on severe emaciation (only observed in juveniles, not in neonates), together with absence of other lesions that could explain stranding. Emaciation was characterized by atrophy of the epaxial and cervical muscles, absence of internal fat (e.g. around heart and lungs), and a thin blubber layer (less than 15 mm average measured at the circumference cranial to the dorsal fin). Emaciation was externally visible as the dorsolateral surface of the body at the level of the dorsal fin being concave rather than convex, and the presence of a dorsal indentation between head and thorax, rather than a flush transition. In six out of seven emaciated animals, the blubber layer was thinner than 15 mm. Normal blubber thickness is 18 to 20 mm on the thorax [30].

### Physical trauma from putative grey seal attacks

**Figure 5:**
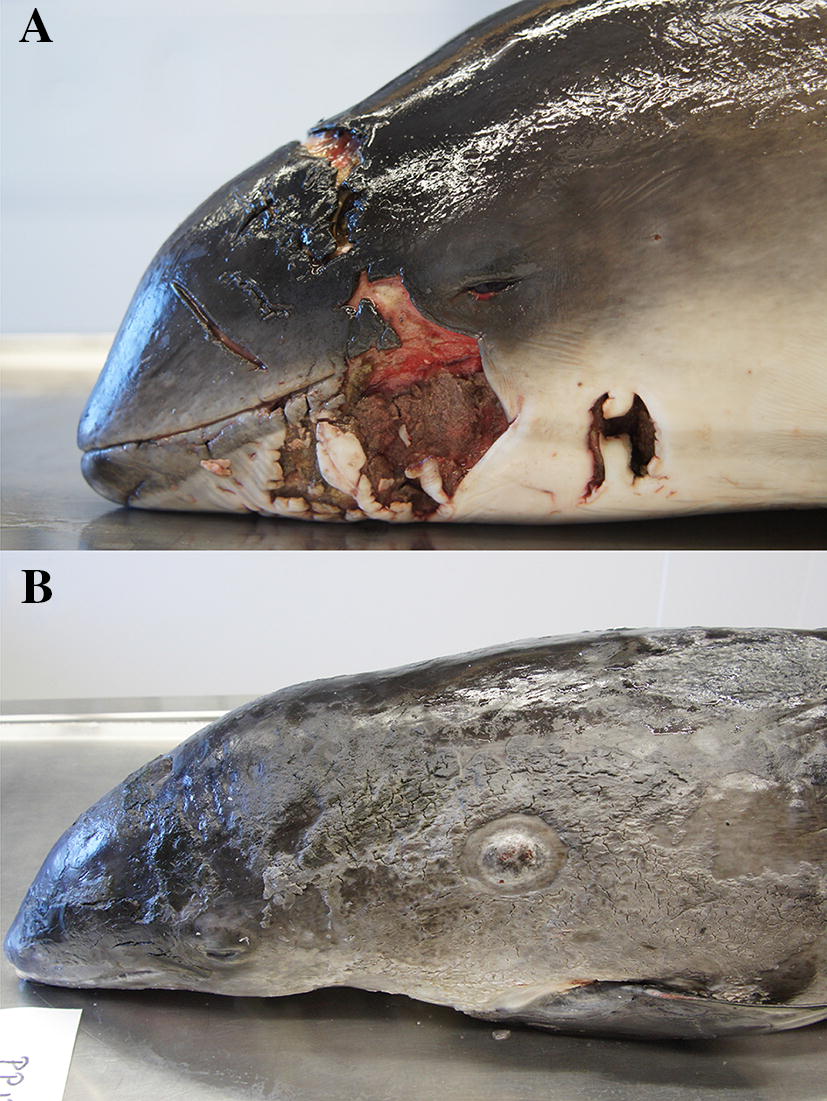
**Lesions of the integument.** **A** Traumatic lesions caused by a grey seal attack on a live(!) stranded harbour porpoise. **B** Generalized inflammatory lesions of the integument caused by a mixed viral and (secondary) bacterial infection.

In three juveniles and one adult animal, lesions attributable to grey seal attack were observed. These animals stranded in 2011, 2015 and 2016. Lesions occurred on the tail stocks of all animals, on the pectoral flipper of one animal, and on the head of another. The head lesion was so severe that the animal had to be euthanized (Figure 5). Trauma from grey seals was considered to be a significant diagnosis in two animals and an incidental diagnosis in the remaining two.

### Organ weights and sizes

We found the following associations between the 5% highest or lowest organ weights and significant diagnoses: high brain weight with encephalitis; high lung weight with pulmonary congestion; low liver weight with cholangitis; and high adrenal weight with hyper- or hypoplasia of the adrenal gland (Additional file 3).

### Comparison of clinical and pathological observations

Clinical observations were made for 48/61 animals during rescue and rehabilitation (Additional file 1: Table S9). In some cases, significant diagnoses after death correlated well with clinical signs before death. Animals with a significant diagnosis of only pneumonia (*n* = 7) showed the following respiratory signs: bradypnea (2/7), tachypnoea (2/7), exaggerated breathing movements (1/7) or rhonchi in the bronchi during auscultation (1/7). Animals with significant diagnoses of fungal otitis media and encephalitis (*n* = 2) showed both nervous signs and non-specific clinical signs: uncoordinated swimming behaviour (2/2), vertical nystagmus and delayed pupillary reflex unilaterally (1/2), increased cardiac rate (1/2) and electrolyte imbalance with decreasing total protein in the serum despite good food intake (1/2). Animals with significant diagnoses of both encephalitis and a pneumonia (*n* = 5) showed both respiratory and nervous signs: dyspnoea with forced laboured breathing (3/5), tachypnoea (2/5) and lifting of the entire head out of the water for inspiration (2/5).

In few animals, there were notable discrepancies between significant pathological diagnoses and clinical signs: three animals with nervous signs had no or only mild brain lesions at autopsy, and two animals with clinical signs of kidney failure had only mild kidney lesions at autopsy (Table 5). For most animals with significant diagnoses in multiple organs (*n* = 23), as well as animals with no significant diagnosis (*n* = 4), it was not possible to compare significant pathological diagnoses with clinical signs.

**Table 5:** **Animals with discrepancies between clinical signs and pathological observations**

Animal	Clinical signs	Pathology observations
1	Multiple epileptic seizures with loss of control	No lesions observed
2	Kidney failure, marked increase in urea, creatinine and sodium values with loss of appetite and vomiting	Nephritis, suppurative, focal, acute, mild Renal medullary calcification, multifocal, mild
3	CNS: Body tremor, forceful difficult expiration Digestive or CNS: Cramps gastric stasis Respiratory or CNS: increased breathing frequency	CNS nad. Cornea and brain herpesvirus PCR positive Digestive: nad Respiratory: pulmonary oedema (acute agony related)
4	CNS symptoms: hypothermia, disorientated swimming against the wall, laboured breathing with vertical rises above the water to inspire	Cerebrum: polioencephalitis, multifocal, mild
5	Kidney failure: marked increase urea, creatinine, sodium, vomiting	Kidney: urolithiasis, mild, some protein granules in the collecting ducts

Nad: no abnormalities detected.

## Discussion

In the present paper we have investigated diseases, of live stranded harbour porpoise, that were severe enough to have contributed to stranding. In comparison with previous surveys [13–16], we observed a higher prevalence of fungal diseases, a higher prevalence of significant lesions in integument, brain, kidney and liver, and a higher prevalence of animals which had significant lesions in multiple organs (Table 6).

**Table 6:** **Comparison of frequency, location and aetiology of causes of death or stranding in harbour porpoises from the North Sea of five autopsy overviews**

Publication	Location of lesion, or reason responsible for stranding or death								
	Lungs	Starvation	Brain	Liver	Integument	Kidney	Sepsis	Unknown	Other
British waters 1979–1991^a^ [13] (*n* = 31) 21 neonates, 30 juveniles, 49 adults									
Total prevalence	*45*	*10*				*3*	*6*	*10*	*32*
Parasites	23								
Bacteria	13						6		
Fungi	3								
Viruses									
Non inflammatory	6					3			
German North and Baltic seas 1991–1996^a, b^ [16] (*n* = 66) 5 foetuses, 35 < 0.5 years, 0.5 < 41 < 4 years, 4 years < 20									
Total prevalence	*46*	*≤* *7*	*?*	*?*	*?*	*?*	*?*	*24*	*?*
Parasites plus bacteria	Mainly								
Fungi	2								
Belgium and Northern France 1990–2000^a^ [14] 11 neonates, 57 juveniles, 32 adults									
Total prevalence	*56*	*9*	*4*				*2*	*?*	
Parasites	26								
Bacteria			2				2		
Parasites plus bacteria	30								
Unknown			2						
England and Wales 1990–1995^a^ [15] (*n* = 104) ages not specified									
Total prevalence	*28*	*20*	*2*	*1*			*7*	*23*	*19*
Parasites	8								
Bacteria	6						6		
Parasites plus bacteria	8								
Fungi	2								
Viruses							1		
Non inflammatory									
Unknown	5		2	1					
Dutch and adjacent coasts 2003–2016 (*n* = 61) 7 neonates, 70 juveniles, 23 adults									
Total prevalence	*58*	*16*	*11*	*11*	*13*	*7*	*5*	*7*	*20*
Parasites	15								
Bacteria	10			2	7		5		
Parasites plus bacteria	11								
Fungi	7		3						
Fungi plus parasites	5								
Fungi plus bacteria	11		2						
Viruses			2						
Viruses plus bacteria					2				
Non inflammatory	2			3	5 (trauma)	7			
Unknown	8		5	7					

Between brackets are number of animals autopsied. All other numbers are percentages. Italics numbers are total percentages.

Prevalence for causes of stranding for the publication referring to the Dutch coast, prevalence for cause of death for the four other publications.

?, could not be deduced from publication.

^a^Bycaught animals, decomposed animals and animals dead due to suspected by-catch related trauma excluded.

^b^Results reported for mixed bycaught and stranded animals, only results reported for which it was clear they related to stranded animals.

Our study noted a high prevalence of fungal infections, which were mostly caused by *A. fumigatus,* and used the lungs or middle ears as portal of entry. The prevalence of fungal diseases was higher in our study (15%) than in previous surveys (2 to 3%) [13–16] and in a study of pulmonary pathology of stranded harbour porpoises (5%) [31]. All 9 infections noted by us were caused by *Aspergillus* sp. derived from the typical histologic appearance of the fungal hyphae. In six cases we could further identify the species to *Aspergillus fumigatus* by successful culture. Comparison to previous research is difficult as successful cultures are only reported sparsely. Siebert et al. noted one mycotic infection caused by *Rhizopus sp.* and Jepson et al. were able to culture *Aspergillus terreus* in one case. *Aspergillus fumigatus* has been identified in a middle ear infection and a brain infection [32, 33] and *Aspergillus terreus* has been identified in a middle ear infection [34]. Most infections we observed (7/9) were fungal pneumonias which spread via the blood to brain, heart and pharynx in three separate cases, analogous to the dissemination of invasive aspergillosis in humans [35]. The two other cases we observed were fungal middle ear infections, which spread per continuitatum to the brain. In these two cases consequences were clearly visible in the live animals which showed neurological signs such as erratic swimming behaviour, vertical nystagmus and unnatural body posture. Our and previously reported middle ear infections in harbour porpoises [33, 34] were infections without evidence of pulmonary involvement. We speculate that infectious *Aspergillus* conidia in inhaled air entered the middle ear via the Eustachian tube. Compared to terrestrial mammals, Eustachian tubes in cetaceans are broader and firmer. This anatomical adaptation is thought to guarantee airflow and thus prevent barotrauma when diving [36], but also might act as an efficient portal of entry for *Aspergillus* infections.

We speculate that the higher prevalence of aspergillosis we observed in harbour porpoises is caused by impaired immunity rather than increased exposure to infectious *Aspergillus* conidia or more sensitive diagnosis of aspergillosis. Impaired immunity due to host damage is a requirement for the development of invasive aspergillosis. In the immunocompetent host, defence mechanisms show a striking redundancy [37]. Although prevalence of *Aspergillus* infections is related to infection pressure in birds [38], there is no reason to assume that the higher prevalence of aspergillosis we observed in harbour porpoises is due to increased infection pressure from *Aspergillus* conidia for harbour porpoises in recent decades. An increased amount of decaying plant material and composting facilities around coastal areas would provide justification for such a suspicion. To the best of our knowledge these changes have not occurred in countries around the North Sea. It is also unlikely that the higher prevalence of aspergillosis is due to more sensitive diagnosis of aspergillosis. Gross lesions were clearly visible and histologic confirmation was straightforward (Figures 2, 3).

Impaired immunity, responsible for the increased prevalence of aspergillosis, could be caused by anthropogenic pollutants, viral infections or malnutrition. The influence of anthropogenic pollutants on the immune system of harbour porpoises is a possible cause for impaired immunity. Several investigations have found a positive correlation between high levels of heavy metals and polychlorinated biphenyls (PCBs) in harbour porpoise tissues and the prevalence of infectious diseases [5, 6]. Hall et al. quantified the increase in risk of mortality due to infectious disease by the concentration of PCB congeners in the blubber layer of harbour porpoises. They stated that a concentration above 25 mg/kg lipid put the animal at an increased risk of mortality [39]. Individuals from our study were well above this 25 mg/kg threshold as was observed by Weijs et al. [7]. Dutch coastal waters are the first to receive the heavily polluted waters from the rivers Rhine, Meuse, Waal, and Eems and have higher levels of PCBs than other regional areas [40]. Another possible cause for impaired immunity is virus induced immunosuppression. We did not observe any infections with morbillivirus, which has been documented as a likely immunosuppressant virus in harbour porpoises. We may have overlooked infections with other viruses. Viral infections can easily be overlooked as knowledge about which viral infections occur in marine mammals is far from complete and one therefore does not know what to look for nor does one know where to look. Finally, malnutrition is a well-known cause for impaired immunity [41]. Our data set did not allow the assessment of sub-lethal effects, such as impaired immunity, by malnutrition.

We observed that a parasitic infection was not a prerequisite for a bacterial pneumonia, as speculated previously [14, 16]. Pure bacterial pneumonias occurred independently of prevalence or intensity of parasitic infections. Harbour porpoises with bacterial pneumonias and without pneumonias had the same prevalence and intensity of pulmonary parasitic infections with *S. minor*, *P. inflexus* or *Halocercus* sp. and *T. convolutus.* (Fisher test > 0.05; Additional file 1: Table S5). However, we did find that an increased prevalence and intensity of parasitic infections of the lung with *P. inflexus* and *T. convolutus* and *Halocercus* sp. were associated with fungal and combined bacterial-parasitical pneumonias (Additional file 1: Table S5).

Infestation of airways with a large number of parasites did not necessarily cause clinical or pathological evidence of disease. Some researchers speculated that pulmonary parasites blocked bronchi and thereby caused fatal respiratory problems [13, 16]. However, we observed harbour porpoises with heavy infestation with *P. inflexus* or *Halocercus sp.* and *T. convolutus* (Additional file 1: Tables S3 and S4) without any associated clinical signs or lesions. Our observations concur with the observations by Kirkwood et al. [15] and Clausen et al. [17], who noted that harbour porpoises were able to tolerate large numbers of lungworms.

We found that prevalence and intensity of *P. inflexus* infection increase with age: these values were significantly higher in adults than in juveniles. Because prevalence and intensity of *P. inflexus* infection are correlated with frequency of pneumonia, this means that results on pneumonia and lung parasitism should be presented separately for each age class: neonates, juveniles and adults. The comparison of our study to previous studies with regard to pulmonary parasitic infections was complicated by the fact that previous surveillance studies categorized infections as mild, moderate or marked, without quantifying the actual numbers of parasites in each of these categories [13–16]. We feel that such quantification is important to investigate the impact of pulmonary nematodes on pulmonary health.

We observed a higher prevalence of significant lesions in integument, brain and liver than were indicated in previous studies [13–16] (Table 6). In the integument, we observed significant lesions in 13% of the animals, mostly due to bacterial infections (5/8 animals); our report stands alone in this. Baker et al. reported lesions of the integument (trauma, non-specific and viral) in 40% of the animals in his study but considered these lesions to be non-fatal [13]. Jauniaux et al. reported ulcerative skin lesions in 20% of the animals, with severe lesions in 4%, but did not clarify whether these lesions were considered severe enough to cause stranding or death [14]. Siebert et al. reported suppurative or necrotizing inflammation of the integument in 8% of the animals, but did not clarify whether these lesions were considered significant or incidental, and whether they had occurred in stranded or by-caught animals [16].

In the brain, we observed significant lesions in 11% of the animals. Neither Baker et al. [13] nor Siebert et al. [16] reported brain lesions. Kirkwood et al. [15] observed brain lesions in 2% of the animals and Jauniaux et al. [14] reported brain lesions in 4% of the animals. In previous studies, carcinoma [42], *Toxoplasma* [43, 44], and *A. fumigatus* [32] have been diagnosed as causes of brain lesions. In comparison, we observed *A. fumigatus* or *Aspergillus* sp. infection in half of the brain cases, and no cause in the other half, except for one case of *Phocoena phocoena* herpesvirus type 2 infection [26]. We may have missed some brain lesions by routine histological examination of one location each from cerebrum, cerebellum and brain stem, as nervous signs without supporting pathological diagnoses in the brain were observed in 5% of the animals (Table 5). Therefore, more extensive histological examination of the brain is warranted to increase the detection of brain lesions in harbour porpoises.

In the liver, we observed significant lesions in 11% of the animals. In previous surveillance studies [13–16], only Kirkwood et al. [15] observed lesions in the liver as a cause of death, and that was in only 1% of the animals. Liver lesions in harbour porpoises have been reported by other researchers. Hiemstra et al. [45] noted liver lesions not caused by parasitic infection in 32 stranded harbour porpoises, and Herder et al. [43] described a hepatitis caused by a generalized *Toxoplasma* infection.

A possible explanation for the observed differences in prevalences of significant lesions in liver, brain, kidney, and integument between our study and previous surveillance studies [13–16] may at least in part be due to different approaches to assigning diagnoses. Kirkwood et al. strictly assigned a single cause of death to each single animal [15]. Baker et al. diagnosed 30 lesions as being responsible for death in 28 animals, but did not indicate the diagnoses per animal [13]. Siebert et al. [16] and Jauniaux et al. [14] provided a broad outline of all lesions encountered, but did not indicate clearly which lesions, and in which frequencies, they considered responsible for death. In our study, we assumed that significant diagnoses in multiple organs may have operated together to cause stranding; we recorded significant diagnoses in multiple organs in 48% (29/61) of the animals. Our assumption is in line with that of Wobeser [46], who stated that “while we tend to think about diseases one at a time, wild animals are affected by many different agents, often simultaneously”. In our view, Wobeser’s statement also holds true for causes of stranding in harbour porpoises.

Starvation was the second most frequent cause for stranding or death in all studies (Table 6; [13–16]). One point to note is that almost all animals, with the exception of two adults, in the study by Jauniaux et al. were neonates or juveniles [14] and the oldest juveniles were around weaning age. This suggests that starvation was mainly due to separation of the juvenile or neonate from the mother and subsequent inability to forage adequately. No adults or independent juveniles were found with signs of starvation, with the exception of the two animals mentioned above. It seems that food shortage does not cause direct starvation in independent harbour porpoises. However, sublethal effects of malnutrition, for example on immunity or fecundity, cannot be assessed by analysis of the data available in this investigation.

Our study on live-stranded harbour porpoises differs from previous surveys, in which dead-stranded harbour porpoises were examined [13–16]. This raises the question of whether it is valid to compare the two. These two samples might differ if a significant number of dead stranded animals died due to diseases which caused death so rapidly, that they would not have had the opportunity to be found stranded alive. A well-known and frequent cause of acute death in harbour porpoises is bycatch [13–16]. Because the vast majority of bycaught animals may be expected to die in the net and strand dead, we excluded known and suspected bycaught animals in the discussion and Table 6.

Another potential cause of acute death in cetaceans is sepsis [47]. However, the percentage of sepsis encountered in our investigation is relatively small (less than 7%) and similar to the percentage encountered in previous surveillance studies on dead stranded animals (Table 6; [13–16]). Therefore, we retained this cause of death in our comparison between live- and dead-stranded animals. A caveat that is valid for all studies on stranded animals, is that the sample of live-stranded harbour porpoises is expected to originate mainly from the part of the population staying close to shore, and it is unknown whether this part of the population is representative for the entire harbour porpoise population.

## Recommendations for future research

Concerning diagnostic evaluations among live stranded harbour porpoises, our main recommendations are to conduct:

- More research on *Aspergillus* sp. infections in harbour porpoises;
- More research on the immune system in animals with aspergillosis, focussing on immune organs (lymph nodes and spleen) and cellular immunity (e.g. T lymphocytes).
- More extensive histopathological analyses of the brain and to better quantify pulmonary parasitic infections as part of the autopsy protocol;
- More uniform reporting of diagnoses, in order to facilitate analysis and comparison of different autopsy surveys of harbour porpoises.

The most concerning finding of our study was an apparent increase in *Aspergillus* sp. infections as a cause of stranding in comparison with similar studies in the past. Future research should investigate whether this increase is consistent over time and across different regions of the North Sea, and to determine the causes of increase, including impaired immunity.

The brain deserves proper attention as in many cases it is the organ, which carries lesions responsible for stranding or death. Present autopsy protocols regularly fail to identify lesions, which cause nervous signs in live animals, or fail to identify the aetiology of brain lesions that are identified. Sampling protocols should be reassessed and possibly larger numbers of samples should be taken routinely for histology and tissue banking. Bacterial culture of brain samples should be done routinely. Modern diagnostic techniques like RT-PCR and deep sequencing should be considered for more sensitive diagnosis of known infectious agents, and discovery of novel infectious agents. The biggest potential for success is with the search for viruses as causes of disease, as virus infections can be more difficult to identify during gross necropsy or histology than bacterial, protozoal or fungal infections.

Extensive autopsy programs are useful for conservation of species and the environment. They may help to recognize causes or changes in causes of morbidity and mortality and relate these to (anthropogenic) environmental stressors. The effects of environmental stressors on prevalence of disease agents may be subtle and difficult to note in harbour porpoises, which often have multiple lesions [48]. In order to discern these effects and allow comparison among geographical regions, long-term autopsy programs will be necessary to both provide adequate detail and present their results in a uniform manner [49, 50]. Program reports should make sufficiently clear at which frequencies diagnoses occur. Pneumonias should be reported separately for different age classes and quantification of intensity of pulmonary parasitic infections should be provided. A clear differentiation should be made between significant and incidental diagnoses.

## Supplementary information

**Additional file 1. Pathology per organ.** Respiratory tract pathology: 1. Pneumonias: i. Parasitic pneumonia gross lesions and histology, ii. Bacterial pneumonias gross lesions and histology. iii. Fungal pneumonias gross lesions and histology. 2. Pathology of the pulmonary vasculature, 3. CNS pathology: a. Fungal infection, histology, b. Viral infection, histology, c. Inflammation of unknown aetiology, histology. 4. Liver pathology: a. Non inflammatory lesions, gross lesions and histology, b. Bacterial infection, gross lesions and histology. Organ sizes and weights (relative to body length) in relation to lesions observed: **Table S1.** Individual animals with their lesions distributed into lesions which contributed to stranding, did not contribute to stranding or were acquired after stranding, with comment interpreting the severity of lesions. **Table S2.** Organs involved and etiological categories involved in significant diagnoses, per stranded harbour porpoise. **Table S3.** Nematode infections in juvenile harbour porpoises with and without severe pneumonia. **Table S4.** Nematode infections in adult harbour porpoises with and without severe pneumonia. **Table S5.** *p* values according to Fisher’s exact test (two-sided) comparing nematode infections in juvenile harbour porpoises with severe pneumonia to those in juveniles without severe pneumonia (*n* = 20). **Table S6.** Comparison of prevalence and abundance of *Pseudalius inflexus *infections in the pulmonary vasculature of juveniles and adult harbour porpoises. **Table S7.** Comparison of prevalence of gastrointestinal parasites in juvenile and adult harbour porpoises. **Table S8.** Comparison of prevalence and abundance of different parasite species in the digestive tracts of juvenile and adult harbour porpoises. **Table S9.** Overview of lesions, diagnosis and most prominent clinical signs.

**Additional file 2.** **Number of annual admissions according to season, age class and gender.**

**Additional file 3.** **Raw data and graphs of organ weights and sizes in relation to body length.**

## Data Availability

All data generated or analysed during this study are included in this published article (and its additional files).
