# Supplementary material for: Clinical, pathological, and laboratory diagnoses of diseases of harbour porpoises (Phocoena phocoena), live stranded on the Dutch and adjacent coasts from 2003 to 2016
Source: Vet Res. 2019 Oct 30;50:88. doi: 10.1186/s13567-019-0706-3 (PMC6822343; doi:10.1186/s13567-019-0706-3)
Supplement: Supplementary file 1 — Additional file 1. Pathology per organ. Respiratory tract pathology: 1. Pneumonias: i. Parasitic pneumonia gross lesions and histology, ii. Bacterial pneumonias gross lesions and histology. iii. Fungal pneumonias gross lesions and histology. 2. Pathology of the pulmonary vasculature, 3. CNS pathology: a. Fungal infection, histology, b. Viral infection, histology, c. Inflammation of unknown aetiology, histology. 4. Liver pathology: a. Non inflammatory lesions, gross lesions and histology, b. Bacterial infection, gross lesions and histology. Organ sizes and weights (relative to body length) in relation to lesions observed: Table S1. Individual animals with their lesions distributed into lesions which contributed to stranding, did not contribute to stranding or were acquired after stranding, with comment interpreting the severity of lesions. Table S2. Organs involved and etiological categories involved in significant diagnoses, per stranded harbour porpoise. Table S3. Nematode infections in juvenile harbour porpoises with and without severe pneumonia. Table S4. Nematode infections in adult harbour porpoises with and without severe pneumonia. Table S5. p values according to Fisher’s exact test (two-sided) comparing nematode infections in juvenile harbour porpoises with severe pneumonia to those in juveniles without severe pneumonia (n = 20). Table S6. Comparison of prevalence and abundance of Pseudalius inflexus infections in the pulmonary vasculature of juveniles and adult harbour porpoises. Table S7. Comparison of prevalence of gastrointestinal parasites in juvenile and adult harbour porpoises. Table S8. Comparison of prevalence and abundance of different parasite species in the digestive tracts of juvenile and adult harbour porpoises. Table S9. Overview of lesions, diagnosis and most prominent clinical signs. [file 13567_2019_706_MOESM1_ESM.docx]

# **Additional file 1 Pathology per organ**

### Respiratory tract pathology

#### Parasitic pneumonia (n = 8):

##### Gross lesions:

Lung lesions associated with adult nematodes (probably *Halocercus invaginatus*) were characterized by the presence of multiple nodules (range: 3 to 20 per animal). Typically, these nodules were between 5 to 10 mm in diameter, yellow, white, or grey, firm to hard, and poorly demarcated. Those nodules at the lung surface protruded a few mm above the surrounding lung tissue. The lung parenchyma around some nodules was discoloured brown to purple (haemorrhage). On section, some nodules had tightly coiled nematodes, pus, or both in the centre.

Lung lesions associated with larval nematodes (probably *Halocercus invaginatus* or *Stenurus minor*) were characterized by a locally extensive area of consolidated, purple lung parenchyma. On section, pus exuded from bronchiolar lumina. In one adult female a thick-walled abscess surrounding a parasite with spikes on the cuticle and a filled coelomic cavity (suspected trematode) was found, a trematode egg was observed in the lungs of a juvenile female.

##### Histology

Lung lesions associated with adult nematodes (probably *Halocercus invaginatus*) (*n* = 7) were centred on multiple distinct aggregates of a few adult nematodes coiled up in the alveoli, bronchioles or bronchi. In two animals, these nematodes were surrounded by aggregates of macrophages and neutrophils, with or without multinucleated giant cells. These were diagnosed as chronic, multifocal, pyogranulomatous pneumonia. In two other animals, abscesses had formed, in which the nematodes were surrounded by an inner layer of fibrous connective tissue and an outer layer of macrophages, neutrophils, eosinophils, and lymphocytes. These were diagnosed as pulmonary abscessation.

Lung lesions associated with larval nematodes (probably *Halocercus invaginatus* or *Stenurus minor*) were characterized by diffuse flooding of alveolar and bronchiolar lumina by many neutrophils and few macrophages, mixed with larval nematodes. This was diagnosed as acute, locally extensive, suppurative pneumonia.

#### Bacterial pneumonias (n = 8 with parasitic pneumonia, n = 6 without parasitic pneumonia):

##### Gross lesions:

Lung lesions associated with bacterial infections varied in gross appearance. In 4 animals, no gross lesions suggestive of bacterial infection were observed. Where bacterium-associated lesions were seen, they had three patterns: locally extensive and centred on the cranioventral part of the lungs (*n* = 5), diffuse across both lungs (*n* = 3), or multifocal in both lungs (*n* = 1). In these animals, lesions were usually purple (other colours were red, grey, or black), heavy, firm, and swollen. On section, the lesional lung tissue was well-demarcated from normal tissue, bulged, and exuded opaque, watery red or viscous yellow fluid. Samples of lesional lung tissue just floated or sank in water. In the one animal with multifocal lesions, some of the lesions had formed abscesses.

##### Histology

Lung lesions associated with bacterial infections typically were centred on the bronchioles. In the lumina and walls of alveoli, bronchioles, and bronchi, there were diffuse or multifocal aggregations of many neutrophils and fewer macrophages, often mixed with oedema fluid, fibrin, erythrocytes, and variable numbers of bacteria. In some animals, the central parts of focal lesions were necrotic and there was lysis of neutrophils. The lesions were typically diagnosed as acute, multifocal, suppurative bronchopneumonia.

#### Fungal pneumonias (n = 7):

##### Gross lesions:

Lung lesions associated with fungal infections varied in gross appearance. In two animals, no gross lesions suggestive of fungal infection were observed. Where fungus-associated lesions were seen (*n* = 5), they usually consisted of multiple, round or irregular, yellow, well-demarcated, raised, firm nodules, which ranged from 3 to 6 cm in diameter, and sank in formalin. On section, the lesional lung tissue exuded yellow to yellow-green, opaque, viscous fluid (pus), except for one animal, where it was dry and pink (necrosis). In another animal, the lesions were walled off by a 0.5-cm-thick layer of connective tissue (abscesses).

##### Histology:

Lung lesions associated with fungal infections typically consisted of focal or locally extensive areas of inflammation and necrosis in the lung parenchyma. The lumina of alveoli and bronchioles contained many neutrophils and macrophages, mixed with erythrocytes, fibrin, oedema fluid, and cellular debris. Within the inflamed tissue, there were one or more foci of necrosis of both lung tissue and inflammatory cells. Also, within the lesional tissue, there were multiple parallel-walled, septate fungal hyphae of 5 to 10 microns thick. Some hyphae split into two branches of equal thickness at an angle of less than 90 degrees, and a few hyphae had fruiting bodies (*Aspergillus* sp.). Fungal hyphae were detectable by H&E stain, but much more obvious by Grocott stain. In one animal fungal structures included buds which were reminiscent of a cryptococcus infection. Culture of infected tissue however resulted in the growth of *Aspergillus fumigatus.* In one animal, the largest focal lesion was partially encapsulated by fibrous connective tissue. In another animal, the lesion extended to the pleura, which was diffusely thickened by many macrophages. The lesions were typically diagnosed as chronic, multifocal or coalescing, pyogranulomatous, necrotizing pneumonia.

Pathology of the pulmonary vasculature

In 29 out of the 57 adult and juvenile harbour porpoises a *Pseudalius inflexus* infection of the pulmonary vasculature was observed. Only in animals with a *Pseudalius inflexus* infection were lesions of the pulmonary vasculature observed. In one animal these lesions were considered to be severe (and thus responsible for stranding). No gross lesions of the pulmonary vasculature were observed with the exception of the presence of specimens of *Pseudalius inflexus*. Histologic lesions associated with *Pseudalius inflexus* infection of the pulmonary vasculature were: phlebitis (*n* = 9), semi obstructive infestation (*n* = 1), smooth muscle hyperplasia, multifocal, chronic and marked (*n* = 1) and thrombosis (*n* = 2). Phlebitis was further characterized as diffuse and fibrosing (*n* = 1), suppurative (*n* = 1), proliferative (*n* = 2). Thrombosis was further characterized as suppurative (*n* = 1) and partly to completely obliterative, chronic, active associated with focal fungal infection.

### CNS pathology

#### Fungal infection (n = 3)

##### Histology

Brain lesions associated with fungal infections were not detected grossly, but only by histological examination. In all three cases, the causative fungus had morphological characteristics typical for *Aspergillus* sp. In the more marked lesion, a large part of the tissue section of cerebellum was necrotic and largely replaced by neutrophils and macrophages, with aggregates of lymphocytes around remaining blood vessels and multiple haemorrhages. The overlying pia mater and dura mater was thickened due to fibroplasia and infiltration by lymphocytes, plasma cells, macrophages, and neutrophils. Throughout the affected tissue, and clearly demonstrated by Grocott stain, were fungal hyphae, characterized by parallel walls, septa, and acute branching at about 30 degrees. Additionally, by Gram stain, multiple aggregates of Gram-positive coccoid and rod-shaped bacteria were present. In the less marked lesion, there were multiple aggregates of neutrophils and macrophages centred on blood vessels in the white matter, and hyphae characteristic for *Aspergillus* sp. in blood vessel walls. In the third animal, which had an *Aspergillus*-infection-associated otitis media (see below), *Aspergillus*-like hyphae were detected at the edge of the tissue section of cerebellum, without associated pathological changes.

#### Viral infection (n = 1)

##### Histology

Brain lesions associated with virus infection were detected in one case. No gross changes were seen in the brain, but histologically in the cerebrum, there was a locally extensive area of increased density of nuclei in the grey matter. In this area, there were perivascular aggregates of lymphocytes and randomly scattered neutrophils in the neuropil. In multiple neurons, the cytoplasm was eosinophilic and the nuclei had large amphophilic inclusion bodies and marginated chromatin. A novel herpesvirus, tentatively named *Phocoena phocoena* herpesvirus type 2, PPHV-2, was identified in brain samples of this animal. The lesion was diagnosed as a subacute, locally extensive, lymphocytic encephalitis with neuronal necrosis and intranuclear inclusion bodies. The findings of this case have been published previously [23].

#### Inflammation of unknown aetiology (n = 3)

##### Gross lesions:

No abnormalities detected.

##### Histology

Three harbour porpoises had brain lesions, for which no aetiology was found. None of these three animals had gross brain lesions, only histological lesions. In the first case, these were diagnosed as moderate, subacute, diffuse, lymphocytic encephalomyelitis, with multifocal gliosis, perivascular lymphocytic cuffing, oedema, and (in the cerebellum) loss of Purkinje cells. In the second case, they were diagnosed as mild, subacute, multifocal, polioencephalitis, and were characterized by the presence of small aggregates of nuclei centred on neurons (neuronophagia) in the cerebrum. In the third case, they were also limited to cerebrum and diagnosed as moderate, subacute, focal, lymphocytic meningo-encephalitis, with neuronophagia.

Beside the six animals described above, CNS involvement was suspected in two more animals. Both animals displayed clinical nervous signs. No gross or histologic lesions were observed in the samples of either animal. One animal had an *Aspergillus fumigatus* infection of the respiratory tract and had epileptic seizures. The other animal had herpesvirus DNA isolated from the brain and cornea and made abnormal uncoordinated swimming movements and had a vertical nystagmus of the right eye.

### Liver pathology

#### Non- inflammatory lesions

In the first case, a pregnant animal with a 60-cm-long male foetus, the liver had an irregular surface and rounded edges. It was diffusely yellow-pale tan and had an increased lobular pattern, both on surface and on section. Histologically, there were areas of necrosis around hepatic venules, characterized by an outer zone of shrunken hypereosinophilic hepatocytes with small dark purple nuclei and a central zone of separated hepatocytes with indistinct cell borders and pale or absent nuclei (karyolysis). Hepatocytes throughout the tissue section had abundant lipid vacuoles in the cytoplasm. These lesions were diagnosed as marked, acute, periacinar hepatic necrosis and moderate, diffuse hepatic lipidosis. The aetiology was not determined, but the lesions resemble those of fatty liver syndrome in cats and equine hyperlipemia in horses (references).

In the second case, a neonate, the liver bulged and had rounded edges. Histologically, the hepatocytes throughout the tissue section contained large smooth-edged cytoplasmic vacuoles, some of which had coalesced into large multilocular vacuoles. In the renal cortex of the same animal, the tubular epithelial cells throughout the tissue section had similar cytoplasmic vacuoles. Kidney and liver weights were not exceptional compared to other animals of similar length. The aetiology was not determined, but the lesions were suggestive of lysosomal storage disease (e.g., glycogen storage disease type 1) or intoxication (e.g., Swainsona).

#### Bacterial infection

Liver lesions associated with a bacterial infection were observed in one animal, a juvenile that had bacterial disease in multiple other organs as well as, terminally, a bacterial sepsis. Grossly in the liver, there were two well-demarcated abscesses of 1 cm diameter and filled with yellow-green viscous fluid. Histologically, there were two coalescing abscesses, with a thick wall of fibrous connective tissue around a core of many neutrophils and a rare trematode egg (*Campula oblonga*). A pure culture of *Streptococcus dysgalactiae* was isolated from an abscess sample.

Findings on parasitic infections of the digestive tract

*Campula oblonga* infection of the liver and *Anisakis simplex* infection of the forestomach occurred significantly more often in adults than in juveniles (*p* < 0.05, Fisher test two sided). The prevalence of *C. oblonga* infection of the pancreas, *Pholeter gastrophilus* infection of the pyloric stomach and *Diphyllobothrium stemmacephalum* of the intestine did not differ significantly between juveniles and adults (*p* > 0.05, Fisher test two sided). Prevalences of parasitic infections of organs of the digestive tract are available in Additional file 1 (Tables 7 and 8). The estimated age of the youngest animals with parasitic infections of the digestive tract were: 9 months for *C. oblonga* infection of the liver, adult (of unknown age) for *C. oblonga* infection of the pancreas, 7 months for *A. simplex* infection of the fore stomach, 9 months for *P. gastrophilus* infection of the fundic stomach and 11 months for *D. stemmacephalum* infection of the intestine.

## Organ sizes and weights (relative to body length) in relation to lesions observed

## The R^2^ was low for spleen (0.17), ratio left ventricle width vs right ventricle width 0.18), ovary (0.34), and left ventricle width (0.42) indicating a poor relation between body length and variable measured. R^2^ was intermediate for brain (0.57) and adrenal weights (0.65). R^2^ was high for liver (0.77), lung (0.81), kidney (0.83), heart (0.88) and body weight (0.91). For raw data please see Additional file 2.

Comparing highest and lowest 5% organ sizes (in relation to body length) or the three cases with the highest weight and the three cases with the lowest weight, to diagnosis made indicated: low body weight coincided with findings of emaciation (3 out of 3) and high body weight with findings of good nutritional condition ( 3 out of 3); high brain weight coincided with diagnoses of encephalitis (2 out of 3); high lung weight coincided with pulmonary congestion (3 out of 3), see also table 8 (incidental diagnoses); low liver weight coincided with cholangitis (2 out of 3); and high adrenal gland weights coincided with hyper- and hypo- plasia of the adrenal gland (2 out of 3). Detailed data are available in excel file Graphs and raw data of organ weights and sizes in additional file 3.

The relation between body length and heart ventricular wall width ratio (L/R) showed four clear outliers out of sixty measurements (see Figure 1).

The four cases of an increased LV width/ RV width ratio were all due to a decreased right ventricular wall width. Two juveniles and two adults were involved. All four animals were infected with *P. inflexus* and had pulmonary vasculature lesions ranging from an obstructive thrombus in a pulmonary artery to hyperplastic diffuse chronic vasculitis. In two cases, death was sudden and unforeseen. In one of these two cases, no cause of death was found by autopsy.

The likelihood that all animals with a high LW width/ RV width ratio were randomly chosen from the population and all had histologic lesions in the vasculature was not significant (*p* > 0.05, Fisher test).

**Figure 1 Ratio Left cardiac ventricular width over right cardiac ventricular width in relation to body length**

Observations on right ventricle wall thinning

We observed right ventricular wall thinning in four harbour porpoises (additional file Figure 1). The circumstantial facts were suggestive of pulmonary hypertension caused by *P. inflexus* infection of the pulmonary vasculature, leading to dilated cardiomyopathy of the right ventricle. Statistical significance of the association of right ventricular wall thinning and histologic lesions of pulmonary vasculature was weak (*p* = 0.16 for adults and *p* = 0.08 for adults, Fisher test), possibly due to small sample size. No supporting evidence exists from other species with a likewise reaction of the right ventricle to pulmonary hypertension. The connection between right ventricular dilation and infection of the pulmonary vasculature by *P. inflexus* therefore remains rather speculative. If future research opportunities present it would be interesting to do extensive diagnostics on harbour porpoise hearts to investigate this relationship.

**Table** **1 Individual animals with their lesions distributed into lesions which contributed to stranding, did not contribute to stranding or were acquired after stranding, with comment interpreting the severity of lesions.**

| **Erasmus code number** | **Lesions which did not contribute to stranding** | **Lesions which contributed to stranding** | **Lesions associated with agony (euthanasia) or acquired after stranding** | **Comment** |
| --- | --- | --- | --- | --- |
| PP030320 | • Oral cavity: ulcerative stomatitis, necrotizing, multifocal, acute, moderate.  • Penis and preputium: balanoposthitis, suppurative, ulcerative, acute, severe.  3) Incidental diagnoses:  • Trachea, bronchi: nematode infection (probably Stenurus minor)  • Lung: pulmonary oedema, diffuse, acute, moderate.  • Lung: pulmonary congestion, diffuse, acute, moderate.  • Oesophagus: ulcerative oesophagitis, multifocal, acute, mild.  • Stomach, first section: nematode infection.  • Stomach, third section: gastritis, granulomatous, focal, chronic, probably associated with a trematode infection (Pholeter gastrophilus).  • Bile ducts: cholangitis, fibrosing, diffuse, chronic, moderate, associated with trematode (Campula oblonga) infection.  • Ear, bulla tympanica: nematode infection (probably Stenurus minor).  • Skin: ulcerative dermatitis, multifocal, chronic, mild.  • Spleen: splenic atrophy, marked. | • General: emaciation, severe. |  | This carcass of a juvenile male harbour porpoise was in poor nutritional condition and freshly dead. The most severe lesion observed at gross necropsy was the severe emaciation. In absence of an obvious underlying cause, lack of food (simple starvation) seems to be the most likely cause. However, further laboratory analyses will be performed to rule out the presence of underlying disease.  The differential diagnosis for ulcerative stomatitis, oesophagitis, and balanoposthitis includes herpesvirus and morbillivirus infections. The parasitic infections in lungs, bile ducts, stomach, and ears are common in harbour porpoises and probably had little effect on this animal’s health. |
| PP030405 | • Lung: pulmonary oedema, diffuse, acute, moderate.  • Lung: pulmonary congestion, diffuse, acute, moderate.  • Pneumonia, granulomatous, focal, chronic, mild, associated with small lungworm infection  • Alveolitis, fibrinous, multifocal, acute, mild.  • Tracheitis, suppurative, superficial, diffuse, acute, very mild  • Nematode infection (small lungworm) of the alveolar lumina.  • Lymph nodes, tracheo-bronchial and lung associated: lymphadenopathy, mild.  • Ear, bulla tympanica: nematode infection (probably Stenurus minor).  • Dermatitis, superficial, multifocal, mild | • Cornea and conjunctiva, bilateral: keratoconjunctivitis, ulcerative, bilateral, chronic, severe.  • Right eye: keratitis, fibrosing, diffuse, chronic, marked, associated with corneal perforation  • Pneumonia, pyogranulomatous, multifocal, chronic, severe, associated with bacterial infection. |  | This carcass of a juvenile female harbour porpoise was in good nutritional condition and freshly dead. The most severe lesion observed at gross necropsy was the bilateral keratoconjunctivitis. No gross lesions were observed in the central nervous system to explain the nervous signs displayed during rehabilitation, but such lesions often only are visible microscopically. The differential diagnosis for ulcerative keratoconjunctivitis includes herpesvirus infection. The severity of the pulmonary lesions was difficult to assess macroscopically. Upon histology these lesions were assessed as relevant. |
| PP031124.2 | • Pulmonary oedema acute severe diffuse  • Pulmonary hypostatic congestion focal mild  • Oral mucosal haematoma focal mild  • haemo/hydro pericardium acute mild  • Renal pelvic mineralization, multifocal, mild | - Emaciation |  | This female juvenile harbour porpoise was found on the tenth of March 2003 on the beach at Cadzand (Zeeland). After three days of intensive care the animal died. The last 36 hours its condition worsened, i.e. the animal started to vomit and became progressively subdued and was not able anymore to swim without human support. On necropsy the most significant finding is a marked general emaciation. The blubber layer is extremely thin, there is no fat around the internal organs and the back around the dorsal fin is markedly concave. In the pericardium 10 cc of red watery fluid is found. The significance of this finding is unclear. A disease that either uses a lot of energy or inhibits sufficient energy uptake by interfering with digestion or food uptake can cause emaciation. Harbour porpoises are weaned between 8 and 12 months after birth. This is a crucial period where they are extra vulnerable and have to prove they can be self-supporting. Thus, emaciation can also be caused by lack of foraging capabilities. Finally, emaciation can be caused by a general lack of food. In this animal the cause for emaciation was not evident from the macroscopic necropsy. There was no evidence of pneumonia or aspiration of food remains. |
| PP040324 | • Tracheitis, lymphocytic, diffuse, chronic, moderate, with epithelial hyperplasia and squamous metaplasia.  • Bronchitis, lymphocytic, diffuse, chronic, moderate, with epithelial hyperplasia and squamous metaplasia.  • Bilateral general pulmonary congestion, mild  • Infestation of the upper respiratory tract with large lungworms (T convolutus or P inflexus) Moderate chronic, bilateral multifocal.  • Wound in front of dorsal fin: Fasciitis and panniculitis, focal, moderate, chronic, with formation of fibrovascular tissue.  • Tracheo-bronchial and mesenteric lymph nodes: Lymphoid hyperplasia, benign.  • Tracheo-bronchial and mesenteric lymph nodes: lymphadenitis, eosinophilic, diffuse, chronic, mild.  • Two ulcers on mucosa of medial rostral mandible, multifocal, chronic and mild  • Ulcer on right side inside genital slit, focal, chronic mild  • Pancreatic duct hyperplasia, diffuse, chronic, marked.  • Duodenal ampulla: abscess, associated with parasite infection (likely Pholeter gastrophilus). | • Bronchopneumonia histiocytic, suppurative, multifocal, chronic-active, marked, associated with lungworm infection.  • Cerebrum: meningitis, non-suppurative, segmental, chronic, moderate.  • Cerebrum: neuronal necrosis, segmental, moderate | • Bilateral general pulmonary oedema, acute and severe. | This carcass of a subadult male harbour porpoise was in poor to moderate nutritional condition and freshly dead.  The most significant lesions were a multifocal puro granulomatous pneumonia and moderate neuronal necrosis and meningitis of the cerebrum associated with herpes virus DNA. The foci in the lungs were mostly associated with the remnants of large lungworms (length 10 – 30 cm). The lung worm infestation was bilateral and moderate to severe (100-1000 lungworms per lung). The cause of the large wound in front of the dorsal fin was unclear but trauma seems a likely cause. The wound had not penetrated the blubber layer. The surface consisted of repair tissue which means the wound was at least several days old. The cause of the ulcers in the oral cavity and the genital slit is not known. |
| PP040517 | • Moderate chronic corneal ulcus on left eye  • Pulmonary-associated lymph node: lymphoid hyperplasia, moderate  • Pulmonary blood vessels: nematode infection  • duodenal submucosal abscess, focal, chronic, mild.  • colonic crypt abscesses, mild  • Mild chronic ulcus on inner side left genital slit  • Mild chronic ulcus on rostral tip of palatum durum. | • bronchopneumonia suppurative, multifocal, chronic-active, associated with lungworm larvae.  • pneumonia, necrotizing, focal, acute, mild, associated with Aspergillus fungal hyphae |  | This male juvenile harbour porpoise in moderate condition was euthanized after repeated epileptiform attacks. The primary cause of his clinical symptoms is most likely a subacute severe diffuse pneumonia. The general pulmonary oedema might have resulted from the euthanasia. Alternatively, the oedema might be the result of heart failure or damaged alveolar type 1 epithelium due to systemic toxins, endotoxins or shock like states. The interstitial emphysema indicates the porpoise had experienced severe respiratory difficulties. The gas in the lymph vessels indicates the emphysema was not peracute and did not result from the euthanasia. No abnormalities were detected in the central nervous system upon macroscopic examination which could have been responsible for the epileptiform attacks. |
| PP040526 | • Patent urachus  • Muscular petechiae multiple, localized acute, moderate.  • Hepatic lipidosis, diffuse, mild.  • Liver congestion, diffuse mild and acute.  • Subepithelial oesophageal congestion, diffuse, acute mild  • Mucosal fore stomach congestion, diffuse acute mild  • Meningeal congestion, diffuse, acute mild  • Patent ductus botallicus | • Interstitial pneumonia, suppurative, multifocal, acute, moderate. | • Pulmonary oedema, acute, diffuse, bilateral, severe | This neonatal male harbour porpoise most likely has died of suffocation due to severe pulmonary oedema and the interstitial pneumonia. Neurogenic shock resulting from transportation stress is a cause that has to be considered. The petechiae found on the thoracic wall support this speculation. The significance of the congestion in oesophagus, fore stomach and meninges is uncertain. The patent urachus did not lead to a bacterial invasion and sepsis, based on the negative findings of the bacterial examination. |
| PP040627 | • Bronchopneumonia, histiocytic, suppurative, diffuse, chronic, mild.  • Fore stomach, glandular stomach: foreign body (both stomachs five ml  of sand)  • Omphalitis, haemorrhagic, suppurative, focal, acute, mild.  • Moderate diffuse acute liver congestion |  | • Acute moderate diffuse bilateral pulmonary oedema. | This male neonatal harbour porpoise was euthanized within twelve hours of being admitted in the rehabilitation centre. No evident cause was found for the severe clinical symptoms that were seen. The sand in the  fore stomach and the glandular stomach might have been caused by a rough stranding on a sandy beach. The pulmonary oedema and the liver congestion are normally seen in carcasses of euthanized animals. The omphalitis and pulmonary infection seem clinically insignificant. |
| PP041215 | . • Pulmonary blood vessels: vasculitis, hyperplastic, diffuse, chronic, moderate, associated with nematode infection.  • Keratitis focal chronic moderate  • Lung: nematode infection | • pneumonia, necrotizing, suppurative, locally extensive, acute, marked, associated with bacterial infection  • bronchitis, bronchiolitis and alveolitis, granulomatous, suppurative, multifocal, chronic, moderate, associated with adult and (especially) larval nematodes.  • Sepsis |  | This juvenile male in poor nutritional condition died after two days in the rehabilitation centre. The most significant finding was a focal pneumonia in the right side of the lungs associated with parasites and a bacterial infection which had spread throughout the body (sepsis) |
| PP050208 | • Pulmonary artery: arteritis, proliferative, multifocal, chronic, moderate, associated with nematode infection.  • Lung-associated lymph node: lymphoid hyperplasia, moderate.  • Lung-associated lymph node: lymphadenitis, eosinophilic, diffuse, subacute, moderate.  • Adrenocortical hyperplasia bilateral, chronic, severe  • Pancreatic angitis, proliferative, eosinophilic, diffuse, chronic, marked, associated with parasitic infection  • Cholangitis, proliferative, eosinophilic, diffuse, chronic, marked, associated with parasitic infection.  • Skin lesions, multifocal scars, healed  • Skin lesions, multifocal discolourings, chronic mild (epidermal intracytoplasmic eosinophilic inclusions (poxvirus))  • Intestine, hypostatic congestion, mild  • Peritoneal cyst, focal mild  • Subcutal inflammation multifocal chronic mild  • Renal visceral discoulorings one sided multifocal, mild  • Urinary vesical wall congestion diffuse, mild  • Pulmonary hypostatic congestion  • Pulmonary purulent inflammation, focal mild  • Bronchiolar parasitic infestation, multifocal, chronic mild | • Bronchopneumonia, mixed, multifocal, chronic-active, marked, associated with two nematode species and suspect microbial infection | • Pulmonary oedema, moderate, acute diffuse.  • Pulmonary haemorrhage, focal, acute moderate | This adult female harbour porpoise in good body condition died during a diagnostic bronchoscopy. The most important findings were a bronchopneumonia, possibly caused by a bacterial infection (not cultured) and or a fungal infection (cultured but not clear on histology) and a severe parasitic infestation of the pulmonary vessels and a moderate haemorrhage of the right lung. The parasitic infection might have had a strong negative effect on the perfusion of the lung thus causing hypoxia and in combination with the bronchopneumonia it caused an inability of the animal to dive. If the animal already failed to dive when at sea this could explain all the scars and wounds found at stranding by bird pecking. The existing hypoxia was not helped by the sedation which decreases breathing rate. The haemorrhage and the stress of the intervention adding to the negative existing hypoxia might have caused fatal hypoxia leading to shock or brain damage and apnoea. The large adrenals might have been caused by chronic stress. All other findings are incidental. |
| PP050502 | • Bronchitis, lympho-plasmacytic, suppurative, diffuse, subacute, moderate  • Lymph node, prescapular (?): Lymphoid hyperplasia, moderate  • Pleuritis multifocal unilateral chronic moderate  • Pericarditis, focal chronic moderate  • Lymfonodular enlargement pulmonary multifocal chronic moderate  • Cystitis, eosinophilic, diffuse, superficial, acute, mild.  • Mesenteric lymph node: lymphoid hypoplasia, moderate.  • Inflammation of the pancreatic duct, lympho-plasmacytic, fibrosing, diffuse, chronic, moderate  • Adrenocortical hypoplasia, diffuse, marked, associated with exogenous corticosteroid therapy  • Papilloma, with characteristic basophilic intranuclear viral inclusion bodies (herpesvirus)  • Gastritis, lympho-plasmacytic, eosinophilic, superficial, multifocal, subacute, mild  • Dermatitis, multifocal, suppurative, superficial, acute, moderate, associated with bacterial infection.  • Dental abrasion multifocal chronic mild  • Mesenterial lipoma’s multifocal mild  • Hepatitis multifocal chronic mild and associated with the presence of trematodes  • Vaginal mucosal lesions multifocal chronic mild  • Unilateral corneal oedema focal mild  • Dermal papilloma Focal chronic mild  • Mammarian cysts multifocal chronic mild  • Thrombosis (fundic stomach), focal, reorganized, recanalized  • Colitis, eosinophilic, lympho-plasmacytic, superficial, diffuse, chronic, mild. | • Alveolitis, histiocytic, multifocal, chronic, moderate to severe, with abundant foamy material containing small round pink bodies about 0.5 μm diameter  • Dermatitis ulcerative multifocal chronic moderate to severe  • Panniculitis multifocal mild | • Pulmonary oedema acute diffuse mild  • Kidney failure leading to unacceptable clinical symptoms and euthanasia  • Nephritis, suppurative, focal, acute, mild | This adult female harbour porpoise in moderate body condition was euthanized after 1 ½ years of treatment at the rehabilitation centre of the Dolfinarium Harderwijk. The animal succumbed to kidney failure, which was most evident in the blood serum values. Urea increased from 18 to 30 mmol/L, creatinine from <27 to 81 μmol/L and sodium from 158,5 to 196,4 mmol/L. The animal suffered from infections in multiple locations, dermatitis, cystitis, nephritis, bronchitis, pancreatic duct inflammation, colitis and gastritis. The dermatitis was the most severe. An ascending cystitis might be responsible for the acute nephritis, which caused severe clinical symptoms. The respiratory tract was despite enduring treatment not free from chronic (bacterial) infection as demonstrated by the chronic pleuritis, miliary abscesses in the rostral left lobe and the suppurative bronchitis. The pulmonary alveolar proteinase which was diagnosed per exlusionem based on histology is in humans known to cause chronic pulmonary infections and may increase the susceptibility of all organ systems to infection due to the failure of granulocyte macrophage colony stimulating factor (GM-CSF) negatively influencing the immune reaction. The applied anti-parasitic treatment was apparently effective only 2 live nematodes were found in the pulmonary venae. The diagnose of pulmonary alveolar proteinase needs to be confirmed by electron microscopy. The genital lesion which had the outward appearance of a papilloma was associated with the presence of herpes virus like particles and a herpes positive PCR |
| PP050610 | • Trachea, bronchus: tracheobronchitis, superficial, chronic, diffuse, with multifocal squamous metaplasia of respiratory epithelium.  • Pulmonary parasitic pneumonia multifocal chronic mild  • Hepatic congestion diffuse acute moderate  • Thyroid gland: hyperplastic goitre or thyroid follicular atrophy (dependent on thyroid weight).  3) Incidental diagnoses:  • Multifocal dermal scarring chronic mild (healed)  • Multifocal dermal nodules chronic mild  • Lung: small lungworm infection (probably Stenurus minor) in lung parenchyma.  • -Pulmonary artery: arteritis, multifocal, chronic, mild, with thrombosis, associated with heartworm infection (probably Torynurus convolutus).  • -Oesophageal artery: arterial mineralization.  • Pulmonary associate lymph node: lymphoid hyperplasia, diffuse. | • Multifocal granulomatous infection of diverse organs and serosa of the thorax chronic severe, associated with Aspergillus infection  • Right and left heart ventricle: epicarditis and myocarditis, granulomatous, necrotizing, multifocal, chronic, moderate, associated with Aspergillus infection.  • Granulomatous myocarditis multifocal chronic severe  • Pyothorax bilateral mild  • Lung: bronchopneumonia, necrotizing, haemorrhagic, granulomatous, multifocal or diffuse, chronic, marked, associated with the presence of Aspergillus hyphae.  • Pleura: pleuritis, granulomatous, multifocal or diffuse, moderate or marked, associated with the presence of Aspergillus hyphae.  • Granulomatous mediastinitis multifocal chronic moderate  • Vertebral osteomyelitis focal chronic moderate | • Pulmonary oedema general acute severe  • Vertebra: osteomyelitis, pyogranulomatous, focal, chronic, marked, with exophytic bone formation, associated with bacterial infection.  • -Skeletal muscle at injection site: muscle necrosis and haemorrhage, focal, acute, marked, associated with imipenem injection. | COMMENTS: This juvenile female harbour porpoise stranded in moderate body condition. The most significant finding was a multifocal granulomatous infection of multiple organs and serosa of the thorax, associated with an Aspergillus fumigatus infection. The infection likely originated from the lungs and spread per continuitatum to the pleura and pericardium. A possible osteomyelitis of the caudal vertebral column might have caused deterioration of the condition and resistance of the harbour porpoise or might have occurred as a result of the disease process causing the animal to be less rapid and evasive of predators. |
| PP050825.1 | • Vasculitis, granulomatous, with endothelial hyperplasia, fibrosis, and thrombosis, moderate, associated with nematode infection.  • Bronchitis, lymphoplasmacytic, locally extensive, chronic, moderate, associated with large lungworm infection.  • Pulmonary blood vessels: vasculitis, suppurative, associated with bacterial infection superimposed on a nematode infection.  • Pulmonary lymph node: lymphoid hyperplasia, moderate.  • Dermal lesion focal mild  • Dermal trauma lesions multifocal acute mild  • Gastritis multifocal chronic mild | • Pneumonia, granulomatous, multifocal, chronic, moderate, associated with large lungworm infection  • Pneumonia, histiocytic, eosinophilic, locally extensive, chronic, moderate, associated with small lungworm larvae. | • Pulmonary oedema | This juvenile male harbour porpoise in good nutritional state died during transport to the rehabilitation centre. Cuts on the tail fluke and abundant pulmonary oedema were found and are in accordance with bycatch. Empty stomachs, absence of chyle in the mesenteric lymphatic vessels, are not in accordance with bycatch. The severe pulmonary oedema is the probable cause of death. Possibly the oedema was caused by increased oxygen requirement due to transport stress in combination with poor ventilation and perfusion due to severe parasite infestation of bronchi and pulmonary arteries plus abnormal posture. The good body condition argues however against continuous respiratory distress as the harbour porpoise had been able at least until very recently to feed itself adequately. |
| PP050825.2 | • Pulmonary artery: arteritis, proliferative, multifocal, chronic, associated with nematode infection (likely Pseudalius inflexus).  • Phlebitis verminous multifocal chronic mild  • Gastric granuloma, focal, chronic, mild, associated with encapsulated parasite (likely Pholeter gastrophilus). | • Encephalitis, lymphocytic, locally extensive, subacute, with neuronal necrosis and intranuclear inclusion bodies (herpesvirus).  • Bronchopneumonia, pyogranulomatous, multifocal, chronic, associated with nematode infection (probably Stenurus minor). | • Pulmonary oedema diffuse acute severe | This female juvenile harbour porpoise in poor nutritional condition was euthanized due to aggravating dyspnoea. The most important morphologic diagnosis is the pyogranulomatous bronchopneumonia, which in itself was probably not severe enough to cause the apparent dyspnoea, the arteritis of the pulmonary artery which may have caused severe perfusion ventilation mismatch which might explain the dyspnoea. The third important morphological diagnosis was encephalitis which was likely caused by herpesvirus. Herpesviruslike particles were observed by electron microscopy in lesions of the cerebrum and herpesvirus presence was confirmed by polymerase chain reaction on samples of the cerebrum. Alternatively, the dyspnoea was a behavioural aberration caused by the herpesvirus encephalitis. Shivering may have been caused by the need of the body to heat up or alternatively by the encephalitis. Shivering was also noted in a white beaked dolphin with morbillivirus encephalitis. The pulmonary oedema is most likely to have been caused by the euthanasia. |
| PP051106 | • Trachea: tracheitis, lymphoplasmacytic, superficial, diffuse, subacute, moderate.  • Bronchus: bronchitis, lymphoplasmacytic, superficial, diffuse, subacute, moderate..  • Bronchiole: bronchiolitis obliterans, focal, chronic, marked, associated with nematode infection (possibly Pseudalius inflexus).  • Adrenal cortex: adrenocortical haemorrhage, multifocal, acute, moderate.  • Forestomach: gastritis, ulcerative, multifocal, acute, mild to marked, associated with bacterial and parasitic infection.  • Fundic stomach: gastritis, fibrosing, focal, chronic, associated with trematode infection (probably Pholeter gastrophilus).  • Bile duct: cholangitis, lymphoplasmacytic, locally extensive, chronic, marked, with epithelial hyperplasia and fibrosis, associated with trematode infection (probably Campula oblonga)  • Anaemia diffuse moderate  • Dermal lesions multifocal acute mild  • Dermal lesions multifocal chronic mild | • Exhaustion and emaciation  • Pulmonary blood vessel: thrombosis, partly to completely obliterative, chronic-active, marked, associated with large nematode and (focally) fungal infection.  • Pulmonary blood vessel: vasculitis, hyperplastic, lymphoplasmacytic, diffuse, marked, chronic, associated with large nematode infection (possibly Pseudalius inflexus).  • Multifocal atelectasis subacute to chronic moderate  • Lung: pulmonary oedema and congestion, diffuse, acute, moderate |  | This pregnant female harbour porpoise in poor body condition probably died due to exhaustion and emaciation. The most relevant lesions were the subacute to chronic diffuse pneumonia and bronchitis obliterans caused by nematode infection (Pseudalius inflexus) plus the marked vasculitis and obliterative thrombosis of the pulmonary vasculature, associated with Pseudalius inflexus infestation and fungal infection. The multiple gastric ulcers will have caused chronic blood loss and the observed anaemia will have been partly due to these ulcers. Toxic substances from the chronic inflammation of the lungs might also have contributed to the observed anaemia. The anaemia in combination with the small function lung capacity left must have caused poor oxygenation of all organs. The multifocal hepatitis is probably due to a C. oblongata infection although no trematodes could be found. The relevance of the pancreatic cyst is unclear. In humans a cyst can be caused by acute or chronic inflammation or be a benign or malignant tumour. Histology might give more clues towards the interpretation of this lesion. The dermal lesions are insignificant. The linear scar on the right side of the head could be caused by the head of the porpoise being stuck in a net but this is very speculative. |
| PP060220 | • Pulmonary artery: thrombosis, chronic, moderate  • Keratitis focal acute mild  • Hepatic lipidosis diffuse acute to chronic moderate associated with possible physiologic gravid hepatic lipidosis  • Hepato cholitis chronic multifocal to coalescing mild  • Pneumonia, chronic-active, multifocal, mild.  • Dermatitis, suppurative, superficial, focal, acute, mild.  • Skin: dermal haemorrhage, focal, subacute, mild.  • Fundic stomach: gastric erosion, focal, acute, mild | • Cardiac malformation unilateral acute to chronic severe  • Skeletal muscle degeneration, diffuse, subacute, marked. |  | This gravid female harbour porpoise died after 4 days in a rehabilitation centre. The main findings were a fatty liver and an abnormal heart with abnormal ratio of left and right ventricle thickness and a right ventricle which had less consistency than normal. A fatty liver is physiologic in gravid dolphins but could also be a sign of acetonemia. Acetonemia would possibly explain the observed spasms and the poor coordination during swimming. However, liver enzymes were not as elevated as in most other strandings and there was no typical smell of ketone bodies which has been observed several times in stranded harbour porpoises. Heart failure might have been responsible for a deteriorated fitness and thus less ability to forage, hence the poor body condition. It might also explain the rather unexpected death of the animal. |
| PP060227 | • Tracheobronchial and mediastinal lymph node: lymphoid hyperplasia, diffuse, moderate to marked  • Keratitis, suppurative, diffuse, chronic-active, marked, with vascularisation, pigmentation, and retrocorneal membrane  • Cellulitis multifocal acute to chronic severe  • Pulmonary inflammation chronic focal mild  • Cholangitis, lymphoplasmacytic, locally extensive, chronic, marked, with bile duct hyperplasia and fibrosis, associated with trematode (Campula oblonga) infection.  • Pulmonary vein: phlebitis, lymphoplasmacytic, diffuse, chronic, very mild, associated with nematode infection  • Blubber: panniculitis, histiocytic, multifocal, chronic, mild.  • Lung: pulmonary granuloma, chronic, mild, associated with lungworm infection.  • Lung: bronchitis, lymphoplasmacytic, diffuse, chronic, mild.  • Right epididymis: Epididymal cyst, unilateral, chronic, marked. | • Lung: bronchopneumonia, suppurative, locally extensive, acute, moderate  • Cerebrum: encephalitis, lymphocytic, diffuse, subacute, moderate, with multifocal gliosis, perivascular cuffing, and oedema.  • Cerebellum: meningo-encephalitis, lymphocytic, subacute, moderate, with multifocal gliosis and loss of Purkinje cells.  • Cervical spinal cord: myelitis, lymphocytic, diffuse, subacute, moderate. | • Pulmonary oedema diffuse acute marked  • Pulmonary congestion diffuse acute moderate | This male harbour porpoise in good nutritional condition died after 16 days in a rehabilitation centre. The most significant lesion is the encephalitis. It is restricted to the grey matter (so, polioencephalitis), and is characterized by neuronal necrosis, foci of gliosis and oedema, and perivascular lymphocytic cuffing. This is typical of a viral or protozoal infection, and because no zoites or cysts are seen, protozoal infection is less likely. A viral infection may be responsible for the polioencephalitis. The pulmonary pathology will have contributed to the demise of the animal. |
| PP060301 | • Corneal perforation, bilateral, with iris prolapse, severe diffuse suppurative keratitis, corneal vascularization, and corneal pigmentation.  • Hepatic atrophy, diffuse, marked.  • Hepatic bile duct cysts, multifocal, coalescing  • Dermatitis, hyperplastic, locally extensive, chronic, moderate, with intracytoplasmic inclusion bodies (poxvirus).  • Dermatitis, suppurative, multifocal, superficial, acute, mild  • Bronchopneumonia, suppurative, multifocal, peracute, mild, associated with bacterial infection  • Small intestine inflammation focal acute mild.  • Bronchiolar infection with nematode parasite  • Pulmonary granuloma, focal, chronic, mild, associated with nematode parasite infection  • Perivascular oedema focal acute mild | • Pancreatic duct hyperplasia, diffuse, chronic, marked. | • Pulmonary oedema diffuse acute severe  • Pulmonary congestion unilateral hypostatic  • Pulmonary atelectasis post mortem diffuse | This juvenile female harbour porpoise was euthanized after a six day stay in a rehabilitation centre. The main clinical symptom was severe emaciation upon arrival and weight loss despite a large amount of food intake during her stay. During her final day breathing frequency increased significantly indicating shortness of breath. No chylum was observed in the mesenteric lymph vessels despite digested food being present in the intestines. Total protein level in the serum dropped from 66 to 31 gr/l, which is dramatic. Malabsorption in the intestine thus appears the most likely cause for the continuing weight loss. Malabsorption might have been caused by absence of pancreatic enzymes due to pancreatic duct obstruction. This obstruction is speculatively caused by the formation of hepatic cysts due to congenital ductal plate malformation. Malabsorption led to hypo-proteinemia and as sequel oedema around vessels on the heart and severe oedema in the lungs, which caused shortness of breath. The pneumonia, which was observed, was mild and insignificant as a cause of clinical symptoms. |
| PP060327.1 | • Pneumonia, chronic-active, multifocal, mild  • Lymphadenopathy multifocal subacute to chronic moderate (pulmonary Lnn)  3) Incidental diagnoses:  • Aortic nodule associated with the remains of the ductus of Botallicus | • Pectoral fin traumatic lesion subacute to chronic focal severe | • Pulmonary oedema diffuse acute severe | This juvenile female harbour porpoise in good nutritional condition was found with a partly amputated right pectoral fin. The trauma on the fin had partly healed and the fin was not inflamed. The straightness of the cut and the absence of further trauma on the pectoral fin makes it likely a knife rather than a bite wound caused this trauma. The observed pneumonia was not severe enough to have caused significant clinical problems. The digestive tract was empty possibly because the animal was not capable anymore of hunting after the trauma had occurred. |
| PP060327.2 | • Dermal ulceration multifocal chronic mild  • Dermatitis, suppurative, multifocal, acute, mild. | • Pulmonary empyema multifocal acute moderate  • Smooth muscle hyperplasia, multifocal, chronic, marked, possibly associated with infection with nematodes (probably Torynurus convolutus).  • Pulmonary abscesses, multifocal, chronic, moderate, possibly associated with large and small nematodes (probably Torynurus convolutus, Pseudalius inflexus, and Halocercus invaginatus) and cocci.  • Broncho-interstitial pneumonia, histiocytic, multifocal, chronic, mild, associated with adult and larval small nematodes (probably Halocercus invaginatus).  • Emaciation chronic severe | • Pulmonary oedema diffuse acute severe | This juvenile male harbour porpoise in very poor nutritional condition died after 8 hours in the rehabilitation centre. The most marked lesions were seen in the lungs, and were associated with nematode infections both of the airways and the blood vessels. In addition, at least one pulmonary abscess had evidence of bacterial co-infection. Together, these lesions could have been responsible for the animal’s death. |
| PP060327.3 | • Oesophageal ulcers multifocal acute to chronic moderate  • Gastric ulcer, focal, superficial, chronic, moderate.  • Gastritis, suppurative, superficial, acute, mild.  • Oral ulcer focal acute to subacute moderate  • Lymphadenopathy multifocal acute to chronic moderate  • Pulmonary artery: vasculitis, thrombosing, suppurative, associated with nematode parasites.  • Ocular traumatic lesion focal peracute severe  • Dermal ulcers multifocal chronic mild  • Dermatitis, suppurative, diffuse, acute, moderate.  • Hard palate of mouth: ulcer, focal, acute, mild | • Jaundice diffuse subepidermal fat layer and the aorta moderate  • Hepatitis, necrotizing, multifocal, acute, marked.  • Bile duct hyperplasia, diffuse, chronic, marked.  • Interstitial pneumonia, locally extensive, histiocytic, chronic, moderate, associated with unknown organisms. | • Tracheo-bronchitis, lymphoplasmacytic, suppurative, superficial, diffuse, subacute, marked, associated with food aspiration, cocci, and bacilli. | This adult male harbour porpoise in good nutritional condition died after five days in the rehabilitation centre. The main lesions are found in the lungs the most marked lesions in this animal are a tracheo-bronchitis, interstitial pneumonia, and hepatitis. The cause of the tracheo-bronchitis may be aspiration. The cause of the interstitial pneumonia is not clear: part of the lesions may be due to the round intra-macrophagic organisms, but other etiologic agents may play a role because the extent of the pneumonia is greater than the distribution of the organisms. The cause of the hepatitis is not clear. Also, the importance of the hepatitis is not clear: in one section, the area of affected tissue is large, but in another section, it is not. The anaemia might be the result from blood loss from the stomach and oesophageal ulcers and from the lungs damaged by the parasites. The ocular trauma is caused after the animal has stranded. It is not clear if one aetiology is present for the mouth stomach and oesophageal ulcers, like acid reflux from the forestomach. The fore stomach smell strongly of acetone, which has been noted before with animals with an ulcer associated with parasites. Possibly the caustic effect of acetone is causing the ulcers due to reflux. The aetiology of the biliary tract infection is unclear. Usually parasites can be found in the biliary tracts but this was not so in this animal. The macroscopically observed jaundice does not correspond to the bilirubin values measured in the blood. |
| PP060327.4 | • Hypostatic pulmonary congestion multifocal mild  • Dermatitis focal chronic mild |  | • Pulmonary oedema diffuse acute and severe. | This juvenile male harbour porpoise in good nutritional condition died after 3 days in the rehabilitation centre in the Dolfinarium in Harderwijk. The clinical symptoms indicate gastro-intestinal problems (vomiting, stasis of gastric content) and problems of the respiratory tract (forced breathing, high breathing frequency, exudates during expiration from blowhole). Multiple organ systems might be affected by sepsis or toxic substances from the infection of the respiratory tract might have adverse effects on the digestive tract.  The lack of macroscopic lesions in the lungs is remarkable considering the clinical symptoms. The influence of the opiate administered on respiration has to be considered. No respiratory depression was observed. |
| PP060501 | • Traumatic dermatitis multifocal acute mild  • Subperitonal haemorrhage multifocal acute mild  • Ulcerative keratitis focal subacute moderate  • Pulmonary blood vessel: nematode infection. | • bronchopneumonia, suppurative, locally extensive, acute, moderate, associated with nematode larvae (lungworm larvae). | • Fundic stomach: Gastric epithelial erosion, focal, acute, mild.  • Adrenocortical haemorrhage, multifocal, acute, moderate. | This juvenile female harbour porpoise in good nutritional condition died of an undetermined cause. The lungworm-larvae-associated bronchopneumonia may be the most significant diagnosis. The gastric erosion and adrenocortical haemorrhages are suspected to have occurred peri-mortally. |
| PP060524 | • Bronchopneumonia, granulomatous, multifocal, chronic, moderate, associated with adult and larval nematodes (small lungworms).  • Ulcerative gastritis (cardiac) focal chronic moderate associated with nematode and bacterial infection  • Ulcerative keratitis fibrosing focal subacute to chronic severe  • Endophthalmitis, fibrosing, chronic, marked  • Atrophic bulbi  • Tracheo bronchial and prescapular lymph nodes: lymphadenopathy subacute to chronic moderate.  • Subcutaneous oedema focal acute to subacute moderate  • Pulmonary atelectasis multifocal acute mild  • Subpleural haemorrhages acute diffuse mild  • Pneumonia multifocal chronic mild associated with nematode infection  • Pneumonia focal chronic mild.  • Fundic section of stomach: gastritis, granulomatous, multifocal, chronic, mild, probably associated with parasite infection  • Dermal lesions acute and chronic multi focal mild  • Mesenteric lymph node: lymphadenitis, granulomatous, multifocal, chronic, mild, probably associated with parasitic infection. | • Pachymeningitis, necrotizing, suppurative, locally extensive, acute, marked, associated with fungal hyphae (Aspergillus sp.).  • Cerebellum: panencephalitis, pyogranulomatous, haemorrhagic, necrotizing, locally extensive, associated with fungal hyphae (Aspergillus sp.) and mixed bacterial infection.  • Otitis media purulent subacute to chronic diffuse severe  • Pia mater of cerebellum: leptomeningitis, suppurative, acute, mild  • Pia mater of cerebrum: leptomeningitis, lymphocytic, subacute, mild | • Pulmonary oedema diffuse acute severe | This juvenile female harbour porpoise in good nutritional condition died 9 hours after being admitted into the rehabilitation centre. The main lesions were a severe panencephalitis, pachymeningitis and otitis media caused by Aspergillus sp. infection combined with a mixed bacterial infection. This resulted in marked inflammation and necrosis of the cerebellum and overlying meninges. The cause is unclear, but the presence of multiple pathogens suggests direct introduction from the external environment, e.g., via a perforated eye. The Clostridium perfringens which was cultured form the middle ear and the skull lesion might have been successful in the live animal due to the anaerobic conditions created by the tissue damage and necrosis caused by the other invaders. |
| PP061122.1 | • Pulmonary thrombosis, associated with infection with nematode worms (likely Pseudalius inflexus)  • Spleen, lymph nodes: increased numbers of hemosiderin-laden macrophages  • Dermatitis, suppurative, hyperplastic, chronic, multifocal, mild, associated with Candida sp  • Dermal lesions multifocal acute mild  • Myositis, lymphocytic, subacute, mild  • Corneal oedema diffuse acute mild  • Parasitic infestation multifocal chronic mild to moderate | • Bronchopneumonia, pyogranulomatous, necrotizing, chronic, moderate, associated with Aspergillus sp. Infection  • Bronchopneumonia, pyogranulomatous, multifocal, chronic, mild to moderate, associated with lungworm (likely Stenurus minor) infection  • Emaciation diffuse chronic severe | • Pulmonary oedema diffuse peracute severe  • Gastric impaction acute severe.  • Sepsis | This female juvenile harbour porpoise in poor nutritional condition was euthanized. The most significant lesion appears to be the pyogranulomatous bronchopneumonia, associated both with S. minor larvae and Aspergillus sp. infection.  The gastric impaction is known to occur in debilitated animals and might be a consequence of malfunctioning of the GI tract due to emaciation or sepsis. Enterococcus faecalis sepsis was demonstrated by bacteriological examination. The organism was cultured exclusively out of the lung, spleen, liver, adrenal, and uterus. A mixed culture was found in the kidneys and the lung associated Ln (together with Enterococcus faecalis). |
| PP061122.2 | • Bronchopneumonia, granulomatous, multifocal, chronic, mild, associated with nematode infection (probably Stenurus minor).  • Dermatitis multifocal purulent acute mild  • Dermal lesion chronic focal mild  • Parasitic infestation multifocal chronic mild to moderate  • Oesophageal ulcer, focal, acute, mild  • Pancreatic duct hyperplasia, mild | • Sepsis secondary to pneumonia  • Bronchopneumonia, suppurative, multifocal, subacute to chronic, severe, associated with *Escherichia coli* infection  • Myositis, suppurative, multifocal, acute, moderate (consistent with *Escherichia coli* infection).  • Fasciitis, suppurative, focal, acute, moderate (consistent with *Escherichia coli* infection).  • Pulmonary thrombosis, organizing, multifocal, chronic, moderate, with nematode (likely Pseudalius inflexus) and bacterial (likely *Escherichia coli*) infections. |  | This male juvenile harbour porpoise in poor body condition died in the rehabilitation centre. The most significant lesion was the severe pneumonia. The observed sepsis will have come from the pneumonia. Abscessation might explain the resilience of the E. coli to amikacin treatment. The E. coli also infected the nematode-induced pulmonary thrombi, likely resulting in septic emboli that spread to other parts of the body. The use of prednisolone can have facilitated the occurrence of sepsis. |
| PP061123.1 | • Hepatic congestion acute diffuse moderate | • Pulmonary oedema, diffuse, acute, marked.  • Renal tubular necrosis, multifocal, acute, moderate, associated with hyaline granular casts (myoglobin?). |  | This juvenile female harbour porpoise in good nutritional condition died during transport. The most significant finding was the acute pulmonary oedema. Together with the suspected myoglobin casts and tubular necrosis in the kidney this speculatively points to capture myopathy leading to heart failure and death. |
| PP070221 | • Cholangiohepatitis, diffuse, fibrosing, chronic, marked, associated with trematode infection, (Campula oblongata)  • Dermatitis, focal, suppurative, acute, mild, with suspect poxvirus inclusions.  • Skin cut, chronic, with focal suppurative dermatitis and focal fibroplasia (physical trauma).  • Dermatitis, suppurative, focal, acute, associated with bacterial infection and cestode infection.  • Pulmonary haemorrhage focal subacute moderate.  • Pulmonary oedema diffuse acute mild  • Pyloric stomach: gastritis, focal, fibrosing, ulcerative, chronic, moderate, associated with trematode infection (Pholeter gastrophilus).  • Bronchus: lungworm infection (probably Halocercus invaginatus and Stenurus minor)  • Pulmonary artery: arteritis, fibrosing, diffuse, chronic, mild to severe, associated with nematode infection (Torynurus convolutus).  • Pleural oedema focal acute mild  • Emphysema, multifocal, mild  • Splenic lymphoid hyperplasia, moderate | • Bronchopneumonia multifocal, lymphocytic and eosinophilic, chronic, moderate, associated with nematode infection., (probably Halocercus invaginatus and Stenurus minor)  • Bronchopneumonia multifocal, suppurative, acute, moderate, associated with bacterial infection. |  | This juvenile male harbour porpoise in poor nutritional condition died 20 minutes after his arrival in the rehabilitation centre. The primary diagnosis was pneumonia. The pneumonia had a chronic component associated with nematode infection and an acute component associated with a bacterial infection. Possibly the parasitic infection may predispose for a bacterial infection. The partial occlusion of the pulmonary arteries by nematodes might be more significant then hitherto assumed in disturbing the perfusion ventilation relation of the lung tissue and needs further examination in live animals. |
| PP070317 | • Bronchi: nematode infection (probably Halocercus invaginatus) diffuse chronic marked  • Pneumonia, pyogranulomatous, multifocal, chronic, mild  • Pulmonary oedema, diffuse, acute, mild.  • Interstitial pneumonia, granulomatous, focal, chronic, mild  • Parasitic infection of the alveoli (probably Stenurus minor).  • Bronchopneumonia, lymphoplasmacytic, diffuse, chronic, mild, associated with bacterial infection (probably Pseudomonas aeruginosa)  • Pulmonary abscess focal chronic mild associated with trematode (?) infection  • Haemorrhages, subpleural, pulmonary and costal, multifocal, acute, mild.  • Cholangitis, proliferative, chronic, marked, associated with Campula oblonga infection.  • Pulmonary artery: nematode infection (probably Torynurus convolutus)  • Thyroid congestion, diffuse, acute, marked. | • Hepatic necrosis, periacinar, extensive, acute, marked associated with a moderate icterus  • Hepatic lipidosis, diffuse, acute, moderate |  | This adult female gravid harbour porpoise was in good nutritional condition and freshly dead upon necropsy. The death of this animal is most likely related to the severe hepatic necrosis and lipidosis, which is associated with the generalized icterus (visible most clearly by the yellow colour of the intima of the aorta and pulmonary trunk). The liver disease fits with the results of clinical chemical analysis, which show strongly increased blood concentrations of liver enzymes (ASAT, ALAT, LD). The cause of the hepatic necrosis is not clear. It has a resemblance to fatty liver syndrome in cats and equine hyperlipemia in horses. Both diseases are conditions in fat animals that suffer some kind of stress, and animals dying from this disease develop severe hepatic lipidosis. In horses, pregnant or lactating mares are predisposed. In both cats and horses the pathogenesis is obscure. Histological evaluation of the liver may help to make a more precise diagnosis.  Severe leukopenia was observed before the rise of liver enzymes. The leukopenia is due to a marked inflammatory reaction of which the location remains obscure. Toxins from the inflammation site in combination with the existing moderate lipidosis are likely to have caused the hepatic necrosis, which ultimately caused the death of the harbour porpoise.  Although the large lungworms in this porpoise occluded a large part of the primary bronchial lumina and would likely affected have affected its endurance, I doubt that this infection played an important role in its death, since the infection was chronic and the porpoise was in good nutritional condition. The small nodules in the lung were likely remnants of small lungworms and of little consequence. The pleural haemorrhages likely occurred just before death, perhaps due to excessive respiratory movements. The trematode infection of the bile ducts was chronic and the associated lesions were mild. No clear lesions were seen in association with the heartworm infection.  This adult female was gravid with a 60-cm-long male foetus. Foetus, uterus, and placenta appeared normal. |
| PP070328 | • tracheo-bronchitis, lymphoplasmacytic, erosive, diffuse, chronic, mild.  • lymphonodular hyperplasia (tracheo bronchial lymph node)  • vasoconstriction and peri-arteritis, eosinophilic, acute, marked (mesenteric artery)  • skin lesions, multifocal, acute, moderate  • pulmonary oedema focal acute mild  • bronchitis multifocal acute to subacute mild associated with parasitical infestation  • oral ulci multifocal acute mild  • cholangitis, fibrosing, chronic, diffuse, mild.  • gastritis, eosinophilic, superficial, subacute, mild. (pyloric stomach)  • granulomatous gastritis focal chronic mild associated with parasites (pyloric stomach)  • adrenocortical necrosis, multifocal, peracute, moderate.  • venous thrombosis, local, chronic (colonic serosal vein)  • Thyroid necrosis, diffuse, peracute, marked | • Bronchopneumonia caused by bacterial infection of the lungs  • bronchopneumonia necrotizing, suppurative, diffuse, acute, moderate, associated with bacterial infection (mixed coliforms). |  | This male juvenile harbour porpoise in moderate body condition died during transport to the rehabilitation centre after having been trapped in a gillnet for over 30 minutes. The proximal cause of death is likely the bronchopneumonia caused by bacterial infection of the lungs. The most likely source of these bacteria is aspirated contents of the digestive tract.  No signs of aspiration of seawater were found.  Histological examination of the thyroid gland gave no indication of malfunctioning. The question why this animal was so small remains unanswered. |
| PP080403 | • Lung: nematode infection of the alveoli, mild.  • Pulmonary blood vessel: nematode infection, mild.  • Lymphadenitis (pulmonary) multifocal moderate acute  • Proximal intestine: enteritis, eosinophilic, focal, subacute, mild. | • Dermal trauma (blowhole) multifocal acute severe  • Lung (left cranioventral lobe): bronchopneumonia, suppurative, locally extensive, acute, marked. | • Pulmonary oedema diffuse moderate acute | This juvenile female harbour porpoise was found alive in moderate nutritional condition. Severe trauma was inflicted to the blowhole and underneath the right eye and involving the eyelids of both eyes. Due to the severity of the trauma whereby the animal was unable to close the blowhole, the harbour porpoise was killed with a toxic injection. The most significant diagnosis was an acute focal pneumonia and a mild infection of the pulmonary blood vessels. However, these diagnoses seem unlikely to have caused the emaciation of the harbour porpoise. By default, it must be concluded that the head injury made the animal unfit to feed and hence its nutritional condition declined. |
| PP080701 | • Lymphadenitis multifocal haemorrhagic acute moderate  • Splenitis diffuse acute moderate  • Liver: bile duct hyperplasia, diffuse, marked.  • Sero hemo pericard diffuse acute mild  • Pancreatic duct hyperplasia, focal, moderate.  • Adrenocortical haemorrhage and necrosis, multifocal, acute.  • Pneumonia multifocal chronic mild associated with nematodes  • Hepatitis focal chronic (to healed) mild | • Interstitial pneumonia, haemorrhagic, diffuse, acute, marked, associated with Staphylococcus aureus infection.  • Pleuritis bilateral focally extensive fibrinopurulent acute moderate.  • Pneumonia, lymphohistiocytic, locally extensive, chronic, marked.  • Pancreatic angitis, suppurative, necrotizing, chronic-active, multifocal, marked. | • Sepsis  • Pulmonary oedema diffuses acute moderate | This juvenile female harbour porpoise died of an acute haemorrhagic pleuro pneumonia which turned into a sepsis. The hepatitis was chronic. The aetiology is unclear as is the significance of this hepatitis to the health of the animal. Likewise, is the aetiology and significance of the sero hemo pericard unclear. The location of the infection in the medial rostral thirds of the lung indicate a bacterial infection introduced by inhaling the infectious agent. The fairly marked infestation of the pulmonary blood vessels with nematodes has an unclear significance. Together, this porpoise had a chronic problem of liver and pancreas (cause remains to be determined: check blood for bile acids) and an acute problem of the lung: S. aureus infection with spread to other organs. In addition, there was a more chronic problem of the lung, which was negative for mycobacteria by ZN stain or other pathogens by Grocott and Gram stains. |
| PP081222 | • Epiglottis: epiglottal ulcer, focal, acute, mild.  • Teeth: Attrition, generalized, marked.  • Pharynx: pharyngitis, necrotizing, multifocal, acute, mild.  • Oesophagus: oesophagitis, necrotizing/ulcerative, multifocal, acute, mild.  • Forestomach: parasite infection.  • Bile ducts: cholangitis, fibrosing, diffuse, chronic, moderate.  • Heart and pulmonary trunk: parasite infection.  • Skin: ulceration and scars, multifocal, chronic, mild. | • Lungs: bronchopneumonia, suppurative, multifocal or coalescing, chronic-active, marked, associated with parasite infection (and bacterial infection?)  • General: emaciation, marked. |  | The most significant lesion was the bronchopneumonia, likely with both a parasitic and bacterial component. |
| PP090626 | • Tracheo-bronchitis, lymphoplasmacytic, diffuse, superficial, chronic, mild.  • Bronchopneumonia, histiocytic, diffuse, chronic, mild, associated with nematode infection.  . • Metritis, lymphocytic, diffuse, superficial, chronic, mild.  • Lung associated lymph node, intestinal lymph node: lymphadenitis, eosinophilic, diffuse, acute, mild.  • Cholangitis multifocal chronic mild associated with parasitic infection  • Galactophoritis, lymphoplasmacytic, superficial, chronic, mild.  • Pulmonary arteriitis multifocal mild associated with parasitic infection  • Dermatitis multifocal acute mild | • Hepatic lipidosis, moderate diffuse.  • Hepatitis, necrotizing, suppurative, focal, acute, mild.  • Bronchiolitis obliterans, multifocal, subacute, moderate |  | This adult female in good nutritional condition died within hours of being found on the beach. The cause of death is unknown. Brucella ceti was cultured form lung tissue, pulmonary lymph node and spleen. Possibly the cow had aborted previously although the time of stranding coincides with the normal calving season. Taking all results together, the most significant lesion is the hepatic lipidosis, which is associated in some areas (but too little liver tissue examined histologically until now) with hepatocyte necrosis and acute inflammation. This is reminiscent of acute ketosis of lactating cows, or pregnancy toxaemia of sheep. However, the pathologic changes in the liver are not convincing and more liver samples from different parts of the liver need to be examined histologically.  Another lesion that might be significant is the bronchiolitis. In about 2/3 of the observed bronchioles in the left and right lung, the lumen is obstructed either by the presence of inflammatory cells or by contraction of the smooth muscle. It is not clear how much effect this would have on respiratory function, but it is likely that it would decrease it. The cause of the bronchiolitis is most likely from nematode infection. These were found at one location in the alveolar lumina, although none were observed in the bronchioles in the tissues examined microscopically. |
| PP100105 | • Oesophageal ulcers multifocal acute mild  • Corneal ulcer focal chronic mild  • Pyloric stomach: gastritis focal chronic mild associated with a fungal (yeast) infection and the presence of nematodes  • Middle ear: otitis diffuse chronic mild associated with the presence of nematodes  • Hepatic lipidosis diffuse acute mild  • Oesophagus, fundic and pyloric stomachs: yeast-like infection.  • Dermal lesions multifocal acute mild likely due to trauma related to stranding  • Dermatitis multifocal chronic mild  • Lung-associated lymph node: benign lymphoid hyperplasia, moderate.  • Pulmonary blood vessel: nematode infection.  • Liver: hepatic lipidosis, diffuse, mild.  • Intestine: enteritis, ulcerative, focal, acute, mild.  • Skeletal muscle: muscle degeneration, multifocal, acute, moderate.  • Skeletal muscle: muscle atrophy, diffuse, chronic, moderate.  • Pancreas: ductular hyperplasia, chronic, moderate |  | • bronchopneumonia suppurative, granulomatous, diffuse, chronic-active, associated with mixed bacterial and yeast-like (?) infection and aspiration of food remains marked. | This juvenile male harbour porpoise with a very poor nutritional condition was euthanized after 48 hours in the rehabilitation centre. The main lesions found were broncho-pneumonia besides emaciation. The respiratory organs showed three different types of lesions. First small calcified nodules which will not have had any significance (probably lungworms). Second major areas of lung which were consolidated and third large parts of lung which had a different colour and was slightly firmer (both likely aspiration pneumonia). The wasting of the back muscles in the presence of a relatively thick blubber layer is likely due to the animal preferentially fulfilling its calorie needs form muscle digestion rather than to threaten its insulation by digesting its isolating layer of blubber. The significance of the pancreatic duct hyperplasia is unclear. The proximal cause of death (or reason to euthanise) is the aspiration bronchopneumonia. The ultimate reason which will have caused the emaciation is unclear, possibly a mycotic infection of the gastro intestinal tract or maldigestion due to pancreatic malfunction. |
| PP100928.1 | • Dermal incision wounds multifocal acute mild  • Pulmonary hypostatic congestion diffuse mild |  | • Pulmonary oedema, diffuse, acute, moderate. | This male neonatal harbour porpoise in poor level of nutrition was euthanized as no artificial milk formulas are known that can maintain neonatal harbour porpoises. The poor level of nutrition and empty intestines indicate the animal had not been feeding recently. The observed stomach ulcers may have been caused by the associated stress and malnutrition. The incision wounds in the skin may have occurred due to contact with the ground during the stranding process. |
| PP100928.2 | • Oesophagus: oesophageal ulceration, multifocal, acute, mild.  • Fundic stomach lesions unknown aetiology |  | • Lung: pulmonary oedema, diffuse, acute, moderate. (or artefact) | This female neonatal harbour porpoise in poor body condition was euthanized as no artificial milk formulas are known that can maintain neonatal harbour porpoises. The longitudinal ulcers in the oesophagus may have been caused by damage during tube feeding. The lesions in the fundic stomach are of unknown aetiology and significance. |
| PP110228 | • Renal tubular epithelial degeneration, multifocal, acute, mild, associated with protein casts (myoglobinuria).  • Alveolitis, suppurative, diffuse, acute, mild, associated with microthrombi.  • Tracheo-bronchial lymph node: lymphoid hyperplasia, moderate.  • Hepatic congestion diffuse chronic mild  • Renal pelvic haemorrhage focal acute mild  • Pulmonary nematode infection, very mild.  • Hepatic lipidosis, diffuse, mild.  • Splenic lymphoid hyperplasia, mild.  • Pyloric stomach: gastric haemorrhage, focal, acute, mild | • Oesophagitis ulcerative multifocal to confluescent acute marked | • Pulmonary oedema diffuse peracute severe | This juvenile male harbour porpoise in moderate nutritional condition was euthanized after 5 days in the rehabilitation centre. The most important diagnosis was severe ulcerative inflammation of the oesophagus and a severe gastritis in the pyloric stomach. The porpoise had a fatty liver which may indicate an abnormal metabolism of fatty acids possibly caused by “partial” fasting. The importance of the pulmonary lesion is unclear, lung associated lymph nodes were enlarged but possibly these lymph nodes drain the oesophagus as well (unknown to me for this species). Based on the epithelial damage of the digestive tract at multiple site a viral aetiology has to be considered. |
| PP110329 | • Oesophagus: oesophagitis, granulomatous, focal, chronic, mild, associated with migrating parasite.  • Fundic stomach, pyloric stomach: vasculitis, granulomatous, multifocal, chronic, moderate, associated with nematode infection  • Pyloric stomach: Focal gastritis, ulcerative, granulomatous, locally extensive, chronic, marked, associated with parasite infection (Pholeter gastrophilus?).  • Dermatitis, erosive, suppurative, chronic-active, moderate, associated with bacteria and yeast-like organisms  • Lung-associated lymph node, spleen, prescapular lymph node: lymphoid hyperplasia, benign, moderate.  • Diffuse adrenocortical hyperplasia chronic severe  3) Incidental diagnoses:  • Pulmonary artery: arteritis, lymphocytic, diffuse, chronic, moderate, associated with nematode infection. | • Bronchopneumonia multifocal, chronic, moderate, associated with nematode infection.  • Bronchopneumonia multifocal, acute, marked, likely associated with bacterial infection.  • Focal ulcerative chronic gastritis severe (fore stoma |  | This adult female harbour porpoise in very poor nutritional body condition died during transport with symptoms of respiratory distress. Probably the most significant lesion is the mixed bacterial and parasitic bronchopneumonia. It is not clear whether the bacterium Actinobacillus delphinicola contributed to this inflammation, since the association between this newly discovered bacterium (in 1996) and disease is not known.  The second most important lesion is a very large stomach ulcer in the fore stomach. |
| PP110711 | • Splenomegaly diffuse acute mild (lymphoid hyperplasia, mild)  • Pulmonary arterial parasitic infection multifocal chronic moderate  • Bronchio-tracheal parasitic infection multifocal chronic moderate  • Middle ear parasitic infection diffuse chronic moderate  • Dermal trauma multifocal acute to subacute mild  • Oesophageal ulceration multifocal chronic mild | • pneumonia, pyogranulomatous, locally extensive, chronic, marked.  • Emaciation diffuse chronic severe  • Lymphadenitis multifocal subacute to chronic marked (Tracheo-bronchial lymph node, prescapular lymph node: lymphoid hyperplasia, marked.)  • Hepatocytic degeneration, random, multifocal, acute, moderate, with neutrophil infiltration and hepatocytic necrosis | • Pulmonary oedema acute severe | This juvenile female harbour porpoise in poor nutritional condition died after being admitted less than 24 hours in the rehabilitation centre. The main pathological findings were a very poor nutritional condition, a chronic pneumonia, in combination with splenomegaly, multiple hyperplastic lymph nodes and acute hepatocytic degeneration. It might be speculated that the animal had been unable to forage effectively, possibly due to the effects of the focal pneumonia, which caused a decrease in immuno-resistance, which resulted in the local pneumonia turning into sepsis with hepatocytic degeneration due to toxin release or bacterial leakage from the intestines. Brucella ceti is the most likely etiologic agent. Aspergillus was cultured from a lung sample but not found on histology. It is not clear how this juvenile animal (1 or 2 years old) contracted a Brucella ceti infection |
| PP110928.1 | • Dermal trauma focal acute mild  • Corpus alienum (sand) in the air-sacs multifocal acute moderate  • Fundic stomach: gastric erosion, focal, superficial, acute, mild | • Kidney: renal tubular epithelial cell vacuolation, diffuse, marked.  • Liver: hepatocytic vacuolation, diffuse, marked. | • Pulmonary atelectasis multifocal acute (or possibly post mortal due to resorption)  • Lung: pulmonary oedema, diffuse, acute, moderate. | This neonatal female harbour porpoise in moderate level of nutrition was euthanized after 2 days in rehabilitation. The main finding on necropsy were vacuolization of hepatocytes and tubular epithelium of the kidneys. These lesions are in accordance with the signs noted which were strong defence musculaire on admission, electrolyte imbalance and the continuous vomiting of administered fish gruel. The tubular epithelial vacuolation in the kidneys and hepatocytic vacuolation in the liver resembles a lysosomal storage disease, like beta-mannosidosis in cattle, or an intoxication, like Swainsona intoxication. Glycogen storage disease type 1 (von Gierke disease) specifically affects liver and kidney (Vet Pathol. 1995 Sep;32(5):460-5. |
| PP110928.2 | • Lung: alveolitis, pyogranulomatous, focal, chronic, mild, associated with nematode infection.  • Pulmonary artery: nematode infection, mild.  • Bronchus: nematode infection, moderate.  • Pulmonary-associated lymph node: benign lymphoid hyperplasia, mild.  • Liver: bile duct hyperplasia, focal, chronic, marked. | • Dermal trauma acute multifocal to coalescing marked | • pulmonary oedema, diffuse, acute, marked. | This male juvenile harbour porpoise in good nutritional condition died during transport to the rehab centre. The proximate cause of death was the pulmonary oedema, which may have been caused by psychogenic shock due to stress and pain. The animal was severely wounded by predators. It is unclear whether this happened at sea (e.g. by grey seals) which would then cause stranding or post stranding by birds and terrestrial scavengers (foxes and dogs). The observations on the respiratory tract do not lead to clear conclusions on the pathology observed. There could either be an insignificant old focal pneumonia or possibly an acute pneumonia which may have been a reason for the animal to strand. Histology will give further clues. The observed bite wound on the right tail fluke is interesting. This bite wound may well be the result of an attack by another cetacean. |
| PP111219 | • Dermal lesion chronic mild  • Lung: nematode infection of pulmonary vein, mild.  • Tracheo-bronchial lymph node: benign lymphoid hyperplasia, mild.  • Pancreas: pancreatic collecting duct hyperplasia, diffuse, chronic, mild. | • Bronchopneumonia, haemorrhagic, suppurative, diffuse, acute, marked, associated with bacterial infection  • Lung: alveolitis, pyogranulomatous or granulomatous, multifocal, chronic, mild or marked, associated with nematode infection. |  | This juvenile male harbour porpoise in moderate to poor nutritional condition died after five days in the rehabilitation centre. The main finding at necropsy was the pneumonia which had a chronic component strongly associated with a nematode infection and an acute component which was purulent and not associated with a nematode infection. The acute bacterial bronchopneumonia was responsible for the death of the animal. Location (ventral parts of the lung), the observation of several different species of bacteria and the history with tubing and frequent vomiting suggest the possibility of an aspiration pneumonia, although no food remains were seen on histology. |
| PP120703 | • Dermal and subdermal trauma multifocal acute moderate  • Gastritis multifocal acute to subacute mild.  • Lung: bronchiolitis, suppurative, diffuse, acute, mild.  • Eyelid: conjunctivitis, suppurative, superficial, acute, mild. |  | • Pulmonary oedema diffuse acute moderate. | This neonatal harbour porpoise in moderate nutritional condition was euthanized. The main findings were an absence of milk or nutritional content in the stomach and bowels and presence of sand throughout the digestive tract. The presence of sand throughout the digestive tract and the presence of multiple small erosions in the fore stomach indicate this animal had been ingesting sand previous to its stranding. Lack of nutrition and consequent hunger may have caused this animal to ingest sand; possibly separation of its mother is the ultimate cause of the animal’s stranding. |
| PP120906.1 | • Lung: nematode infection in alveoli.  • Bronchitis, multifocal, lymphocytic, chronic, associated with nematode infection.  • Verminous arteritis multifocal acute to chronic mild (pulmonary arteries)  3) Incidental diagnoses:  • Dermal trauma multifocal acute to subacute moderate  • Stomatitis, ulcerative, focal, acute, mild.  • Oesophageal ulcers multifocal acute to chronic mild  • Benign lymphoid hyperplasia moderate  • Verminous otitis diffuse chronic moderate  • Pyloric stomach: granuloma, eosinophilic, focal, chronic, mild. | • Pneumonia, pyogranulomatous, multifocal to coalescing, chronic, marked, associated with fungal (Aspergillus fumigatus) infection.  • Encephalitis, pyogranulomatous, multifocal, chronic, moderate, associated with fungal (Aspergillus fumigatus) infection.  • Pulmonary haemorrhage and oedema diffuse acute marked |  | This male juvenile harbour porpoise in moderate nutritional condition died within one day at the rehabilitation centre. The most severe lesion by far is the pyogranulomatous pneumonia associated with Aspergillus fumigatus infection. Interestingly, the fungus has spread to the cerebellum, but is not present in sections of any other tissues. The pneumonia was severe enough to be responsible for the death of the animal. |
| PP120906.2 | • Lung: foreign body (trematode egg).  • Lung-associated lymph node: venous nematode infection.  • Subcutaneous emphysema diffuse acute moderate (possible artefact)  • Dermatitis multifocal acute and chronic and healed moderate  • Benign lymphoid hyperplasia multifocal acute to chronic moderate  • Cholangitis, hyperplastic, multifocal, chronic, moderate, associated with trematode (Campula oblonga) infection.  • Cutaneous papilloma’s, multifocal, chronic, moderate.  • Verminous otitis media diffuse chronic mild  • Pyloric stomach: gastritis, fibrosing, multifocal, chronic, mild, associated with trematode (Pholeter gastrophilus) infection.  • Renal calcification multifocal chronic mild  • Lingual ulceration focal acute mild | • sepsis  • Pneumonia, pyogranulomatous, multifocal, chronic, moderate, associated with nematode infection.  • Interstitial pneumonia, suppurative, diffuse, acute, moderate.  • Joint capsule left jaw: arthritis, suppurative, diffuse, acute, marked, associated with coccoid bacteria.  • Hepatic abscesses, multiple, marked. | • Pulmonary oedema acute diffuse marked | COMMENTS: This juvenile female harbour porpoise in moderate to poor nutritional condition died spontaneously during transport. The suppurative arthritis and the coccoid bacteria arranged in strings within the exudate fits with Streptococcus dysgalactiae infection. It is difficult to determine where this S. dysgalactiae infection started: mandibular joint, lung (secondary to lungworm infection), or liver (secondary to liver fluke infection). The diffuse presence of neutrophils in the alveolar walls (suppurative interstitial pneumonia) suggests that the bacterium has spread to the blood (sepsis), which would be the most likely proximate cause of death. Interestingly, the mammary gland had no evidence of S. dysgalactiae infection, even though this bacterium is a well-known cause of mastitis in cattle.  The arthritis of the mandible may well have hindered the animal in hunting and feeding and be as such a more ultimate cause of death. |
| PP120906.3 | • Adrenalitis, necrotizing, multifocal, chronic, with eosinophilic intranuclear inclusions.  • Hemoperitoneum acute diffuse moderate (if not artefact)  3) Incidental diagnoses:  • Cholangitis, lymphocytic, diffuse, chronic, marked, with marked periductular fibrosis, associated with trematode (Campula oblonga) infection.  • Lip: cutaneous ulcer, superficial, focal, acute, mild.  • Blowhole ulcer: epidermal necrosis, focal, acute, mild, associated with eosinophilic intranuclear inclusions.  • Pyloric stomach: gastritis, granulomatous, focal, chronic, mild.  • Skin fin: incision wound, superficial, chronic, mild. | • Bronchopneumonia, granulomatous, multifocal, chronic, moderate, associated with nematode infection.  • Interstitial pneumonia, suppurative, histiocytic, locally extensive, chronic, moderate.  • Cerebrum: polioencephalitis, multifocal, mild.  • Hepatitis, necrotizing, multifocal, acute, marked. | • Pulmonary oedema acute diffuse marked | This male juvenile harbour porpoise in moderate nutritional condition was euthanized after 8 days in the rehabilitation centre. Clinical symptoms indicate a peracute severe infection, possibly sepsis (hemoperitoneum) pneumonia (focal pneumonia) or an infection of the CNS (no gross pathological indications). Based on the presence of eosinophilic intranuclear inclusions in the adrenal gland and in the blowhole ulcer, there is evidence of a systemic herpesvirus infection. This would fit with the neuronophagia suspected in the cerebrum, as well as the necrotizing hepatitis. These pathological changes fit with the aberrant behaviour observed in this animal during rehabilitation. |
| PP121031 | . • Bronchitis, lymphoplasmacytic, diffuse, chronic, mild.  • Verminous arteriitis multifocal acute to chronic moderate  • Benign lymphoid hyperplasia multifocal acute moderate  • Acetonemia  • Cholangitis, fibrosing, hyperplastic, diffuse, chronic, moderate, associated with Campula oblonga infection.  • Renal cyst.  • Thymic cysts.  • Aorta: atheroma (?), mild.  • Mesenteric lymph node: lymphadenitis, eosinophilic, focal, acute, mild.  • Prescapular lymph node: benign lymphoid hyperplasia.  • Fore stomach: scar, superficial, focal.  • Epidermal and dermal scars, multiple, mild | • Bronchopneumonia, pyogranulomatous, necrotizing, multifocal, chronic, moderate, with suspect eosinophilic intranuclear inclusions, associated with Salmonella sp. infection and nematode infection. | • Pulmonary oedema, diffuse, acute, moderate | This gravid adult female in moderate body nutritional condition died shortly after starting the transport to the rehabilitation centre. The main lesions were a diffuse acute bronchopneumonia associated with Salmonella sp. and a moderate to severe infestation of the pulmonary arteries with nematodes. The eosinophilic intranuclear inclusions in the epithelial cells of the bronchus are suspect of herpesvirus infection. The acetonemia, empty stomach and slightly wasted musculature indicate she had not been feeding for quite a while. Likely she did not have the necessary fitness to hunt caused by either a lack of oxygen due to the pneumonia and ill feeling caused by the toxins of infection or by the disturbed circulation due to the infestation of the pulmonary arteries with nematodes. Her proximate cause of death is unclear. |
| PP121102 | • Bronchopneumonia, lympho-plasmacytic, diffuse, chronic, associated with nematode infection.  • Lung-associated lymph node, prescapular lymph node: benign lymphoid hyperplasia  • Dermal scars multifocal chronic mild  • Dermal wound multifocal acute mild  • Subdermal scar focal chronic mild  • Cholangitis, fibrosing, diffuse, marked, associated with Campula oblonga infection.  • Ascites very mild (normal?)  • Hydropericardium very mild  • Hydrothorax very mild | • Pneumonia, granulomatous, coalescing, chronic, marked, associated (in some foci) with fungal structures. |  | This adult male harbour porpoise in good to moderate nutritional condition died after three days in rehabilitation. The main finding was a severe pneumonia associated with Aspergillus fumigatus and associated hyperplasia or inflammation of the draining lymph nodes. Scars on the tail stock indicate this animal was once caught in a line which tightened around the end of its tail stock. An associated hard swelling might indicate bony involvement. The proximate cause of death is most likely the pneumonia and associated phenomena. (toxins, laboured breathing, hypoxia). |
| PP121130 | • Pulmonary hyperaemia acute to chronic multifocal mild and associated with pneumonia  • Pulmonary arteritis multifocal chronic mild associated with verminous infection.  • Benign lymphoid hyperplasia multifocal chronic moderate  3) Incidental diagnoses:  • Pulmonary oedema peracute diffuse mild  • Oesophageal ulceration multifocal chronic mild  • Hydropericardium diffuse mild  • Dermal trauma multifocal chronic mild possible rake or bite marks(?). | • Asphyxia due to pneumonia in combination with hydrops ascites.  • Bronchopneumonia pyogranulomatous, locally extensive, chronic, moderate, associated with large adult nematodes (Torynurus convolutus, Halocercus invaginatus) and small nematode larvae (Stenurus minor).  • Forestomach, jejunum, colon: vasculitis, leukoclastic, haemorrhagic, multifocal, chronic-active, marked  • Ascites diffuse severe.  • Uterus: vasculitis, leukocytoclastic, haemorrhagic, multifocal, subacute, mild.  • Dermatitis, pyogranulomatous, multifocal, chronic, marked, with epidermal hyperplasia, keratin pearls, and bacterial infection. |  | This juvenile female harbour porpoise was emaciated and died after 17 days in the rehabilitation centre. Unusual lesions in this porpoise are those of leukocytoclastic vasculitis of the small and medium-sized arteries of the stomach, intestine, and (to a much milder degree), uterus. I have never seen vascular lesions like this in harbour porpoises. Since the lesions extended to the lumen of the intestine, they would have resulted in blood loss into the intestinal lumen, and thus fit with the anaemia diagnosed clinically. Since the lesions also extended to the serosal surface, they also fit with the mild hydrops ascites observed at gross autopsy.  The protrusion of the teats and clitoris and vulvar mucosa from the genital slit were caused by pressure from the ascites. The combination of anaemia, pressure on the thorax form the distended abdomen and the pulmonary infection, including the verminous infection of the pulmonary arteries are likely to have compromised the breathing, circulation and oxygen supply of the animal and this is probably the proximate cause of its death.  The skin lesions, with apparent invagination of the epidermis, resembles an inverted papilloma. There appears to be an associated increase in vascularization of the subjacent dermis. It seems to fit well with papillomavirus infection, and needs to be followed up. The bacterial infection and subsequent inflammation seem to be secondary. |
| PP130204 | • Dermal lesions multifocal chronic mild  • Dermal lesions multifocal acute mild  • Lingual ulcer focal acute mild  • Liver: bile duct hyperplasia, diffuse, chronic, mild.  • Brain: medulla oblongata, malformation.  • Urinary bladder: serosal haemorrhage, multifocal, acute, mild.  • Pyloric stomach: gastric ulceration, focal, acute, mild, with subjacent haemorrhage.  • Oesophagus: oesophageal ulceration, multifocal, acute, mild.  • Corneal perforation acute focal marked | • Skeletal muscle: muscle degeneration, locally extensive, acute to subacute, marked.  • Kidney: urolithiasis, mild.  • Right heart: myocardial oedema, multifocal, acute, mild. | • Pulmonary oedema, diffuse, acute, mild. | This juvenile male harbour porpoise in in moderate level of nutrition and fresh state of autolysis as humanely killed as there was an inability to administer sufficient calories and the animal continued to lose weight. Significant findings were subperitoneal haemorrhages on and close to the urinary bladder and pale muscles suggesting necrosis. Histology confirmed pathology of the muscles. However, the muscles were not necrotic. Blood values indicated kidney failure (increasing urea up to 30(20 is high normal) and creatinine from 19 to 80 and Na from 144 to 165), which probably caused the vomiting and may also have caused protein loss and wasting despite the food intake. The proteinaceous material observed in the distal renal tubuli may therefore be more significant then they appeared. Speculatively the possibility of an attack or blunt trauma, causing subserosal haemorrhages on the bladder) followed a strong flight reaction and capture myopathy might be considered. It is worthwhile to see if cardio respiratory clinical data can be evaluated to see if any signs of heart muscle pathology were observed. |
| PP130513 | • Skin: parakeratotic hyperkeratosis, locally extensive, marked, associated with bacterial and fungal (Fusarium sp.) infection.  • Icterus diffuse acute mild  • Subpleural hematoma focal chronic mild  • Esophagitis ulcerative multifocal chronic mild  • Stomach, pyloric: gastric abscess, focal, chronic, mild, associated with a trematode infection (probably Pholeter gastrophilus)  • Cardiac valve cyst focal chronic mild  • Keratitis bilateral chronic moderate  • Adrenal gland: Hypoplasia of the inner layer of the adrenal cortex, diffuse, chronic, marked, and replacement by erythrocyte-filled blood vessels.  • Cholangitis, multifocal, chronic, mild, associated with trematode infection  • Thyroid gland: cyst, solitary. |  | • Pulmonary oedema diffuse acute severe | This adult female in good nutritional condition was euthanized after six months in rehabilitation as she did not dive and only floated at the surface. The cause of this problem was not determined during necropsy. The most significant problem was a multifocal dermatitis caused by a Fusarium sp infection. In humans such an infection does occasionally disseminate and result in arthritis and osteomyelitis. Although such lesions were not observed they could have been missed during necropsy. The mild icterus and hepatic swelling may have resulted from the therapy with voriconazole or the high dosages of prednisolone which were administered. The keratitis may also have been caused by a Fusarium sp infection. The hypoplasia of the inner layer of the adrenal cortex could have been caused by a neoplasm elsewhere in the body producing sex steroids, as this would result in the zona reticularis to become hypoplastic. The zona reticularis is responsible for producing sex steroids. Alternatively, it might be a consequence of aging: teleangiectasis of the adrenal cortex, at the cortico-medullary border, with loss of adrenocortical cells, occurs in older animals. A third possibility is that it is a consequence of high prednisolone doses 1 to 2 months before euthanasia. |
| PP130829 | • Lung-associated lymph node, right: lymphoid hyperplasia, benign, marked.  • Liver: Cholangitis, proliferative, multifocal, chronic, marked, with fibrosis.  • Dermatitis, lymphocytic, diffuse, chronic, mild.  • Otitis diffuse chronic mild associated with Stenurus minor infection  • Bronchitis multifocal chronic mild associated with nematode infection  • Stomach, fundic: gastritis, lymphocytic, focal, chronic, mild, associated with parasite infection.  • Duodenal ampullae: enteritis, lymphocytic, multifocal, chronic, associated with parasite infection.  • Mesenteric lymph node: lymphadenitis, lymphocytic, focal, chronic, mild, associated with parasite infection.  • Oesophagitis ulcerative multifocal subacute moderate | • Lung: pulmonary abscesses, multifocal, chronic, marked, associated with Aspergillus fumigatus infection.  • Lung: bronchopneumonia, pyogranulomatous, coalescing, chronic, marked, probably associated with Aspergillus fumigatus infection.  • Palate: palatal abscess, focal, chronic, marked, associated with Aspergillus fumigatus infection. |  | This adult male in very poor nutritional condition died due to a chronic pneumonia. The most important lesions are those in the palate and lungs, caused by Aspergillus fumigatus infection. These were chronic lesions that would explain the emaciation of this animal. Dissemination from one location to the other by hematogenous spread or via the airways appears likely. It is surprising that breathing was normal upon acceptance into the rehabilitation centre, because he must already have had severe respiratory lesions then. Aspergillus fumigatus infection usually is secondary to some kind of immunosuppression. It is not clear what that was in this animal. |
| PP140204 | • Cerebellum: Purkinje cell degeneration, multifocal, acute, moderate (or artefact from, e.g., euthanasia?)  • Liver: hepatic lipidosis, diffuse, marked. |  | • Lung: pulmonary oedema, diffuse, peracute, moderate. (associated with euthanasia) | This neonatal harbour porpoise in poor nutritional condition was euthanized as a consequence of its poor survival chances. The most important lesion was the poor level of nutrition and complete absence of food remains in the gastrointestinal tract which indicate starvation was the main problem of this neonate. |
| PP140627 | • Epidermal lesions multifocal acute mild  • Ulcerative esophagitis multifocal acute mild  • Ulcerative gastritis multifocal acute mild  • Lung: bronchopneumonia, locally extensive, fibrinosuppurative, mild, acute.  • Liver: hepatic lipidosis, diffuse, moderate.  • Umbilicus: omphalitis, haemorrhagic, suppurative, acute, focal, mild.  • Tonsil: tonsillitis, suppurative, focal, superficial, acute, mild. |  | • Pulmonary oedema diffuse peracute marked (caused by euthanasia) | This female neonate harbour porpoise in moderate nutritional condition was euthanized upon arrival in the rehabilitation centre of SOS-Dolfijn. The most significant observations were absence of food remains or chillum in the mesenteric lymphatic vessels and the liver degeneration. The most likely cause of death in line with these observations is starvation. The liver degeneration may have caused the anorexia or might be the result of the anorexia. The multiple small ulcers observed in the gastrointestinal tract might have been the result of the chronic stress due to not feeding. These ulcers appear too small to have caused the anorexia. The inability to feed may have been the result of the mother animal not being available to the neonate. |
| PP140917 | • Oedematous lymphadenitis multifocal acute moderate (prescapular Lnn)  • Lymphopathy multifocal acute moderate (pulmonary Lnn)  • Lung: pulmonary oedema, diffuse, acute, mild to moderate.  • Hepatic lipidosis diffuse mild  • Prescapular lymph node: Lymphonodular oedema, diffuse, acute, marked.  • Lung-associated lymph node: Lymphoid hyperplasia, benign, marked. | • Lung: bronchopneumonia, suppurative, multifocal, acute to subacute, moderate to marked, associated with bacterial infection.  • Malnutrition (no nourishment) chronic  • Skin of snout: Dermatitis, suppurative, necrotizing, locally extensive, superficial, acute, marked, associated with bacterial infection |  | This neonatal male harbour porpoise in poor nutritional condition was euthanized as the length indicated this animal was unfit for release. The most significant findings were a bronchopneumonia and a marked locally extensive dermatitis of the snout. The oedematous lymphadenitis of the prescapular lymph nodes and the enlarged pulmonary lymph nodes were consequential to the above-mentioned infections. |
| PP150210 | • Ventricular septal defect, congenital  • Pneumonia, granulomatous, multifocal, chronic, mild.  • Pulmonary oedema, locally extensive, acute, moderate.  • Lung-associated lymph node: lymphoid hyperplasia, benign.  • Ascites acute diffuse mild  • Thyroid hypoplasia or thyroid follicular atrophy, marked.  • Dermal trauma acute multifocal mild  • Dermal hyperplasia focal chronic mild  • Duodenum: enteritis, granulomatous, multifocal, chronic, mild.  • Mesenteric lymph node: lymphadenitis, granulomatous, eosinophilic, multifocal, chronic, mild  • Epidermal keratin plug, focal. |  |  | This female harbour porpoise in good nutritional condition was euthanized after 11 days in the rehabilitation centre. The most significant lesion was a ventricular septal defect. It is unclear if the observed pulmonary oedema was due to heart failure or to euthanasia. During life no signs were observed which indicate heart failure, no fluid in thorax or abdomen, normal to low breathing frequency. The pulmonary associated lymph nodes were enlarged, indicating possibly a pneumonia which could otherwise not be supported by any other observations. |
| PP151116 | . • Pericardial effusion mild  • Persistent ductus botallicus chronic mild  • Mesenteric lymph node: Lymphonodular necrosis, multifocal, acute, moderate. | • Tail vertebra: Puncture wound with suppurative osteitis, focal, acute, superficial, mild.  • Skin: Puncture wounds with suppurative dermatitis, multifocal, acute to subacute, superficial, mild  • Emaciation severe  • Lung: Bronchopneumonia, fibrinopurulent, unilateral, locally extensive, acute, moderate. | • Pulmonary oedema peracute diffuse severe associated with euthanasia or agony | This juvenile female porpoise in extremely poor nutritional condition was euthanized because of poor prospects for recovery. The main findings were emaciation and multiple trauma suspected to be caused by a grey seal attack. The retracted wound edges and bulging tissue indicate this trauma was caused approximately one to a few days before the animal stranded. Tens of small otoliths were observed in the fore stomach otherwise the digestive tract was near empty. Harbour porpoises of this length/age at this time of year still nurse and are mother dependent A possible sequence of events is then: loss of mother leading to loss of nutrition leading to emaciation and decrease of fitness and immune resistance with subsequent grey seal attack and bronchopneumonia. The bite wounds caused an osteomyelitis of the underlying vertebra. |
| PP160304 | • Lung, right cranial lung lobe: multifocal mild subacute granulomatous bronchopneumonia with intralesional nematodes  • Lung, near left bronchus: focal extensive mild subacute pyogranulomatous bronchopneumonia with intralesional nematodes  • Lung: left and right lung: multifocal mild subacute suppurative bronchopneumonia with intralesional bacteria  • Skin, tailstock and fluke: multifocal moderate subacute necropurulent dermatitis, due to suspect bite marks  • Liver: multifocal (random) moderate acute hepatocellular necrosis  • Skeletal muscle: focal mild subacute skeletal muscle necrosis  3) Incidental diagnoses:  • Tail, vertebral column: mild scoliosis  • Skin: focal mild chronic pyogranulomatous furunculosis  • Skin: multifocal epidermo-pathy  • Liver: multifocal mild chronic lymphoplasmacytic cholangitis | General: Emaciation  • Kidney: suspect protein loss |  | This severely emaciated juvenile female harbour porpoise showed very little autolytic changes. The lacerations and punctures in the skin of the tail fluke, pectoral fins and the tailstock were bilaterally present, often mirroring, with regular distances between them and very suggestive of (grey) seal bite wounds. The inflammation in the tissues adjacent to the wound seemed subacute (hyperaemia and suppurative exudate present, but little hyperplasia of epidermis or fibrosis) so could have been inflicted around the time of stranding. The irregular surface of the body wall seemed to be due to slight fluctuation of the thickness of the epidermis. The cause for this is not clear, but possibly related to drying of the skin due to the stranding. The skin on the right lateral body wall was different, and showed wrinkling in cranio-caudal direction, and might also be related to drying (on what side was the animal found?).  A focal area of muscle necrosis was detected macroscopically and microscopically. Not the complete longissimus dorsi seemed to have been affected, it is not clear how much muscle was affected. The reported (clinical history) increased CK and LDH in the blood suggest that muscle damage was more widespread than the one focus detected. Within kidney cortex, both in glomerular tufts and in tubular lumina, was proteinaceous material. This could fit with the expected myoglobinuria, or with (more generalized) proteinuria (additional stains should be performed for definite diagnosis). The protein loss due to damage of the glomeruli (due to the myoglobinuria, if it occurred) might have caused the quick loss of weight and the hypoalbunemia.  The cause for the liver necrosis and the skin lesions (epidermo-pathy) are not clear. |
| PP160331 | • Nematode infection of bronchioles  • Lung artery: Nematode infection.  • Focal dermal trauma healed (scar tissue) mild  • Nodular hyperplasia of the gastric epithelium focal chronic mild |  | • Lung: Pulmonary oedema, locally extensive, acute, moderate. | This juvenile female harbour porpoise in good nutritional condition died shortly after she was found. Gross necropsy and histology did not provide clues to why this animal stranded. The only slightly unusual observation is that nearly all tissues had marked dilatation of small blood vessels, which were filled with erythrocytes. This suggests a widespread vasodilatation, which might cause hypovolemic shock (but what would cause such shock is unclear). In conclusion the necropsy was unsatisfactory in that it did not provide a cause of death of this animal. |
| PP160527 | • Semi-obstructive nematode infection of the pulmonary vasculature multifocal marked chronic (Pseudalius inflexus)  • Bilateral infestation of the ears with Stenurus minor  • Lung: Pneumonia, granulomatous, multifocal, chronic, mild, associated with nematode infection.  • Nematode infection of the right heart chamber mild chronic  • Oesophageal erosion, focal, mild  • Mesenteric lymph node: Lymphonodular granuloma, focal, chronic, marked.  • Gastric erosion, focal, acute, mild. | • Cerebral meninges: Meningitis, lymphocytic, diffuse, mild.  • Otitis media, necrotizing, suppurative, diffuse, chronic-active, marked, associated with Aspergillus sp. | • Pulmonary oedema (due to euthanasia) diffuse marked peracute | This juvenile male harbour porpoise in very poor nutritional condition was euthanized after four days in the rehabilitation centre. The most important finding was a unilateral middle ear infection which continued into the cerebral meninges and was associated with Aspergillus hyphae. |

**Table 2** **Organs involved and etiological categories involved in significant diagnoses, per stranded harbour porpoise.**

|  | Organ involved in significant diagnoses | | | | | | | | | | | | | | | | | | | |  | Other probable causes of stranding | | | |  |
| --- | --- | --- | --- | --- | --- | --- | --- | --- | --- | --- | --- | --- | --- | --- | --- | --- | --- | --- | --- | --- | --- | --- | --- | --- | --- | --- |
| Erasmus code number | Lung | | Liver | Brain | | Integument | | Kidney | Muscle | Skeleton | Pancreas | Heart | Ear | | Pharynx | Oesophagus | Stomach | Vasculature | Lymph nodes | Eyes | Sepsis | | Orphaned neonate | Emaciated^#^ | Unknown |  |
| PP030405 |  | |  |  | |  | |  |  |  |  |  |  | |  |  |  |  |  |  |  | |  |  |  |  |
| PP040324 |  | |  |  | |  | |  |  |  |  |  |  | |  |  |  |  |  |  |  | |  |  |  |  |
| PP040517 |  |  |  |  | |  | |  |  |  |  |  |  | |  |  |  |  |  |  |  | |  |  |  |  |
| PP040526 |  |  |  |  | |  | |  |  |  |  |  |  | |  |  |  |  |  |  |  | |  |  |  |  |
| PP041215 |  |  |  |  | |  | |  |  |  |  |  |  | |  |  |  |  |  |  |  | |  |  |  |  |
| PP050208 |  |  |  |  | |  | |  |  |  |  |  |  | |  |  |  |  |  |  |  | |  |  |  |  |
| PP050502 T | **Alv Prot** | |  |  | |  | |  |  |  |  |  |  | |  |  |  |  |  |  |  | |  |  |  |  |
| PP050610 T |  | |  |  | |  | |  |  |  |  |  |  | |  |  |  |  |  |  |  | |  |  |  |  |
| PP050825.1 |  | |  |  | |  | |  |  |  |  |  |  | |  |  |  |  |  |  |  | |  |  |  |  |
| PP050825.2 |  | |  |  | |  | |  |  |  |  |  |  | |  |  |  |  |  |  |  | |  |  |  |  |
| PP051106 |  | |  |  | |  | |  |  |  |  |  |  | |  |  |  |  |  |  |  | |  |  |  |  |
| PP060227 |  | |  |  | |  | |  |  |  |  |  |  | |  |  |  |  |  |  |  | |  |  |  |  |
| PP060327.2 |  |  |  |  | |  | |  |  |  |  |  |  | |  |  |  |  |  |  |  | |  |  |  |  |
| PP060327.3 |  | |  |  | |  | |  |  |  |  | **NON** |  | |  |  |  |  |  |  |  | |  |  |  |  |
| PP060501 |  | |  |  | |  | |  |  |  |  |  |  | |  |  |  |  |  |  |  | |  |  |  |  |
| PP061122.1 |  |  |  |  | |  | |  |  |  |  |  |  | |  |  |  |  |  |  |  | |  |  |  |  |
| PP061122.2 |  | |  |  | |  | |  |  |  |  |  |  | |  |  |  |  |  |  |  | |  |  |  |  |
| PP061123.1 | **NON** | |  |  | |  | | **NON** |  |  |  |  |  | |  |  |  |  |  |  |  | |  |  |  |  |
| PP070221 |  |  |  |  | |  | |  |  |  |  |  |  | |  |  |  |  |  |  |  | |  |  |  |  |
| PP070328 |  |  |  |  | |  | |  |  |  |  |  |  | |  |  |  |  |  |  |  | |  |  |  |  |
| PP080403 |  | |  |  | | **NON** | |  |  |  |  |  |  | |  |  |  |  |  |  |  | |  |  |  |  |
| PP080701 |  | |  |  | |  | |  |  |  |  |  |  | |  |  |  |  |  |  |  | |  |  |  |  |
| PP081222 |  |  |  |  | |  | |  |  |  |  |  |  | |  |  |  |  |  |  |  | |  |  |  |  |
| PP090626 |  | |  |  | |  | |  |  |  |  |  |  | |  |  |  |  |  |  |  | |  |  |  |  |
| PP110329 |  |  |  |  | |  | |  |  |  |  |  |  | |  |  |  |  |  |  |  | |  |  |  |  |
| PP110711 |  | |  |  | |  | |  |  |  |  |  |  | |  |  |  |  |  |  |  | |  |  |  |  |
| PP120906.1 |  | |  |  | |  | |  |  |  |  |  |  | |  |  |  |  |  |  |  | |  |  |  |  |
| PP120906.2 |  |  |  |  | |  | |  |  |  |  |  |  | |  |  |  |  |  |  |  | |  |  |  |  |
| PP120906.3 |  |  |  |  | |  | |  |  |  |  |  |  | |  |  |  |  |  |  |  | |  |  |  |  |
| PP121031 |  |  |  |  | |  | |  |  |  |  |  |  | |  |  |  |  |  |  |  | |  |  |  |  |
| PP121102 |  |  |  |  | |  | |  |  |  |  |  |  | |  |  |  |  |  |  |  | |  |  |  |  |
| PP121130T |  | |  |  | |  |  |  |  |  |  |  |  | |  |  |  |  |  |  |  | |  |  |  |  |
| PP130829 |  | |  |  | |  | |  |  |  |  |  |  | |  |  |  |  |  |  |  | |  |  |  |  |
| PP140917 |  | |  |  | |  | |  |  |  |  |  |  | |  |  |  |  |  |  |  | |  |  |  |  |
| PP151116 |  | |  |  | |  | |  |  |  |  |  |  | |  |  |  |  |  |  |  | |  |  |  |  |
| PP160527 | **WSD** | |  |  | |  | |  |  |  |  |  |  | |  |  |  |  |  |  |  | |  |  |  |  |
| PP030320 | **WSD** | |  |  | |  | |  |  |  |  |  |  | |  |  |  |  |  |  |  | |  | **EMA** |  |  |
| PP030919 | **WSD** | |  |  | |  | |  |  |  |  |  |  | |  |  |  |  |  |  |  | |  |  |  |  |
| PP031124.2 | **WSD** | |  |  | |  | |  |  |  |  |  |  | |  |  |  |  |  |  |  | |  | **EMA** |  |  |
| PP040627 | **WSD** | |  |  | |  | |  |  |  |  |  |  | |  |  |  |  |  |  |  | |  | **EMA** |  |  |
| PP060220 | **WSD** | |  |  | |  | |  | **NON** |  |  | **NON** |  | |  |  |  |  |  |  |  | |  |  |  |  |
| PP060301 | **WSD** | |  |  | |  | |  |  |  | **NON** |  |  | |  |  |  |  |  |  |  | |  |  |  |  |
| PP060327.1 | **WSD** | |  |  | | **NON** | |  |  | **NON** |  |  |  | |  |  |  |  |  |  |  | |  |  |  |  |
| PP060327.4 | **WSD** | |  |  | |  | |  |  |  |  |  |  | |  |  |  |  |  |  |  | |  |  | **UNK** |  |
| PP060524 | **WSD** | |  |  |  |  | |  |  |  |  |  |  |  |  |  |  |  |  |  |  | |  |  |  |  |
| PP070317 | **WSD** | |  |  |  |  | |  |  |  |  |  |  |  |  |  |  |  |  |  |  | |  |  |  |  |
| PP100105 | **WSD** | |  |  |  |  | |  |  |  |  |  |  |  |  |  |  |  |  |  |  | |  | **EMA** |  |  |
| PP100928.1 | **WSD** | |  |  |  |  | |  |  |  |  |  |  |  |  |  |  |  |  |  |  | |  | **EMA** |  |  |
| PP100928.2 | **WSD** | |  |  |  |  | |  |  |  |  |  |  |  |  |  |  |  |  |  |  | | **ORN** |  |  |  |
| PP110228 | **WSD** | |  |  |  |  | |  |  |  |  |  |  |  |  |  |  |  |  |  |  | |  |  |  |  |
| PP110928.1 | **WSD** | | **NON** |  |  |  | | **NON** |  |  |  |  |  |  |  |  |  |  |  |  |  | |  |  |  |  |
| PP110928.2 | **WSD** | |  |  |  | **NON** | |  |  |  |  |  |  |  |  |  |  |  |  |  |  | |  |  |  |  |
| PP111219 | **WSD** | |  |  |  |  | |  |  |  |  |  |  |  |  |  |  |  |  |  |  | |  | **EMA** |  |  |
| PP120703 | **WSD** | |  |  |  |  | |  |  |  |  |  |  |  |  |  |  |  |  |  |  | | **ORN** |  |  |  |
| PP130204 | **WSD** | |  |  |  |  | | **NON** | **NON** |  |  |  | **NON** | |  |  |  |  |  |  |  | |  |  |  |  |
| PP130513 | **WSD** | |  |  |  |  | |  |  |  |  |  |  | |  |  |  |  |  |  |  | |  |  | **UNK** |  |
| PP140204 | **WSD** | |  |  |  |  | |  |  |  |  |  |  | |  |  |  |  |  |  |  | | **ORN** |  |  |  |
| PP140627 | **WSD** | |  |  |  |  | |  |  |  |  |  |  | |  |  |  |  |  |  |  | |  | **EMA** |  |  |
| PP150210 | **WSD** | |  |  |  |  | |  |  |  |  |  |  | |  |  |  |  |  |  |  | |  |  | **UNK** |  |
| PP160304 | **WSD** | |  |  | |  | | **NON** |  |  |  |  |  | |  |  |  |  |  |  |  | |  |  |  |  |
| PP160331 | **WSD** | |  |  | |  | |  |  |  |  |  |  | |  |  |  |  |  |  |  | |  |  | **UNK** |  |

**Legend for Tables 1, 2 and 3**

Yellow is Parasitic aetiology

Blue is Bacterial aetiology

Red is fungal aetiology

Grey is inflammation of unknown aetiology

ALP = Alveolar Proteinosis

NON = Non-inflammatory lesion

WSD= Lungs without significant diagnoses

L = 1-100 nematodes (both lungs

H = >100 nematodes (both lungs)

L/H= infection with unknown intensity

T = animal treated with anti parasitics

EMA= emaciated

ORN= Orphaned Neonate

UNK= No cause for stranding observed during necropsy

# emaciation was only severe lesion observed

**Table 3** **Nematode infections in juvenile harbour porpoises with and without severe pneumonia**

|  |  | |  | Nematode species | | |  |
| --- | --- | --- | --- | --- | --- | --- | --- |
| Erasmus code number | Pneumonia caused by | | Infectious organisms | *Stenurus minor* (<22 mm) | *Halocercus*  *invaginatus/ Torynurus convolutus* (30 - 70 mm) | *Pseudalius inflexus* (>100 mm) | age (days, est) |
| PP040324 |  | |  |  |  | **H** | 296 |
| PP050825.1 |  | |  |  | **H** | **H** | 275 |
| PP060501 |  | | Lungworm larvae |  |  |  | 325 |
| PP121130T |  | |  |  | **L** | **H** | 530 |
| PP050825.2 |  | | Small lungworms (susp *Sten. minor*) | **L/H** |  |  | 405 |
| PP120906.3 |  |  |  |  | **L** | **L** | 345 |
| PP041215 |  |  | Bact: *Aeromonas sp.* 2 species  Parasitic pneumonia: lungworm larvae |  | **H** | **H** | 561 |
| PP060327.2 |  |  | Bact: Coccoid bacteria |  | **H** | **L/H** | 286 |
| PP070221 |  |  | Bact: *Actinobacillus delphinicola* and *Brucella sp.* |  | **H** | **H** | 264 |
| PP111219 |  |  | Bact: *E. coli* and *S. maltophilia* |  | **H** |  | 197 |
| PP120906.2 |  |  | Bact: *Streptococcus dysgalactiae* |  | **H** | **H** | 372 |
| PP040517 |  |  | Fung: *Aspergillus fumigatus*  Parasitic pneumonia: lungworm larvae. | **L** | **L** | **L** | 317 |
| PP061122.1 |  |  | Fung: *Aspergillus fumigatus* |  | **L** |  | 353 |
| PP030405 |  | | Bact: 4 species 3  specified: *Pseudomonas aeruginosa Streptococcus sp. Escherichia coli* |  |  |  | 647 |
| PP061122.2 |  | | Bact: *Escherichia coli* |  | **L** | **L** | 490 |
| PP070328 |  | | Bact: mixed coliforms |  | **H** |  | 299 |
| PP080701 |  | |  |  |  |  | 294 |
| PP140917 |  | | No growth |  |  |  | 76 |
| PP151116 |  | | No growth |  |  |  | 137 |
| PP120906.1 |  | | Fung: *Aspergillus fumigatus* |  |  | **L** | 147 |
| PP050610 T |  | | Fung: *Aspergillus fumigatus* |  | **L (dead worms)** |  | 325 |
| PP080403 |  | | No growth |  | **H** |  | 306 |
| PP110711 |  | | Bact: *Brucella ceti* susp |  | **H** |  | 1132 |
| PP160527 | **WSD** | |  | |  |  | 331 |
| pp030320 | **WSD** | |  | | **H** |  | 291 |
| PP030919 | **WSD** | |  | |  |  | 104 |
| PP031124.2 | **WSD** | |  | |  |  | 282 |
| PP060301 | **WSD** | |  | | **L** | **L** | 266 |
| PP060327.1 | **WSD** | |  | |  |  | 283 |
| PP060327.4 | **WSD** | |  | |  |  | 253 |
| PP060524 | **WSD** | |  | |  |  | 357 |
| PP061123.1 | **NON** | |  | |  |  | 52 |
| PP100105 | **WSD** | |  | | **L/H** |  | 215 |
| PP100928.1 | **WSD** | |  | |  |  | 49 |
| PP100928.2 | **WSD** | |  | |  |  | 37 |
| PP110228 | **WSD** | |  | |  | **L** | 264 |
| PP110928.1 | **WSD** | |  | |  |  | 17 |
| PP110928.2 | **WSD** | |  | | **L** |  | 678 |
| PP130204 | **WSD** | |  | |  |  | 216 |
| PP140627 | **WSD** | |  | |  |  | 28 |
| PP150210 | **WSD** | |  | |  |  | 240 |
| PP160304 | **WSD** | |  | | **L** |  | 273 |
| PP160331 | **WSD** | |  | | **L** | **L** | 272 |

**Legend for Table 3 see underneath Table 2**

**Table 4** **Nematode infections in adult harbour porpoises with and without severe pneumonia**

|  |  |  | Nematode species | | |
| --- | --- | --- | --- | --- | --- |
| Erasmus code number | Pneumonia caused by | Infectious organisms | *Stenurus minor* (<22 mm) | *Halocercus*  *invaginatus/ Torynurus convolutus* (30 - 70 mm) | *Pseudalius inflexus* (>100 mm) |

| PP051106 |  | |  |  | **H** | **H** |
| --- | --- | --- | --- | --- | --- | --- |
| PP090626 |  | |  |  | **H** | **L** |
| PP110329 |  |  | Bact: *Actinobacillus delph*inicola |  | **H** | **H** |
| PP121031 |  |  | Bact: *Salmonella sp.* (host adapted group B *Salmonella*) |  |  | **H** |
| PP081222 |  |  | Bact: *Pseud. aeruginosa* |  | **L/H** | **L/H** |
| PP050208 |  |  | Fung: *Aspergillus fumigatus* | **L** | **L** |  |
| PP121102 |  | | Fung: *Aspergillus fumigatus* |  | **L** | **H** |
| PP130829 |  | | Fung: *Aspergillus fumigatus* |  | **L** | **L** |
| PP060227 |  | |  |  |  | **L** |
| PP060327.3 |  | |  |  |  |  |
| PP050502 T | **AL P** | |  |  |  |  |
| PP060220 | **WSD** | |  |  | **H** | **L** |
| PP070317 | **WSD** | |  | **L/H** | **L/H** | **H** |
| PP130513T | **WSD** | |  |  |  |  |
|  |  | |  |  |  |  |

**Legend for Table 4, see under Table 2**

**Table 5** ***p*-values according to Fisher's exact test (two-sided) comparing nematode infections in juvenile harbour porpoises with severe pneumonia to those in juveniles without severe pneumonia (***n* = **20).**

|  | Nematode species | | |
| --- | --- | --- | --- |
|  | *Stenurus minor* | Mix *Torynurus convolutes* and *Halocercus sp.* | *Pseudalius inflexus* (bronchi, lung) |
| Juveniles with severe pneumonia (*n* = 21/22)* | *p* ≥ 0.47 | *p* ≤ 0.008 | *p* ≤ 0.002 |
| Juveniles with a severe parasitical pneumonia (incl. combined parasitic-fungal-bacterial infections) (*n* = 13) | *p* ≥ 0.36 | *p* ≤ 0.003 | *p* ≤ 0.00006 |
| Juveniles with a severe parasitic pneumonia (*n* = 5/6)^*^ | *p* = 0.2 | *p* ≤ 0.17 | *p* = 0.005 |
| Juveniles with a severe bacterial pneumonia (incl. combined bacterial and parasitic infections) (*n* = 12) | *p* ≥ 0.27 | *P* = 0.008 | *p* ≤ 0.006 |
| Juveniles with only a severe bacterial pneumonia (*n* = 5) | *p* = 1.0 | *p* >0.54 | *p* = 0.25 |
| Juveniles with a severe fungal pneumonia (incl. mixed fungal and parasitic infections) (*n* = 3/4)* | *p* = 0.45 | *p* = 0.007 | *p* = 0.00009 |

Values indicate the outcome of the test comparing the sample mentioned in the left-hand row to the sample of animals without pneumonia. The top left value, for example, tells us that the statistical chance that juveniles with a pneumonia and without a pneumonia came from a population with the same prevalence and intensity of *Stenurus minor* infection is 0.47.

Larger or smaller than p values indicate animals with unknown number of parasites were in the test. For these animals’ calculations were done with all possible combinations of light or heavy infections. (in red: *p* < 0.05 = significant difference in lungworm infection compared to animals without pneumonia).

Animals which have received anti-parasitic treatment were excluded. * one anti-parasiticum treated juvenile animal with a light infection of dead worms (mix *Torynurus convolutes/ Halocercus sp.*) This single result was taken into consideration for the statistical analysis and tested as an infection of unknown intensity.

All untreated adult animals with parasitical (*n* = 2), bacterial (*n* = 3), fungal (*n* = 3) or without pneumonia (*n* = 2) had light or heavy infections with both *Torynurus convolutus/Halocercus sp.* and *Pseudalius inflexus.* Apart from one animal with a bacterial infection where no *Torynurus convolutus* or *Halocercus sp.* was observed. Statistical analysis comparing animals with and without pneumonia in relation to parasite load, was therefore not informative. For *Stenurus minor* infections infestation in animals without pneumonia was higher compared to animals with bacterial or fungal pneumonia.

**Table 6** **Comparison of prevalence and abundance of *Pseudalius inflexus* infections in the pulmonary vasculature of juveniles and adult harbour porpoises.**

|  |  |  | No. of animals (percentage of animals) with estimated parasite abundance of: | | | |
| --- | --- | --- | --- | --- | --- | --- |
| Age category | No. of animals infected (prevalence of infection) |  | 1 – 10 | 11 – 100 | 101 – 1000 | Unknown |
| Juveniles (*n* = 41) | 19 (46%) |  | 2 (10%) | 10 (50%) | 2 (10%) | 5 (25%) |
| Adults (*n* = 14) | 10 (71%) |  | 4 (40%) | 1 (10%) | 2 (20%) | 3 (30%) |

**Table 7** **Comparison of prevalence of gastrointestinal parasites in juvenile and adult harbour porpoises.**

|  | Parasite species and location of infection | | | | |
| --- | --- | --- | --- | --- | --- |
| Age category | *C. oblongata*  Liver | *C. oblongata*  Pancreas | *A.Simplex*  Fore stomach | *P. Gastrophilus*  Fundic stomach | *D. stemmacephalum*  Intestines |
| Juveniles (*n* = 41) | 5 | 0 | 2 | 4 | 1 |
| Adults (*n* = 13) | 6 | 1 | 4 | 0 | 0 |
| P values | **0.02** | 0.24 | **0.02** | 0.56 | 1 |

Numbers are numbers of animals

*P* values according to fisher test. *P*< 0.05 indicates significant difference in prevalence

**Table 8** **Comparison of prevalence and abundance of different parasite species in the digestive tracts of juvenile and adult harbour porpoises**

|  | No. of animals (percentage of animals) infected, per organ and corresponding parasite species | | | | |
| --- | --- | --- | --- | --- | --- |
| Age category | Pancreas (*Campula oblonga*) | Liver (*Campula oblonga*) | Forestomach (*Anisakis simplex*) | Pyloric stomach (*Pholeter gastrophilus*) | Intestine (cestode, likely *Diphyllobothrium stemmacephalum*) |
| Juveniles (*n* = 41) | 0 (0%) | 5 (12 %)^u^ | 2 (5%)^l^ | 4 (10%)^u^ | 1 (2%)^l^ |
| Adults (*n* = 13) | 1 (8%)^u^ | 6 (46%)^u^ | 4 (31%)^h^ | 0 (0%) | 0 (0%) |

^u^ parasite abundance unknown

^l^ parasite abundance light

^h^ parasite abundance high

**Table** **9 Overview of lesions, diagnosis and most prominent clinical signs.**

| Erasmus code number | Organs affected by lesions which contributed to stranding, emaciation, suspected aetiology | diagnosis | Most prominent clinical signs | Other and or remarkable signs |
| --- | --- | --- | --- | --- |
| PP030320 | Emaciation |  | Tachypnoea (16/min), hypothermic, vomiting |  |
| PP030405 | Lungs | Pyogranulomatous pneumonia multifocal chronic severe, eyes (corneal perforation) | Nervous: bumped into wall |  |
| PP030919 | Integument | dermatitis, fibrinosuppurative, superficial, diffuse, acute, marked, with bulla formation | increase in temperature, intermittent vomiting and increase in breathing frequency | Animal developed acute bullae on dorsal side of body |
| PP031124.2 | Emaciation |  | Vomiting, passive unable to keep afloat |  |
| PP040324 | brain, lungs | Bronchopneumonia, histiocytic, multifocal, chronic-active, marked, suppurative, associated with lungworm infection 1. meningitis, non-suppurative, segmental, chronic, moderate (cerebrum) 2. neuronal necrosis, segmental, moderate (cerebrum) | increasing tachypnoea |  |
| PP040517 | lungs, brain metastases suspected based on symptoms | 1. Bronchopneumonia, multifocal, chronic-active, moderate, suppurative, associated with lungworm larvae 2. pneumonia, necrotizing, focal, acute, mild, | Nervous: epileptiform attacks |  |
| PP040526 | lungs | Interstitial pneumonia, suppurative, multifocal, acute, moderate | respiratory; bradypnea, respiratory arrest |  |
| PP040627 | susp reaction to fish porridge no sign path observed |  | Abdominal: severe continuous cramping with a sharp rise in BF |  |
| PP050208 | lungs | Interstitial pneumonia, suppurative, multifocal, acute, moderate | Unable to dive |  |
| PP050502 T | lungs, kidneys, integument | 1. Bronchitis, lympho-plasmacytic, diffuse, subacute, moderate, suppurative, 2. Pneumonia, histiocytic, multifocal, chronic, moderate to severe, with abundant foamy material 3. nephritis, suppurative, focal, acute, mild containing small round pink bodies about 0.5 μm diameter (alveolitis) 4. Dermatitis, multifocal, suppurative, superficial, acute, moderate, associated with bacterial infection | Kidney failure as was evident by marked increase of urea, creatinine and Sodium, brown urine, **loss of body weight and anorexia** |  |
| PP050610 | lungs, muscles, heart, skeleton | 1. Lung: bronchopneumonia, necrotizing, haemorrhagic, granulomatous, multifocal or diffuse, chronic, marked 2. Pleura: pleuritis, granulomatous, multifocal or diffuse, moderate or marked. 3. Right and left heart ventricle: epicarditis and myocarditis, granulomatous, necrotizing, multifocal, chronic, moderate. 4. Vertebra (tail stock): osteomyelitis, pyogranulomatous, focal, chronic, marked, with exophytic bone formation. | High BF, high PR, clear infect resp tract |  |
| PP050825.2 | lungs, brain | 1. Bronchopneumonia, pyogranulomatous, multifocal, chronic, associated with nematode infection (probably Stenurus minor). 2. Encephalitis, lymphocytic, locally extensive, subacute, with neuronal necrosis and intranuclear inclusion bodies (herpesvirus). |  | She lifts her head out of the water and the respiratory muscles make a large effort to exhale. Gastric stasis occurs and at day 7 she starts to vomit |
| PP060220 | muscles, heart | 1. Right ventricular wall thinning. 2. skeletal muscle degeneration, diffuse, subacute, marked. | heavy cramps and forced breathing in final stage | spasms as from receiving an electric shock, disoriented swimming (bumping against the wall) and sporadic vomiting |
| PP060227 | brain, lungs | 1. Bronchopneumonia, suppurative, locally extensive, acute, moderate. 1. Cerebrum: encephalitis, lymphocytic, diffuse, subacute, moderate, with multifocal gliosis, perivascular cuffing, and oedema. 2. Cerebellum: meningo-encephalitis, lymphocytic, subacute, moderate, with multifocal gliosis and loss of Purkinje cells. 3. Cervical spinal cord: myelitis, lymphocytic, diffuse, subacute, moderate. | lethargic curved position, final morning sharp increase BF, agitated. |  |
| PP060301 | pancreas, emaciation | pancreatic duct hyperplasia/ occlusion, emaciation | Initially none, in final stage vomiting and increased BF |  |
| PP060327.1 | musculoskeletal trauma | musculoskeletal trauma |  |  |
| PP060327.2 | lungs | Pneumonia, multifocal, chronic, moderate, pulmonary abscesses associated with large and small nematodes |  |  |
| PP060327.3 | lungs, liver, cardiac | 1. Interstitial pneumonia, histiocytic, locally extensive, chronic, moderate, associated with unknown organisms. 2. Tracheo-bronchitis, lymphoplasmacytic, diffuse, subacute, marked, suppurative, superficial. 3. Hepatitis, necrotizing, multifocal, acute, marked. 4. Right ventricular wall thinning. | sporadic vomiting, arching, less active | Ronchi in left bronchi |
| PP060327.4 | Brain suspected |  | • Increased breathing frequency • Vomiting • Stasis of gastric content • Exudate occasionally with blood expired from the blowhole • Body tremor • Cramps • Forceful difficult exhalation (appears to choke) |  |
| PP060524 | brain, ear | 1. Dura mater: pachymeningitis, necrotizing, suppurative, locally extensive, acute, marked, associated with fungal hyphae (Aspergillus sp.). 2. Cerebellum: panencephalitis, pyogranulomatous, haemorrhagic, necrotizing, locally extensive, associated with fungal hyphae (Aspergillus sp.) and mixed bacterial infection. 3. Otitis media purulent subacute to chronic diffuse severe. | At first the animal appeared to swim uncoordinated with the tail making a cork screw type of motion. The pupil reflex of the right eye was slow and a slight vertical nystagmus of the right eye was visible. |  |
| PP061122.1 | lungs | 1. Bronchopneumonia, multifocal, chronic, mild to moderate, pyogranulomatous, 2. Bronchopneumonia, multifocal, chronic, moderate, pyogranulomatous, necrotizing | Increased BF, pulse, Vomiting. Blood loss from blowhole (once) and melaena, emaciated and very anaemic. Occasional exaggerated breathing movements |  |
| PP061122.2 | lungs, sepsis | 1. Bronchopneumonia, multifocal, subacute to chronic, severe, suppurative. 2. Myositis, suppurative, multifocal, acute, moderate (consistent with *Escherichia coli* infection). 3. Fasciitis, suppurative, focal, acute, moderate (consistent with *Escherichia coli* infection). | initially active and with good appetite afterwards listing, varying appetite, lethargy | slightly elevated BF (22 - 28/5min) |
| PP070221 | lungs | 1. Bronchopneumonia, multifocal, lymphocytic and eosinophilic, chronic, moderate, associated with nematode infection. 2. Bronchopneumonia, multifocal, suppurative, acute, moderate, associated with bacterial infection. | slight tachypnoea 9/min to 6/min a few hours after arrival | slight tachypnoea 9/min to 6/min a few hours after arrival |
| PP070317 | liver | 1. Hepatic necrosis, periacinar, extensive, acute, marked associated with a moderate icterus 2. Hepatic lipidosis, diffuse, acute, moderate | At first inactive and floating, then swimming but not diving on final day diving. Low body temperature. Final day vomiting. | severe leukopenia final two days |
| PP070328 | lungs | Bronchopneumonia, diffuse, acute, moderate, necrotizing, suppurative, associated with bacterial infection (mixed coliforms). | Increased BF (died during transport) |  |
| PP080403 | lungs, integument | 1. Bronchopneumonia, locally extensive, acute, marked, suppurative (too acute to explain emaciation). 2. Severe trauma to blowhole and eyelids (necessitating euthanasia) | none noted apart from the observation that blowhole could not be closed |  |
| PP080701 | lungs, sepsis, liver, pancreas | 1. Interstitial pneumonia, diffuse, acute, marked, haemorrhagic, (post rehab) 2. Pleuritis bilateral focally extensive fibrinopurulent acute moderate (post rehab) 3. Pneumonia, lymphohistiocytic, locally extensive, chronic, marked. 1. Liver: bile duct hyperplasia, diffuse, marked. 2.Pancreatic angitis, suppurative, necrotizing, chronic-active, multifocal, marked 3. Pancreatic duct hyperplasia, focal, moderate. | No clinical signs apart from increasing liver enzymes during rehab. Final day anorexia, vomiting, coughing (blood), fever, increased BF |  |
| PP100105 | lungs (in reh acquired), emaciation, muscles | Bronchopneumonia, diffuse, chronic-acute, marked, suppurative, granulomatous, associated with mixed bacterial and yeast-like (?) infection and aspiration of food remains marked. 2. Muscle degeneration, multifocal, acute, moderate. | tachypnoea, squeaks on auscultation, dark urine high bun while normal creatinine |  |
| PP100928.1 | emaciation |  |  |  |
| PP100928.2 | emaciation |  | drop in albumin and tp despite hand rearing formula, increasing weakness and lethargy |  |
| PP110228 | lungs (acquired in rehab), oesophagus | 1. Oesophagitis ulcerative multifocal to confluescent acute marked 2. Pneumonia, diffuse, (per) acute, mild, suppurative, associated with microthrombi. | Passive, blood loss upon expiration with vomitus and with defaecation |  |
| PP110711 | lungs, liver, emaciation | 1. Pneumonia, pyogranulomatous, locally extensive, chronic marked. 2. Hepatocytic degeneration, random, multifocal, acute, moderate, with neutrophil infiltration and hepatocytic necrosis. | tachypnoea (7 -8,5/ min average at times extr high) Signs of resp distress (head out of water tail bending) forced audible gurgling breathing. Elevated temp (38 to 38,3) lack of appetite, moderate leukopenia |  |
| PP110928.1 | liver, kidney | 1. Hepatocytic degeneration, random, multifocal, acute, moderate, with neutrophil infiltration and hepatocytic necrosis. 2. Renal tubular epithelial cell vacuolation, diffuse, marked. | defence musculaire, vomiting when given food, | bloodwork indicates kidney failure |
| PP111219 | lungs (aq in rehab), lungs, emaciation | 1. Bronchopneumonia, haemorrhagic, suppurative, diffuse, acute, marked, associated with bacterial infection, suspected aspiration pneumonia 2. Lung: alveolitis, pyogranulomatous or granulomatous, multifocal, chronic, mild or marked, associated with nematode infection | final day diff moist breathing, final hours sharp increase in breathing rate 18/min |  |
| PP120906.1 | lungs, brain | 1. Pneumonia, multifocal to coalescing, chronic, marked, pyogranulomatous. 2. Encephalitis, pyogranulomatous, multifocal, chronic, moderate. | dyspnoea with forced laboured breathing with gurgling sounds and exudate and accompanied by body turns (curved cramps) to the right side. Breathing frequency was increased | sporadic vomiting and white and red bits of exudate sporadically upon expiration |
| PP120906.3 | lungs, liver, brain | 1. Interstitial pneumonia, suppurative, histiocytic, locally extensive, chronic, moderate, suppurative 2. bronchopneumonia, granulomatous, multifocal, chronic, moderate. 3. Liver: hepatitis, necrotizing, multifocal, acute, marked. 4. Cerebrum: polioencephalitis, multifocal, mild. | The last day the animal showed less to absent appetite, had severe hypothermia (33 degrees Celsius) swam disorientated against the wall and had laboured difficult breathing with vertical body rises from the water for inspiration. | Blood values on the last day (only) showed increase in WBC, drop in Ht and Hgb and a slight hypernatremia. |
| PP121102 | lungs | Pneumonia, multifocal, chronic, marked, granulomatous | Passive low BF, very frequent coughing, passive only mild inflammatory markers rise in blood work, difficult breathing | Ronchi in bronchi (left side mainly) |
| PP121130T | lungs, vasculature, skin | 1. Bronchopneumonia, locally extensive, chronic, moderate, pyogranulomatous. 2. Forestomach, jejunum, colon: vasculitis, leukoclastic, haemorrhagic, multifocal, chronic-active, marked. 3. Dermatitis, pyogranulomatous, multifocal, chronic, marked, with epidermal hyperplasia, keratin pearls, and bacterial infection. | Last 48 hours passive and stiff, gained one kilo (ascites possibly) lowered breathing rate. | High to very high WBC not reacting to antibiotic therapy |
| PP130204 | kidney, bladder, muscles, heart | 1. Kidney: urolithiasis, mild, 2. subserosal haemorrhages on urinary bladder. 3. muscle degeneration, locally extensive, acute to subacute, marked. 4. Right heart: myocardial oedema, multifocal, acute, mild. | passive, strong defence musculaire, regular vomiting |  |
| PP130513 | unknown | unknown | Unable to swim or dive throughout its six months stay in the rehab centre. Necropsy did not provide an explanation for this sign. |  |
| PP130829 | lungs, pharynx | 1. Lung: pulmonary abscesses, multifocal, chronic, marked, associated with Aspergillus fumigatus infection. 2. Lung: bronchopneumonia, pyogranulomatous, coalescing, chronic, marked, probably associated with Aspergillus fumigatus infection. 3. palatal abscess, focal, chronic, marked. | Mucous exudates, and gurgling moist sounds with respiration, multiple abdominal cramps |  |
| PP140204 | emaciation |  |  |  |
| PP140627 | emaciation |  |  |  |
| PP140917 | lungs, skin | 1. Bronchopneumonia, suppurative, multifocal, acute to subacute, moderate to marked. 2. Skin of snout: Dermatitis, suppurative, necrotizing, locally extensive, superficial, acute, marked. |  |  |
| PP150210 | heart (but not reason for stranding), unknown | Ventricular septal defect, congenital | vomiting, constipation, coughing, leucocytosis |  |
| PP151116 | lungs, skin, bones, emaciation | 1. Bronchopneumonia, fibrinopurulent, unilateral, locally extensive, acute, moderate. 2. Puncture wounds with suppurative dermatitis, multifocal, acute to subacute, superficial, mild. 3. Tail vertebra: Puncture wound with suppurative osteitis, focal, acute, superficial, mild. |  |  |
| PP160304 | kidney (protein loss), emaciation | Approximately half of the glomeruli have a distended capsule of Bowman, which is filled with protein. Many cortical tubuli also are filled with granular eosinophilic material. | shaking when out of water, vomiting and continuous weight loss |  |
| PP160331 | none | none | Increased cardiac frequency (160/min no resp arrythmia), drop in albumin and TP despite good food intake, with a rise in Na and Cl indicating kidney failure and or vomiting (although not observed). Final day swimming into wall and unable to maintain upright position. |  |
| PP160527 | meninges, ear, emaciation | 1. Otitis media, necrotizing, suppurative, diffuse, chronic-active, marked. 2. Cerebral meninges: Meningitis, lymphocytic, diffuse, mild. | Increased cardiac frequency (160/min no resp arrythmia), drop in albumin and TP despite good food intake, with a rise in Na and Cl indicating kidney failure and or vomiting (although not observed). Final day swimming into wall and unable to maintain upright position. |  |
