# Supplementary figures and images for: Clinical, pathological, and laboratory diagnoses of diseases of harbour porpoises (Phocoena phocoena), live stranded on the Dutch and adjacent coasts from 2003 to 2016
Source: Vet Res. 2019 Oct 30;50:88. doi: 10.1186/s13567-019-0706-3 (PMC6822343; doi:10.1186/s13567-019-0706-3)

Number of admissions per year variation according to season (A), gender (B) and age class (C)

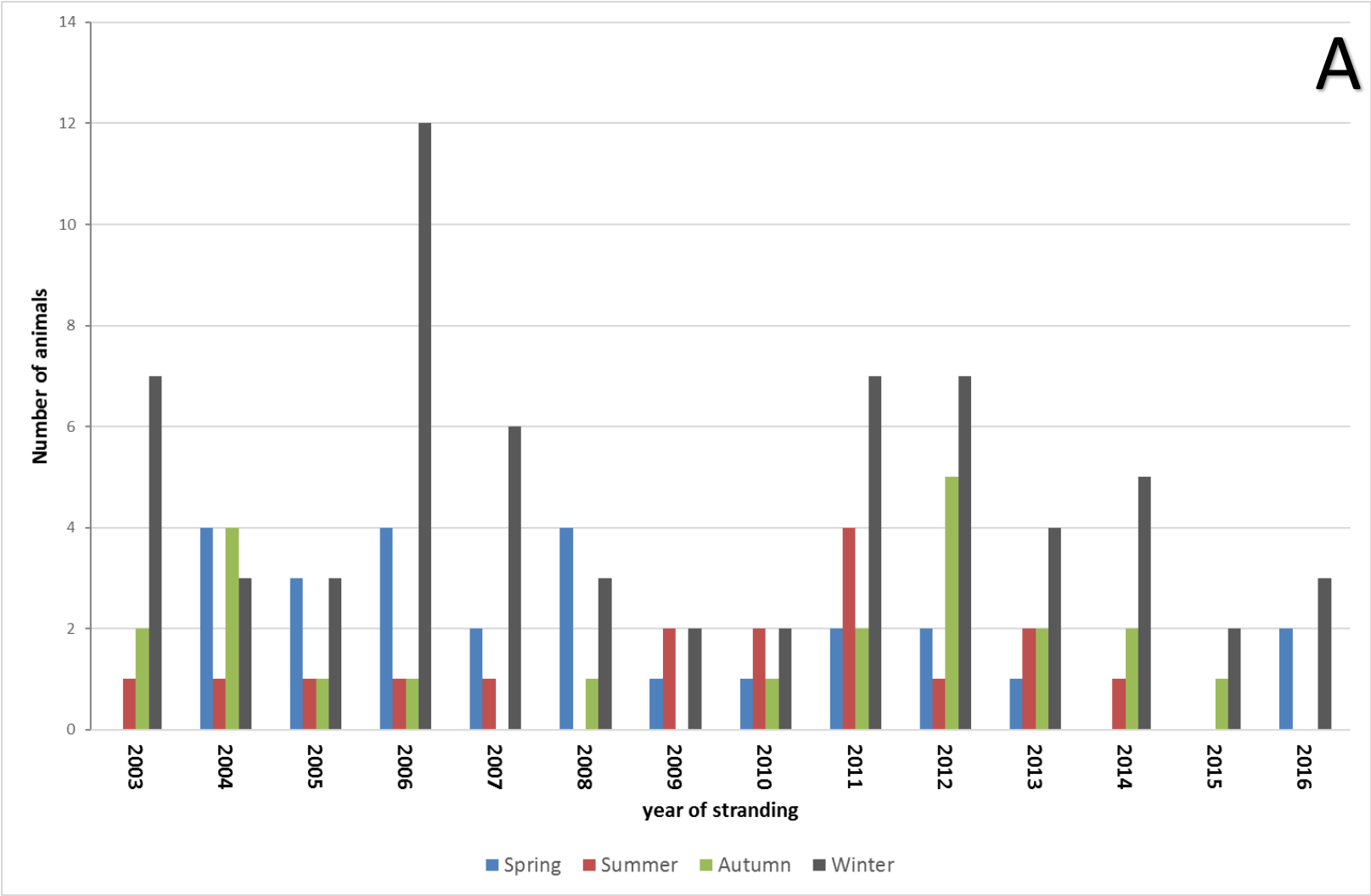

**B**

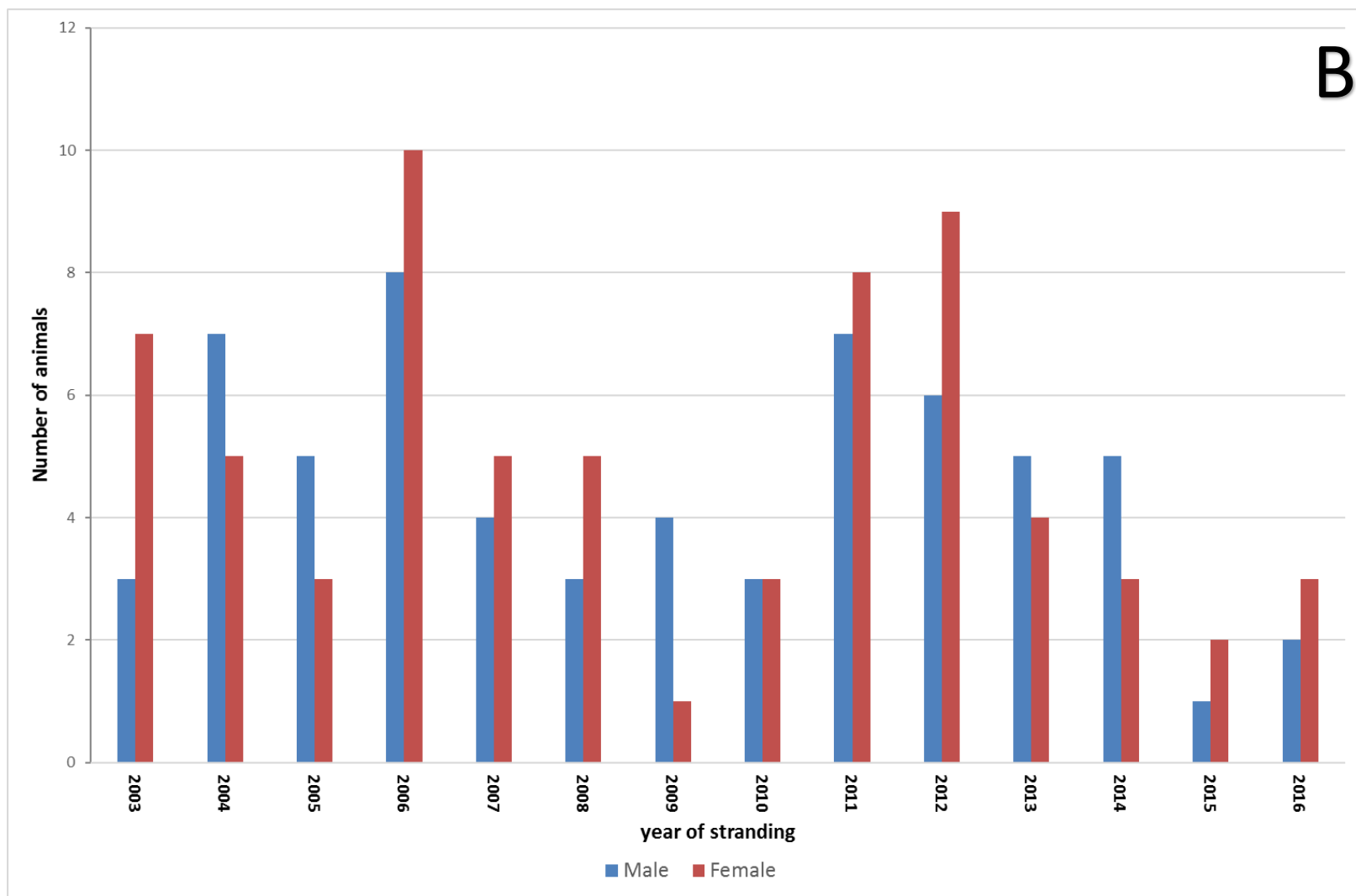

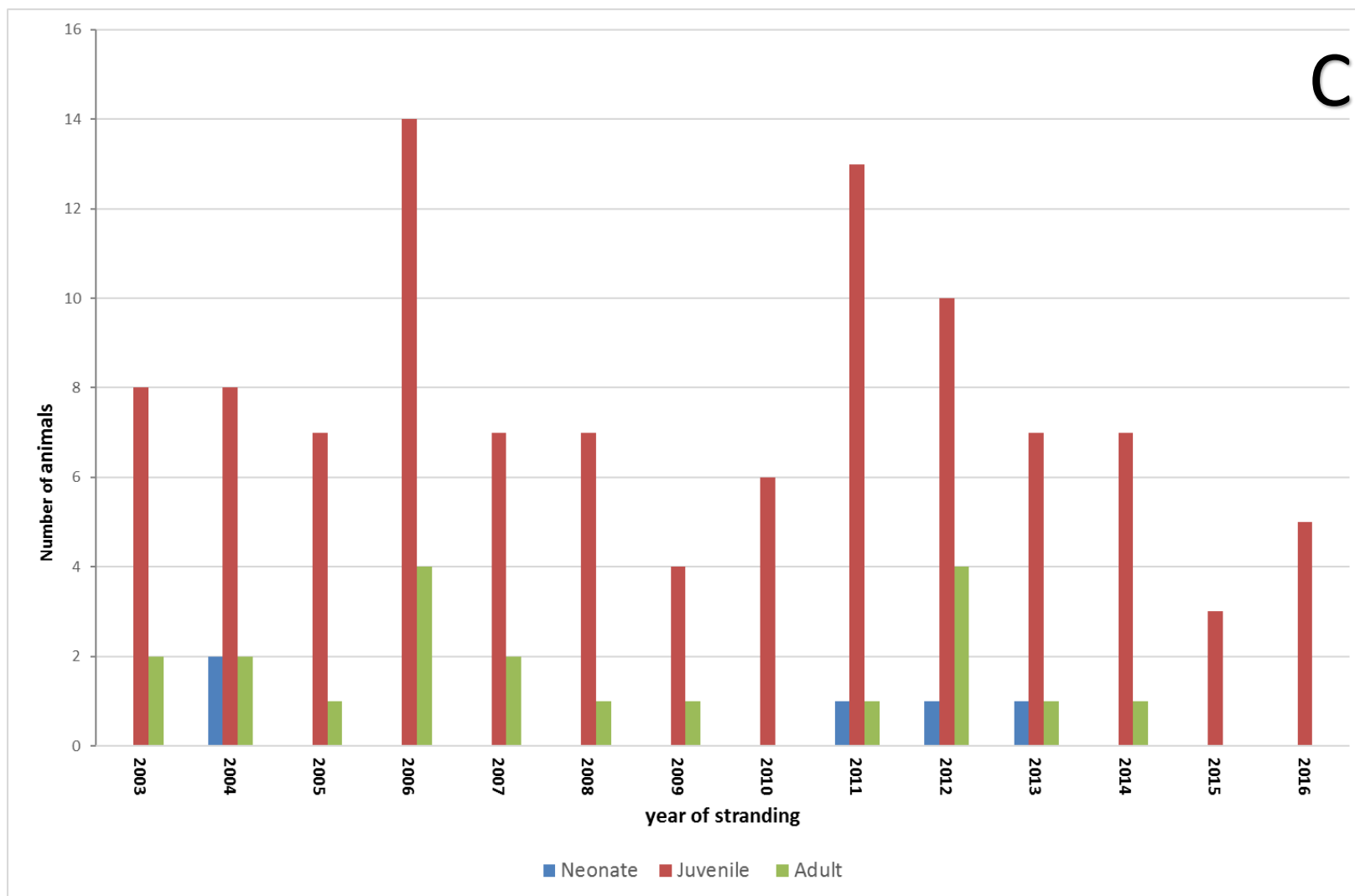

Supplement: Supplementary file 2 — Additional file 2. Number of annual admissions according to season, age class and gender. [file 13567_2019_706_MOESM2_ESM.pdf]
